# Population Structure of Genotypes and Genome-Wide Association Studies of Cannabinoids and Terpenes Synthesis in Hemp (*Cannabis sativa* L.)

**DOI:** 10.3390/plants15020202

**Published:** 2026-01-08

**Authors:** Marjeta Eržen, Andreja Čerenak, Tjaša Cesar, Jernej Jakše

**Affiliations:** 1 Department for Plants, Soil and the Environment, Slovenian Institute of Hop Research and Brewing, Cesta Žalskega Tabora 2, 3310 Žalec, Slovenia; andreja.cerenak@ihps.si; 2 Department for Agronomy, Biotechnical Faculty, University of Ljubljana, Jamnikarjeva 101, 1000 Ljubljana, Slovenia; tjasa.cesar@bf.uni-lj.si (T.C.); jernej.jakse@bf.uni-lj.si (J.J.)

**Keywords:** *Cannabis sativa*, genotype, GWAS, hemp, population structure, SNP

## Abstract

Hemp (*Cannabis sativa* L.) is one of the oldest cultivated plants in the world. It is a wind-pollinated and heterozygous species, and diverse phenotypes can occur within population varieties. In our study, three different hemp varieties—(‘Carmagnola Selected’ (CS), ‘Tiborszallasi’ (TS) and ‘Finola selection’ (FS))—were grown. Based on visual characteristics, two, five and four phenotypes were identified within CS, TS and FS, respectively. According to *Cannabis sativa* L. transcriptome data from the Sequence Read Archive (SRA), 4631 single-nucleotide polymorphism (SNP) positions were identified to develop capture probes. DNA was isolated from 171 plants representing selected phenotypes of three cultivars. Next-generation sequencing (NGS) libraries were constructed and hybridized with capture probes for target enrichment. The population structure of the samples was analyzed using SNP data for each genotype. Based on genotype profiles, CS formed a single cluster, while TS and FS were each grouped into two clusters, with phenotypes randomly distributed among them. The GWAS results were visualized using Manhattan plots. Fourteen significant SNPs surpassing the false discovery rate (FDR) of 0.01 were identified for delta-9-tetrahydrocannabinol (delta-9-THC). For cannabigerol (CBG), 12 significant SNPs were detected, and for myrcene, one SNP exceeded the 0.01 FDR threshold. However, plausible genes located 1000 bp to the left and right of the SNP position were identified for all significant SNPs.

## 1. Introduction

Hemp (*Cannabis sativa* L.) originates from Asia [1]. It contains over 500 phytochemicals and exhibits numerous therapeutic effects on human health [2,3]. Cannabinoids are the most prominent compounds, followed by essential oils.

The most well-known cannabinoids are delta-9-tetrahydrocannabinol (delta-9-THC), cannabidiol (CBD), cannabigerol (CBG) and cannabichromene (CBC). The controversy surrounding *Cannabis sativa* L. stems from the intoxicating effects produced by delta-9-THC. However, delta-9-THC also provides significant health benefits [3,4,5,6,7,8].

The most well-known components of *Cannabis sativa* L. essential oils are myrcene, limonene, α-pinene and β-caryophyllene, all of which also have positive effects on human health [9]. In addition, these terpenes contribute to the plant’s aroma and have insect-repellent properties [10]. Together with cannabinoids, they are considered to exert a synergistic effect on human health [2].

According to different cannabinoid ratios, five major chemotypes have been defined:

Chemotype I: High tetrahydrocannabinol (THC) content (>0.3%, >0.5%) and low cannabidiol (CBD) content (<0.5%);
Chemotype II: Intermediate type with a THC:CBD ratio around 1:1;
Chemotype III: High CBD content with THC <0.5%;
Chemotype IV: Dominated by cannabigerol (CBG) content;
Chemotype V: Cannabinoids undetectable [11,12].

Different genetic loci are associated with each chemotype. Plants with a B_T_B_T_ allele (where B_T_ is the THC-A synthase allele) exhibit Chemotype I. Chemotype II plants carry a B_T_/B_D_ allele (B_D_ being the CBD-A synthase allele). Chemotype III has a B_D_/B_D_ allele. Chemotype IV is presumed to have a B_0_ (null) allele encoding a non-functional synthase gene [13,14,15]. Chemotype V is defined by the genotype o/o-A pe^1−n^/A pe^1−n^ [14]. Laverty et al. (2019) constructed physical and genetic maps for drug-type Purple Kush and the Finola hemp-type variety [16]. They discovered that a THC-A-like synthase discovered by Kojoma et al. (2002) may encode a CBC-A synthase [17]. It was also found that drug-type plants contain only one copy of THC-A synthase and no copies of CBD-A synthase, while hemp-type contains only CBD-A synthase and no copies of THC-A synthase. Both varieties have genes encoding CBC-A synthase. The THC-A and CBD-A synthase genes are located at chromosome 7 of the CBDRx genome [18].

Cannabinoid precursors are synthesized via two different biosynthetic pathways: the polyketide pathway, which leads to olivetolic acid (OLA), and the plastidal 2-C-methyl-D-erythritol 4-posphate pathway, which produces geranyl diphosphate (GPP). Geranylpyrophosphate: olivetolate geranyltransferase catalyzes the alkylation of OLA with GPP, forming cannabigerolic acid (CBG-A) via a prenyltransferase. CBG-A is the direct precursor of tetrahydrocannabinolic acid (THC-A), cannabidiolic acid (CBD-A) and cannabichromenic acid (CBC-A) through their respective synthases [19].

*C. sativa* has a diploid genome with nine pairs of autosomes and one pair of sex chromosomes (X and Y). Genomic resources for *Cannabis sativa* L. are publicly available in the NCBI database resulting from several worldwide genomic projects [16,18,20].

The first draft genome and transcriptome of drug-type Purple Kush were presented by van Bakel et al. (2011) [21]. Numerous studies have aimed to examine genetic variability among *Cannabis sativa* L. types [22,23,24]. In the previous study, it was observed that some samples identified as *Cannabis sativa* subsp. *indica* resembled *Cannabis sativa* subsp. *sativa* and vice versa. Hemp-type exhibited a higher level of heterozygosity than drug-type, suggesting a broader genetic base [25]. These findings suggest that some hemp-type cultivars may be genetically closer to *indica* than *sativa* [23,25]. Soler et al. (2017) [25] used 20 feminized varieties of *Cannabis sativa* var. *indica* and two varieties of *Cannabis sativa* var. *sativa* (Finola and Futura) to study genetic diversity using genomic simple sequence repeat (gSSR) markers developed from the Purple Kush cultivar. High polymorphism was observed, averaging 17 alleles per locus, for determination of genetic structures, diversity, and relationship within *C. sativa* var. *indica* [25]. In 2021, Grassa et al. (2021) [18] assembled a high-quality, chromosome-level genome of the CBD-type cultivar CBDRx, with an ancestry of 89% drug-type and 11% hemp-type. It is highly collinear with chromosomal assemblies for Purple Kush and Finola but has more anchored genes and fewer contigs with higher contig values [18].

*Cannabis sativa* L. research was long hindered by restrictive legislation. However, in the past two decades, advances in high-throughput sequencing and SNP platforms have facilitated genomic analyses of *Cannabis sativa* L. aimed at cultivar improvement [26]. A comprehensive review on biotechnology and genetic engineering in *Cannabis sativa* L. was conducted by Hesami et al. (2020) [27]. Johnson and Wallace (2021) investigated the genetic and phenotypic consistency of high-CBD hemp-type using genotype-by-sequencing (GBS) rather than shotgun sequencing [28]. GBS was also used in the study by Soorni et al. (2017) to compare *Cannabis sativa* L. samples from two germplasm collections [29], while Adamek et al. (2023) compared GBS with simple sequence repeats (SSRs) in micropropagated plants [30]. Many studies are performed with the goal to identify genetic differences between *C. sativa* cultivars [17,22,25,29,31,32,33]. However, there is only one study that examined differences within drug-type varieties but only to a limited extent [34].

Building on the data of Eržen et al. (2021) [35], the presented work aimed to develop a high-throughput genotyping method. The goal was to investigate genomic variation among different phenotypes within three hemp-type varieties, with reference to known traits for 10 cannabinoids, 29 components of essential oils and six morphological traits as well as differences between selected genotypes at a genetic level [35].

## 2. Results

### 2.1. Phenotype Determination

Two phenotypes were observed within CS (CI, CII), five were observed within TS (TI, TII, TIII, TIV, TV), and four were observed within FS (FI, FII, FIII, FIV) (Table 1). Detailed photos of the phenotypes are provided in Supplementary Collection of Figure S1.

### 2.2. SNP Position Selection from Transcriptomic Data

Among the three analyzed *Cannabis sativa* L. varieties, ‘Purple Kush’ exhibited the highest number of polymorphisms, with 782,406 SNPs identified. In contrast, ‘Santhica 27’ had the lowest number of polymorphisms (122,033 SNPs). The ‘Finola’ variety showed the highest number of homozygous SNPs (149,967), whereas ‘Santhica 27’ and ‘Purple Kush’ exhibited the highest number of biallelic SNPs (62,443 and 461,557, respectively) (Table 2).

The highest number of polymorphisms was on chromosome 1, with an averge distribution of 67.5 kb/SNP regardless of the intersection. The lowest number of polymorphisms was on chomosome X, with a distribution of 131.3 kb/SNP. The highest SNP count was found in the Finola and Purple Kush intersection on chromosome 1 (635 SNPs), while the lowest was observed in the three-way intersection on chromosome 7 (67 SNPs) (Table 2). Based on the final set of 4631 annotated SNPs, capture probes for a total of 4537 SNP positions were designed. Among these, 4086 probes exhibited 100% coverage (three probes per SNP position), 227 probes had 60% coverage (two probes per SNP position), and 224 probes had 33% coverage (one probe per SNP position).

### 2.3. Population Structure

**Figure 1:**
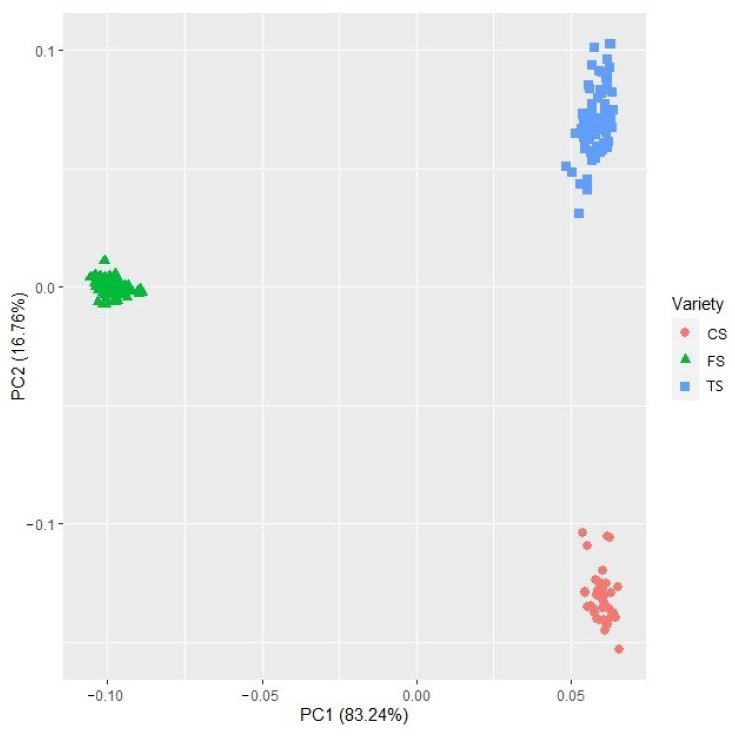
PCA plot of genotypic data of all three hemp varieties (Carmagnola selected (CS), Tiborszallasi (TS) and Finola selection (FS)) according to the first two components. First component, PC1, explained 83.24%, and second component, PC2, explained 16.76%.

After filtering the genomic data in Tassel, a total of 3670 high-quality SNPs were obtained. The proportion of missing data was 0.00563. The proportion of heterozygosity was 0.33703, and the minor allele frequency (MAF) was 0.28874. A PCA was conducted using genotypic data of all three hemp-type varieties (CS, TS and FS) based on the first five principal components. A PCA plot was generated using the first two components, which clearly distinguished the three varieties from one another (Figure 1). The first, PC1, explained 83.14% of the total genetic variance, while the second, PC2, accounted for 16.76%. Phenotypes within individual varieties determined by visual traits were not distinguished.

**Figure 2:**
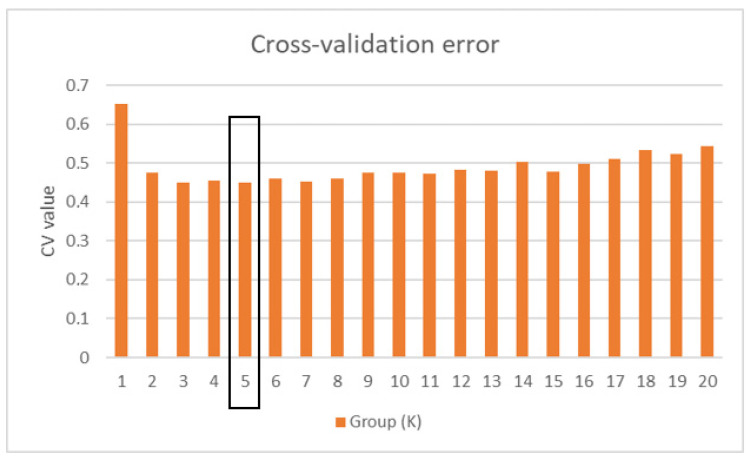
Groups of cross-validation for cluster detection using Admixture. The black frame indicates the lowest CV value.

The Admixture analysis grouped the genotypes from the CS, TS and FS varieties into five genetic clusters (K = 5) based on the CV error, which reached its minimum at 0.44940 (Figure 2). The CS variety appeared genetically homogeneous, showing no distinguishable substructure among its phenotypes. In contrast, both TS and FS varieties were subdivided into two distinct clusters, though their phenotypes were randomly distributed across the genetic groups.

**Figure 3:**
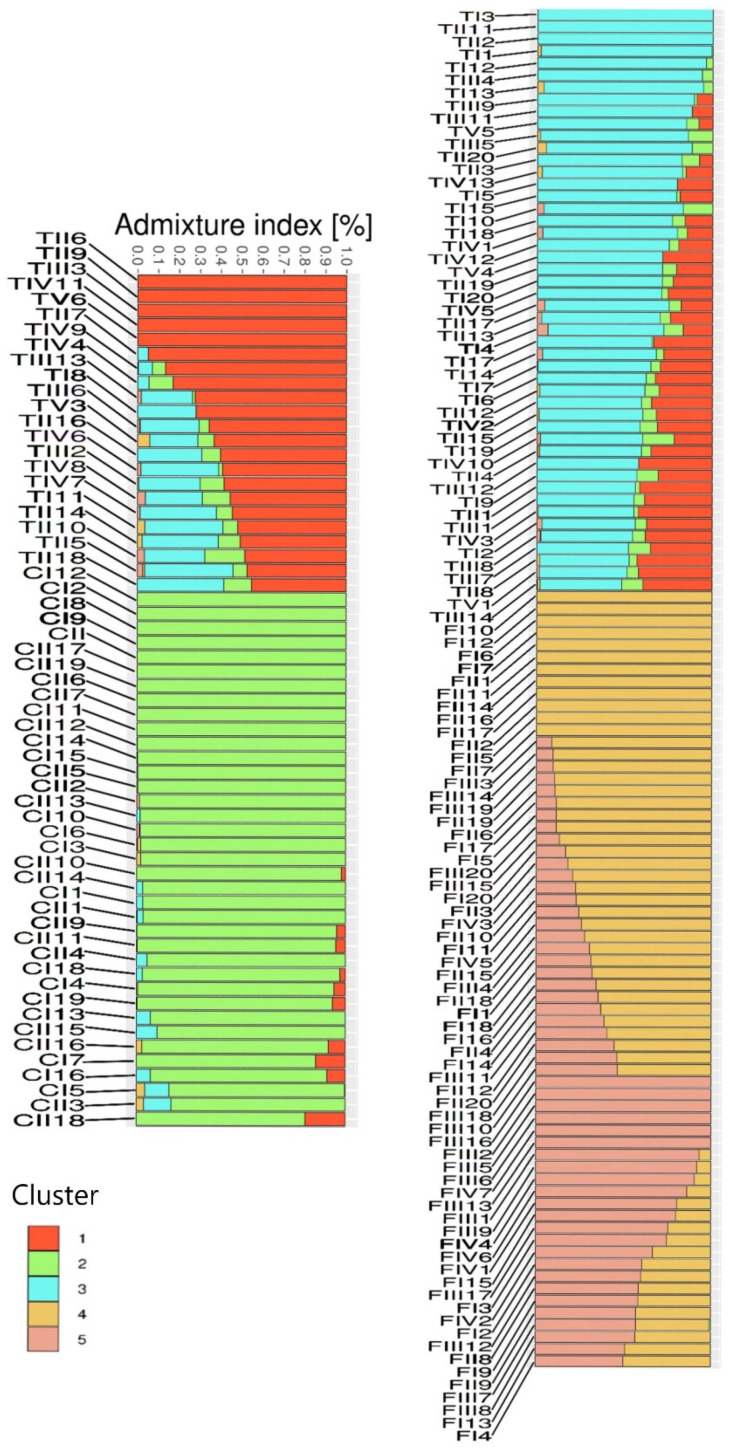
Population structure of three different hemp varieties (‘Carmagnola selected’ (CI–CII), ‘Tiborszallasi’ (TI–TV) and ‘Finola selection’ (FI–FIV)) with associated phenotypes.

As illustrated in Figure 3, there is evidence of genetic similarity between the CS and TS varieties. The TS genotypes share minor components of cluster 2, which is predominant in CS, while the CS genotypes display slight contribution from clusters 1 and 3, more common in TS. The FS variety, however, is genetically distinct from both CS and TS, as it is composed exclusively of clusters 4 and 5, indicating minimal genetic overlap.

### 2.4. Genome-Wide Associations (GWAS)

To determine the association between genetic variation and phenotypic traits, a set of 3670 high-quality SNPs was combined with data for 45 phenotypical traits. These traits encompassed cannabinoid and terpene profiles as well as visually assessed morphological traits and were determined in 55 hemp-type samples used in previous research [35].

**Figure 4:**
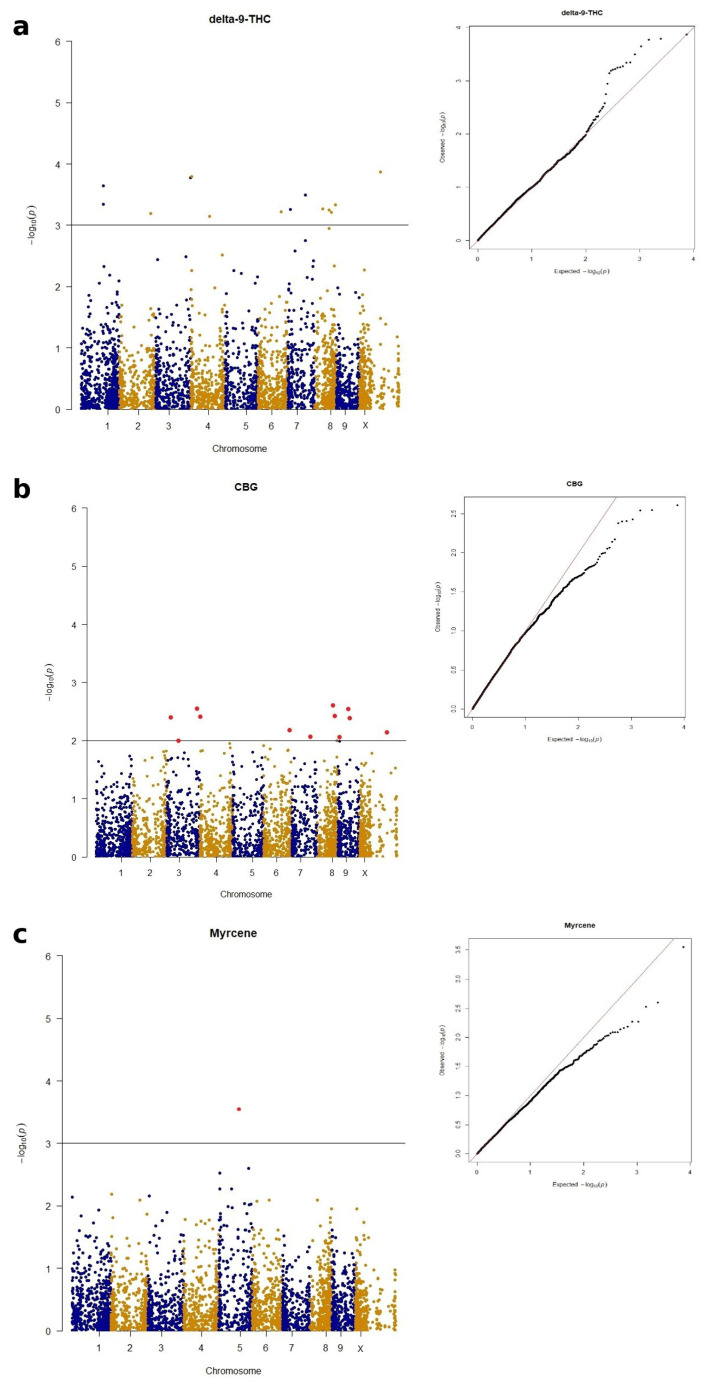
Manhattan plots showing significant SNP positions (red dots) above the FDR threshold across the 10 *Cannabis* chromosomes and Q-Q plot for delta-9-THC (**a**), CBG (**b**) and myrcene (**c**) traits.

For the MLM analysis, phenotypic and genotypic data were analyzed using a mixed linear model that included principal components (PCA) to account for population structure, as shown in Figure 4a–c and Supplementary Figure S1–S42.

A total of 14 significant SNPs were associated with delta-9-THC (Figure 4a). These SNPs were distributed across multiple chromosomes: two on chromosome 1; one each on chromosomes 2, 3 and 6; two on chromosome 4; two on chromosome 7; four on chromosome 8 and one on chromosome X, which was also the most significant SNP, with a *p*-value of 0.000135 and an R^2^ of 0.3474. For each significant SNP, putatively associated genes were identified (Supplementary Table S3). There were four uncharacterized genes on chromosomes 3, 8 and 10. RNA-binding protein 24-B and a conserved oligomeric Golgi complex subunit 4, similar to that associated with protein transfer, are located on chromosome 1. Spermidine coumaroyl-CoA acyltransferase, involved in metabolic pathways and mediating the conversion of sperimidine to dicoumaroyl-sperimidine, is located on chromosome 2. Nucleobase-ascorbate transporter 7, involved in the transport of organic substances, and 3-hydroxyacyl-[acyl-carrier-protein] dehydratase FabZ, linked to the biosynthesis of unsaturated fatty acids in bacteria, are located on chromosome 4. On chromosome 7 are located ethylene response sensor 1, involved in the negative regulation of the signaling pathway activated by ethylene, which is also involved in the biosynthesis of secondary metabolites, and an L-type lectin domain containing a receptor kinase S.4-like gene that plays a role in protein phosphorylation. On chromosome 8, three genes are located: a probable WRKY transcription factor 72, involved in the processes of plant growth, plant development and the stress response; putative methyltransferase DDB_G0268948, involved in methylation; and ketol-acid reductoisomerase (chloroplastic), involved in the valine biosynthetic process.

For CBG (Figure 4b) 12, significant SNPs were identified, located on the following chromosomes: three on chromosome 3; one each on chromosomes 4, 6 and 7; two on chromosome 8; three on chromosome 9 and one on chromosome X. The most significant SNP was located on chromosome 8, position 49352334, with a *p*-value of 0.00245. On chromosome 3, putatively associated 40S ribosomal protein S2-4, the ubiquitin-conjugating enzyme E2-23 kDa and an uncharacterized gene were identified. An uncharacterized gene was also identified on chromosome 4. On chromosome 6, zinc finger CCCH domain-containing protein 19, involved in gene silencing, is located. A putative lipase YDR444W is located on chromosome 7. The homeobox protein knotted-1-like LET6, which is expressed in the vegetative meristem of *Arabidopsis thaliana*, and lipid protein patellin-3, which can be found in secretion proteins and proteins regulated by lipides, are located on chromosome 8. Thioredoxin X, chloroplastic, U-box domain-containing protein 44 and CHUP1 and chloroplastic are located on chromosome 9. Ubiquitin carboxyl-terminal hydrolase 16, involved in the ubiquitin-dependent protein catabolic process, is located on chromosome X (Supplementary Table S3).

For myrcene (Figure 4c), one significant SNP was identified on chromosome 5, which was above the 0.01 FDR threshold. On this chromosome, putatively associated pre-mRNA-splicing factor ISY1 homolog was found, which generates a catalytic spliceosome for the second transesterification step (Supplementary Table S3).

## 3. Discussion

Genomic analyses have significantly improved cultivars of major crops in the past two decades, particularly through the application of high-throughput sequencing technologies and the availability of high-quality reference genomes and transcriptomic datasets [26]. One of the breakthroughs in *Cannabis sativa* L. genomics was the publication of the first draft genome of the Purple Kush variety [21].

The purpose of our study was to examine the genetic differentiation among individuals from three hemp-type varieties (CS, TS and FS), focusing on phenotypes selected within each variety. These three varieties were intentionally selected in order to focus on phenotypic and genetic variation within individual varieties. Because hemp is a highly heterogeneous crop, substantial phenotypic (visual) variation occurs among plants within the same variety. In this study, multiple distinct phenotypes were observed within each cultivar (e.g., five phenotypes in Tiborszallasi, four in the Finola selection and two in Carmagnola selected). Accordingly, the objective of this study was not to assess genetic diversity across a large number of cultivars but rather to investigate intra-varietal variation within three selected cultivars and to determine whether this variation is associated with visually observed phenotypic differences.

Captured reads were mapped to the cs10 reference genome, one of the most complete genomes based on the chromosome level to date, encompassing 25,302 protein-coding genes [36]. Using the Admixture software version 1.3.0, genetic data were grouped into five clusters: TS and FS genotypes were separated into two clusters each, while CS genotypes formed a single cluster. When comparing different phenotypes within our three varieties based on genotypic data using the PCA method, differences were shown between varieties, while phenotypes within each variety did not form distinct clusters. Comparable studies have been conducted at broader taxonomic levels. For instance, Gao et al. (2014) [22] used SSR markers to analyze population structure of 115 different varieties: a total of 100 from China and 15 from Europe. Varieties were divided into four clusters (three clusters from the Chinese varieties and one cluster from the European varieties) [22]. Henry et al. (2020) [37] used 23 SNPs related to cannabinoids, terpenoids, fiber and resin products of three different groups of *Cannabis* (*sativa*, *indica* and *ruderalis*). According to population structure samples, they were divided into five clusters. European hemp-types were classified as one group, K5. Drug-type resin accessions were classified as two groups, K1 and K3. *C. sativa* from the equator were classified as group K4, and high CBD resin accessions were classified as the K2 cluster [37]. The genetic structure of *C. sativa* and *C. indica* was studied by Sawler et al. (2015) [23]. They used 14,031 SNPs from 81 *indica* and 43 *sativa* types. They discovered that these two types are significantly different on a genetic level, whereby differences are not limited only to THC synthase genes. They also discovered that heterozygosity is higher in *C. sativa* than in *C. indica*, which indicates that hemp-types come from a wider genetic pool than drug-types [23]. Zhang et al. (2020) also conducted genetic research using SSR markers to study genetic diversity and the population structure of *Cannabis sativa* L. from different parts of the world [33]. Chen et al. (2022) [38] performed genome resequencing of different Chinese *Cannabis sativa* L. plants. Chinese *Cannabis sativa* L. could be divided into five clusters according to geographical origin and ecotype [38].

Our results highlight genetic differentiation among the three varieties studied, especially the genetic distinctness of ‘Finola selection’, supporting the potential for cross-breeding between genetically distant varieties. This could result in higher heterozygosity and potentially a higher content of desirable cannabinoids or components of essential oils. GWAS is a powerful tool to uncover the association between genotype and phenotype, whereby differences in the allele frequencies of genetic variants are considered [39]. While GWAS studies in *Cannabis sativa* L. are still limited, a few have been conducted on *Cannabis sativa* L. based on agronomic traits [40,41]. Our study is the first comprehensive GWAS to examine a wide range of phenotypic traits, including cannabinoids, terpenes and visual traits within and across hemp-type varieties.

We analyzed 3537 high-quality SNPs in relation to 45 phenotypic traits and generated Manhattan plots for all traits. For 12 other phenotypic traits, candidate genes associated with significant SNP positions were identified (Supplementary Table S3). For example, the CBD-A trait was associated with a berberine-bridge enzyme-like 15 gene found on chromosome 7 (Supplementary Table S3). This is consistent with previous studies indicating that CBG-A is a precursor for cannabinoids, such as THC-A, CBD-A and CBC-A, and is synthesized via olivetolic acid and geranyl pyrophosphate synthase [42]. Both THC-A and CBD-A synthases are soluble enzymes, have 84% identical amino acid sequences and contain a domain homologous to the berberine-bridge enzyme (BBE) [43].

Interestingly, several genes were found to be associated with more than one cannabinoid. For instance, delta-9-THC and THC-A traits were both associated with RNA-binding protein 24-B and ethylene response sensor 1 on chromosome 1. RNA-binding proteins play an important role. They post-transcriptionally regulate the gene expression of different biological processes. They also play an important role in plant adaptation to abiotic stress [44]. Ethylene is responsible for fruit ripening, seed germination, cell elongation, senescence of plants and leaf fall. It is also a mediator for stress situations and pathogen infections. Ethylene actions are connected via receptors and sensors, such as ethylene response sensor 1 [45].

In addition, both CBD-A and delta-9-THC were associated with genes such as conserved oligomeric Golgi complex subunit 4-like and a transcription factor WRKY 72, which has a defense function. Transcription factors from the WRKY family were also found in hops, where they affect lupulin biosynthesis [46]. CBG and CBD shared significant SNPs near the 40S ribosomal protein S5 gene.

For α-pinene, a significant SNP was associated with taumatin-like protein 1, which was found on chromosome 5. This protein acts like a powdery mildew resistance gene and provides resistance to other pathogens in hops and grape vines [47]. Synthase genes for cannabinoids, such as CBD-A, THC-A and CBC-A, are known to reside on chromosome 7 [18], while the CBG-A synthase gene is located on chromosome X [48]. Grassa et al. (2021) also studied gene expression of cannabinoid synthase of CBDRx (cs10) transcripts [18]. They reported that protein-coding genes were included in the cannabinoid’s synthesis and precursor pathways. In our study, with the exception of limonene on chromosome 1—where we identified a geranylgeranyl transferase type-1 subunit beta gene—no additional candidate genes overlapped with those identified by Grassa et al. (2021) [18]. Geranyl pyrophosphate, a precursor for both CBG-A and monoterpenes, is a key intermediate in both cannabinoid and terpene biosynthetic pathways. Overall, our GWAS results provide foundational datasets for future marker-assisted breeding in *Cannabis sativa* L.

## 4. Materials and Methods

### 4.1. Plant Material

Three different dioecious hemp-type (*Cannabis sativa* L.) varieties (Cannabaceae) were grown in 2019. ‘Carmagnola selected’ (CS) and ‘Tiborszallasi’ (TS) were cultivated in Ljubno ob Savinji on silty soil, and ‘Finola selection’ (FS) was grown in Žalec, Slovenia, on clay to clay–loamy soil. The experiment was initially designed to be conducted at the Ljubno ob Savinji location using two varieties (CS and TS). Subsequently, this study was expanded to include a third variety (FS). Due to spatial limitations at the original site, the expanded experiment was established at the Žalec location. The two sites are geographically close and were exposed to comparable climatic conditions and identical cultivation practices, thereby minimizing environmental and management-related variability. The cultivation area covered 1080 m^2^, with a row spacing of 75 cm at both locations. In Ljubno ob Savinji, the previous crop was corn, while in Žalec, it was hop. According to the visual traits assessed by human observation—including height, color, leaf size, compactness of inflorescences, anthocyanin coloration of leaf petiole and branching—different phenotypes were identified within each variety. Each plant was visually inspected based on these characteristics. Only female plants were included in the analysis. To prevent pollination, male plants were eliminated from the field prior to full flowering. In total, 171 samples were selected for further analysis. Plants were monitored throughout the growing season. Phenotypes were determined at the end of maturity. Leaves for genetic analysis were collected at the end of September and stored at −20 °C until further analysis.

### 4.2. Determination of SNP Positions from Transcriptomic Data

In the SRA database (Sequencing Read Archive), transcriptomic NGS data for ‘Finola’ (SRR351933, SRR7630403, SRR351932), ‘Purple Kush’ (SRR352210, SRR352208, SRR352205) and ‘Santhica 27’ (SRR5210008, SRR5209988, SRR5209953) were obtained. The selected data were mapped to NCBI’s cs10 reference genome (BioProject PRJEB29284, GCA_900626175.2) using CLC Genomic Workbench Version 20.0.4. Common SNP positions were determined for all three varieties using the Basic Variant Detection tool, which is part of the CLC Genomic Workbench. Common SNP positions to all three varieties were visualized using Venny software version 2.0. Only biallelic SNP positions were selected for further analysis.

To test the suitability of the SNP selection methodology for our samples, 60 SNP positions were chosen: 30 SNP positions common to all three varieties (‘Purple Kush’, ‘Finola’ and ‘Santhica 27’) and 30 SNP positions common to ‘Santhica 27’ and ‘Finola’. Positions were randomly selected across the chromosome. To ensure the low copy number of selected positions in the hemp-type genome, the BLASTn tool (https://blast.ncbi.nlm.nih.gov/Blast.cgi?PROGRAM=blastn&PAGE_TYPE=BlastSearch&LINK_LOC=blasthome (accessed on 1 March 2021)) was used on the cs10 reference genome. Positions with more than one significant alignment were excluded. After filtering, 14 primer pairs were designed using Primer3Web Version 4.1.0 (https://primer3.ut.ee/ (accessed on 20 March 2021)) (Table 3).

The primers were used to verify the adequacy of SNP selection in eight hemp-type samples using a PCR mixture of 15.1 μL deionized water, 2.5 μL 10x buffer PCR, 2 μL MgCl, 2 μL dNTP, 1.25 μL each of primer pair, 0.13 μL Taq polymeraze enzime and 0.75 μL DNA, with the following amplification program: 95 °C for 5 min, followed by five cycles where annealing temperature decreased by 1 °C each cycle (30 s) (60 °C, 59 °C, 58 °C, 57 °C, 56 °C). Amplification took place at 72 °C (1 min), followed by 30 cycles at 95 °C (30 s), 55 °C (30 s) and 72 °C (1 min).

Once PCR Sanger sequencing was completed, samples were cleaned using ExsoSAP-IT^TM^ (Aplied Biosistems^TM^ by Thermo Fisher Scientific, Los Angeles, CA, USA) and incubated in a thermal cycler. Denaturation took place at 95 °C for 3 min, followed by 99 cycles at 96 °C (10 s), 50 °C (10 s) and 60 °C (4 min). Amplification took place at 72 °C for 7 min. For the sequencing reaction, the BigDye™ Terminator v3.1 (Applied Biosystems^®^, Foster City, CA, USA) kit was used with the following PCR mixture: 2 μL 5x buffer, 0.5 μL mix BigDye™ Terminator v3.1, 0.2 μL primers, 3.8 μL deionized water and 3.5 μL sample. The final cleaning of the sequencing reaction was performed using ethanol and ethylenediaminetetraacetic acid (EDTA). Results were processed using CodonCode Aligner 9.0.1 (CodonCode Corporation, Centerville, MA, USA).

**Figure 5:**
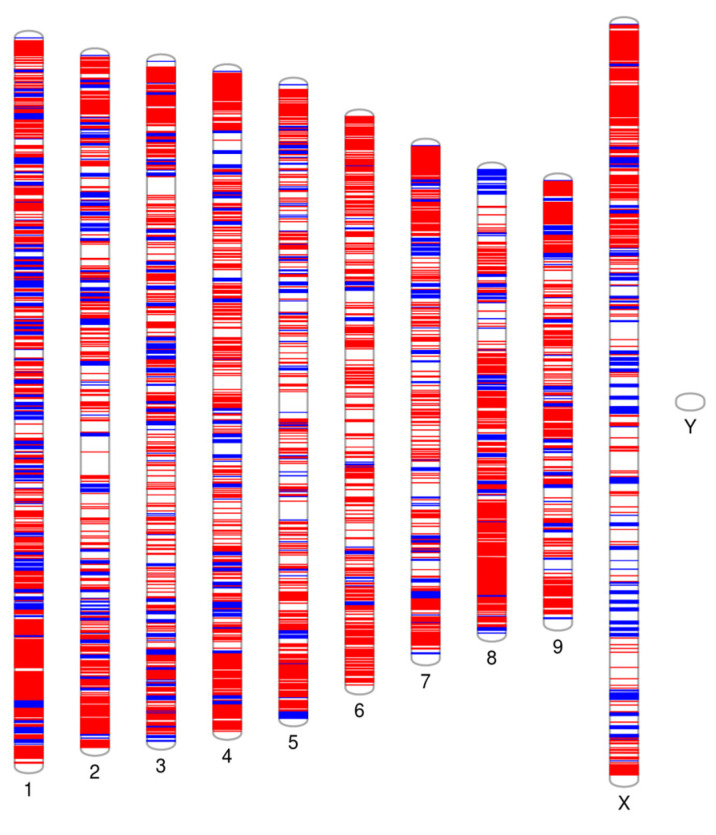
Distribution of selected 4631 SNP positions (blue color) on all 10 *Cannabis* chromosomes (https://visualization.ritchielab.org/phenograms/plot (accessed on 31 August 2023)).

After successful validation of SNP selection, 4631 SNP positions were identified in silico, evenly distributed across the chromosomes. Selection was based on approximately 100,000 bp distance between them. Annotated SNP positions in GFF format were sent to Arbor Biosciences (Daicel) for capture probe design, resulting in successfully designed probes for 4537 SNP positions. Each SNP region was represented by three 80 bp probes—two flanking the SNP and one centered on it. The distribution of SNPs across chromosomes is shown in Figure 5.

### 4.3. NGS Library Preparation

After DNA isolation by Kump and Javornik (1996) [49], NGS libraries were prepared following an in-house protocol. A total of 1000 ng of DNA in a final volume of 100 µL was mixed with Tris-borate EDTA (TBE) buffer and fragmented using an ultrasound bath sonicator for 30 min. Fragmentation success and size distribution were verified by 1% agarose gel electrophoresis. The samples were evaporated to 40 µL in a centrifugal evaporator (vacufuge plus 5305, Eppendorf, Hamburg, Germany).

End-repair of the DNA fragments was performed using the following mixture: 5 µL 10X T4 DNA ligase buffer (New England Biolabs, Ipswitch, MA, USA), 2 µL dNTP (10 mM), 1 µL T4 polynucleotide kinase (10 U/µL) (New England Biolabs, Ipswitch, MA, USA), 0.33 µL T4 polynucleotide polymerase (3 U/µL) (New England Biolabs, Ipswitch, MA, USA), 6.76 µL deionized water and 40 µL of the sonicated DNA sample. The reaction was incubated at 25 °C for 25 min, followed by 12 °C for 10 min and, finally, cooled to 4 °C. The end-repair reaction was cleaned using MagSi-NGS^PREP^ Plus (Magtivio, B.V., Nuth, The Netherlands) magnetic beads at 1.8× reaction volume and 180 µL 70% ethanol.

Samples with magnetic beads were placed on a magnetic separator for 2 min, and the supernatant was discarded. Beads were washed twice with 180 µL of 70% ethanol. Adapter ligation was performed using the following ligation mixture: 20 µL of sample, 3 µL 10X T4 DNA ligase buffer (New England Biolabs, Ipswitch, MA, USA), 0.42 µL P1 adapter, 0.42 µL barcoded adapters (96 combinations) (A1-A96) that were different for each sample, 3 µL dNTP (10 mM), 1 µL T4 DNA ligase (New England Biolabs, USA), 0.66 µL deionized water and 1.5 µL Bst 2.0 WarmStart^®^ DNA polymerase (New England Biolabs, USA). The ligation reaction was incubated in a cyclic thermostat: 22 °C for 30 min, 50 °C for 20 min and then cooled to 4 °C. The ligation reaction was cleaned again with MagSI-NGS^PREP^ beads (Magtivio, B.V., Nuth, The Netherlands) (1.8× reaction volume) and 180 µL 70% ethanol.

NGS library amplification was carried out using the following PCR mixture: 10 µL of libraries, 10 µL 5X KAPA HiFi Fidelity buffer (Roche, Basel, Switzerland), 1.5 µL dNTP (10 mM), 25 µL P1amp primer, 2.5 µL T_PCR_A primer, 22.5 µL deionized water and 2 µL of HotStart DNA polymerase (Roche, Basel, Switzerland). Amplification was carried out according to the following procedure: initial denaturation at 95 °C for 3 min, followed by 8 cycles of 98 °C for 20 s, 60 °C for 30 s and 72 °C for 30 s. A final extension was performed at 72 °C for 1 min. The reaction was cooled to 4 °C.

Amplified libraries were cleaned using MagSI-NGS^PREP^ beads (Magtivio, B.V., Nuth, The Netherlands) (0.8× reaction volume) and 180 µL 85% ethanol. Libraries were resuspended in 20 µL TE buffer and quantified using the Agilent 2100 DNA Bioanalyzer system with the DNA 1000 kit (Agilent, Santa Clara, CA, USA) for quality control. Library concentrations were quantified using qPCR QuantStudio^™^ 5 (ThermoFisher Scientific, Waltham, MA, USA) with the KAPA Library Quantification Kit for Ion Torrent™ Platforms (Kapa Biosystems, Wilmington, MA, USA), following the manufacturer’s instructions. After quantifications, libraries were normalized to equimolar concentrations and pooled into four reactions, each containing 2000 ng of total NGS library. Barcodes with adapters and primers used in the NGS library preparation are detailed in Supplementary Tables S1 and S2.

### 4.4. Hybridization Capture-Based Target Enrichment

A hybridization mix with biotinylated probes was prepared and incubated at 60 °C for 10 min. Aliquots of 18.5 μL were distributed in 0.2 mL PCR strip tubes for each of the four capture reactions. Separately, 5 μL of blockers was aliquoted into 0.2 mL PCR strip tubes for each capture reaction. To each tube containing blockers, 7 µL of pooled library was added and gently mixed by pipetting. The tubes were then placed in a thermal cycler and incubated at 95 °C for 5 min. The hybridization mix was then added into a thermal cycler for five minutes. After this denaturation step, the tubes were removed, and 18 μL of the hybridization mix was added into each tube containing the blockers mix. The reactions were incubated for 16 h at 65 °C to allow hybridization.

The following day, streptavidin magnetic beads were prepared by transferring 120 μL of beads into each of four 0.2 mL PCR strip tubes. The tubes were put on a magnetic particle collector until the suspension cleared. After 2 min, the supernatant was discarded, and 800 μL of binding buffer was added. Samples were placed back on a magnetic particle collector, and after 2 min, supernatant was removed. Washing steps were repeated two more times. The washed streptavidin magnetic beads were resuspended in 280 μL of binding buffer, and 70 μL aliquots were distributed into four 0.2 mL PCR strip tubes. The reactions were heated at 65 °C for 2 min, and 30 μL of each hybridization reaction was transferred to the corresponding heated bead aliquots. The capture reactions were put on a magnetic particle collector, and the supernatant was discarded. A total of 180 μL of Wash Buffer X (Arbor Bioscience, Ann Arbor, MI, USA) was added, and reactions were incubated at 65 °C for 5 min. The wash step was repeated three more times for a total of four washes. Following the final wash, the enriched libraries were resuspended in 30 μL of Buffer E (Arbor Bioscience, Ann Arbor, MI, USA) and amplified using KAPA HiFi HotStart ReadyMix (Roche, Basel, Switzerland). PCR amplification was performed with the following cycling conditions: initial denaturation at 98 °C for 2 min, followed by 10 cycles of 98 °C for 20 s, 60 °C for 30 s and 72 °C 30 s. A final extension was performed at 75 °C for 5 min and cooled to 8 °C. The enriched and amplified libraries were then purified using MagSI-NGS^PREP^ Plus beads (Magtivio, B.V., Nuth, The Nederlands) and resuspended in 20 μL TE buffer. Captured libraries were quantified using qPCR.

### 4.5. Sequencing

The four captured reactions were pooled into a single reaction with a final concentration of 100 pM. Emulsion PCR was performed using an Ion OneTouch^TM^ 2 system (Thermo Fisher Scientific, Waltham, MA, USA) with the Ion PI^TM^ Ion Sphere Particles (ISP) (Thermo Fisher Scientific, Waltham, MA, USA) and the corresponding solution. Template-positive ISPs (Thermo Fisher Scientific, Waltham, MA, USA) were then washed using Resuspension Solution and enriched using an Ion OneTouch^TM^ ES system (Thermo Fisher Scientific, Waltham, MA, USA) and Dynabeads^TM^ MyOne^TM^ Streptavidin C1 beads (Thermo Fisher Scientific, Waltham, MA, USA). The instrument run time for enrichment was 37 min. After enrichment, the supernatant was removed. For sequencing, an Ion PI^TM^ Hi-Q^TM^ Sequencing 200 Kit (Thermo Fisher Scientific, Waltham, MA, USA) was used, following the manufacturer’s instructions. Nuclease-free water was added to the pellet, which was then resuspended. Ion PI^TM^ Controle Ion Sphere^TM^ Particles (Thermo Fisher Scientific, Waltham, MA, USA) and a sequencing primer were added to the enriched ISPs. Sequencing was performed on an Ion Proton^TM^ Sequencer using an Ion PI^TM^ v3 Chip (Thermo Fisher Scientific, Waltham, MA, USA). A total of 171 samples were sequenced across two chips: a total of 96 samples were loaded on the first chip, and 75 samples were loaded on the second. The sequencing data were delivered adapter-trimmed in UBAM format.

### 4.6. Analysis of Sequencing Data

Raw reads (BioProject accession number: PRJNA1311468, https://www.ncbi.nlm.nih.gov/sra/PRJNA1311468 (accessed on 27 August 2025)) in UBAM format were imported into CLC Genomics Workbench version 22 (Qiagen, Venlo, The Nederlands). Reads were trimmed. Short reads were removed using the ‘Trim Reads’ tool in CLC. For quality control (QC), the ‘QC for sequencing reads’ tool in CLC was applied. Each set of trimmed reads was mapped to the *Cannabis sativa* L. reference genome (cs10) using the ‘Map Reads to Reference’ tool in CLC. The resulting alignments were exported as BAM files.

SNP calling was performed using the Genome Analysis Toolkit (GATK) (https://gatk.broadinstitute.org/hc/en-us (accessed on 5 February 2023)), following the GATK Best Practices Workflows (https://gatk.broadinstitute.org/hc/en-us/sections/360007226651-Best-Practices-Workflows (accessed on 5 February 2025)). The procedure included the following steps: (1) creating a sequence dictionary file from a reference sequence using the ‘CreateSequenceDictionary’ tool, (2) identification of duplicates using the ‘MarkDuplicates’ tool, (3) sorting of BAM files using the ‘SortSam’ tool, (4) recalibration of qualitative values using the ‘BseRecalibrator’ tool according to known SNP positions and the ‘ApplyBQSR.bash’ tool, (5) determination of SNP positions using the ‘HaplotypeCaller’ tool and (6) combining individual VCF files to one VCF file using the ‘CombineGVCFs’ tool. Genotyping of samples followed using the ‘GenotypeGVCFs’ tool, which is part of a GATK tool, where genotyping of samples occurred according to 4537 analyzed SNP positions (Supplementary File S1 and Supplementary Data S1). Data for monomorphic positions were kept using the ‘Include-non-variant-sites’ command. Data were filtered according to the quality and depth of reads using the ‘VariantFiltration’ tool, where the depth threshold was <5 and quality threshold was >20. The final combined VCF file was used for subsequent bioinformatic analyses (Supplementary Data S2).

### 4.7. Population Structure

Population structure analysis was performed based on the SNP data for each genotype. The VCF file was first converted into PLINK file format. Analysis of population structure was conducted using Admixture version 1.3.0 (https://dalexander.github.io/admixture/download.html (accessed on 31 August 2023)) [50], where models assuming K = 1 to 20 genetic clusters were tested. For each K value, the cross-validation error (CV) was calculated, and the optimal number of populations was determined as the K with the lowest CV value. Visualization of the Admixture results was carried out using the StructuRly application version 0.1.0 (https://github.com/nicocriscuolo/StructuRly (accessed on 31 August 2023)) [51].

### 4.8. Genome-Wide Association Studies (GWAS)

Genome-wide association studies (GWAS) were conducted using the genotyped VCF file, which was imported into Tassel version 5.2.88 [52]. Genotype data from 171 hemp-type genotypes were integrated with phenotypic data for traits collected from a subset of 55 individuals. Missing genotype data were imputed using the ‘LD KNNi Imputation’ method, with linkage disequilibrium (LD) set to 30, K-nearest neighbor set to 10 and a maximum LD search distance of 10 million bp. Quality control of SNP data involved filtering out SNPs with minor allele frequency (MAF) <0.05 and maximum heterozygosity >1 and the removal of minor SNP statistics and indels. After filtering, 3670 high-quality SNPs remained from the initial 4537 (Supplementary Data S3 and S4).

Kinship matrix was computed using the centered identity-by-state (IBS) method to account for population structure and genetic relatedness. Principal component analysis (PCA) was performed in Tassel 5.88 software using five components (Supplementary Data S5) based on the filtered VCF file (Supplementary Data S4). GWAS was conducted using a mixed linear model (MLM) in Tassel 5.88 software, incorporating both the PCA and kinship matrix, resulting in raw *p*-values. The MLM model is based on principal components and kinship matrix to reduce false positives, which can be related to the family population structure and relatedness (Supplementary Data S6). Marker effects on trait variance (R^2^) were assessed to determine the proportion of the phenotypic variation explained by each SNP. For multiple testing corrections, a false discovery rate (FDR) approach was applied using the ‘qvalue’ package in R Studio software version 4.2.3. at 0.01, 0.001 and 0.0001 [53] instead of a Bonferroni correction, which was considered too conservative. Manhattan plots were generated using the ‘qqman’ package [54], and Q-Q plots were produced to assess the distribution of *p*-values.

Based on the generated Manhattan plots, we identified genes associated with our SNPs according to the significant loci. We loaded the annotated cs10 genome into CLC Genomic Workbench 23.0.3 and searched for genes at the significant SNP positions. The annotated genes were then queried in the UniProt database, the NCBI database, the KEGG database and the Gene Ontology database, where we obtained information about each gene’s function and the biological processes in which it is involved. For genes that could not be found using CLC, we searched for them in the NCBI database by retrieving sequences of 2001 bp in length (1000 bp upstream and 1000 bp downstream of the SNP position) and using the BLAST tool (Basic Local Alignment Search Tool) to identify highly similar alignments. This allowed us to obtain information about the genes in which our SNPs are located.

## 5. Conclusions

This study investigated the population structure and genotype–phenotype relationships of hemp-type phenotypes across three varieties: Carmagnola selected (CS), Tiborszallasi (TS) and Finola selection (FS). Clear genetic differentiation was detected among the varieties. TS and FS displayed pronounced internal substructure, each forming two genetic clusters, whereas CS showed no detectable substructure, indicating a comparatively higher level of genetic homogeneity. Additionally, FS was genetically distinct from CS and TS, highlighting substantial divergence among the analyzed varieties.

Genome-wide association analysis integrating 45 phenotypic traits with 3670 SNP markers enabled the identification of significant loci and candidate genes associated with all evaluated traits. Although most identified genes were not directly linked to known cannabinoid or terpene biosynthetic pathways, a berberine-bridge enzyme-like 15 gene was associated with the CBD-A trait and showed correlation with both CBD-A and THC-A.

To further improve the resolution of gene–trait associations, future studies should investigate broader genomic regions surrounding significant SNPs, which may capture regulatory elements or biosynthetic genes more directly involved in phenotypic expression. Such approaches will enhance the application of GWAS results in marker-assisted selection and support functional validation of candidate genes for hemp improvement.

The methodology for selecting SNPs that are well distributed across chromosomes serves as a robust indicator of genetic diversity. This represents proof of principle, demonstrating that the sequences are spaced appropriately for the development of a commercial genotyping chip. This study offers valuable insight into hemp genetics, homogeneity and the comparison between different phenotypes. To obtain more accurate results, research should be expanded to include a greater number of unique hemp-type and drug-type varieties.

## Figures and Tables

**Table 1:** Description of selected phenotypes according to visual traits.

Phenotype	Height	Color	Leaf Size	Inflorescence	Anthocyanin Coloration of Leaf Petiole	Branching
CI	Tall	Bright	Large	Small	No	***
CII	Tall	Dark	Small	Small	Yes	*****
TI	Tall	Medium	Medium	Small	No	*****
TII	Medium	Dark	Medium	Medium	Yes	***
TIII	Small	Dark	Small	Medium	Yes	**
TIV	Medium	Dark	Large	Big	No	***
TV	Small	Medium	Small	Medium	Yes	*
FI	Tall	Dark	Medium	Big	No	*****
FII	Medium	Medium	Medium	Big	No	***
FII	Medium	Bright	Medium	Medium	Yes	*****
FIV	Medium	Dark	Medium	Big	Yes	*****

CI–CII—‘Carmagnola selected’, TI–TV—‘Tiborszallasi’, FI–FIV—‘Finola’ selection, *—very sparsely branched, **—low branched, ***—moderately branched, *****—very strongly branched.

**Table 2:** Intersection and distribution of polymorphisms across *Cannabis sativa* L. chromosomes for all three varieties (‘Finola’, ‘Santhica 27’ and ‘Purple Kush’).

Intersection	Chromosome 1 (101,209 kb)	Chromosome 2 (96,347 kb)	Chromosome 3 (94,671 kb)	Chromosome 4 (91,941 kb)	Chromosome 5 (88,182 kb)	Chromosome 6 (79,355 kb)	Chromosome 7 (71,238 kb)	Chromosome8 (64,622 kb)	Chromosome 9 (61,561 kb)	Chromosome X (104,666 kb)
F, S27, PK	176	75	83	84	72	113	67	116	85	89
F, S27	327	175	216	262	174	195	155	253	213	251
F, PK	635	394	333	357	307	345	292	416	293	286
S27, PK	361	184	221	218	171	253	144	275	217	171
Total	1499	828	853	921	724	906	658	1060	808	797
Distribution	67.5 kb/SNP	116.4 kb/SNP	111.9 kb/SNP	99.8 kb/SNP	121.8 kb/SNP	87.6 kb/SNP	108.4 kb/SNP	61.0 kb/SNP	76.2 kb/SNP	131.3 kb/SNP

F—‘Finola’, S27—‘Santhica 27’, PK—‘Purple Kush’.

**Table 3:** Selected primers with positions on chromosomes and sequences.

Primers	Chromosome	Position on Chromosome	Primer sequences 5′-3′	
SNP 1	1	443869	L: CCGTAGAAGGTGGCAAATGT R: TGCTTGTTTTCTTGGTTTTAGG	Common positions to ‘Finola’, ‘Santhica 27’ and ‘Purple Kush’
SNP 2	2	7186803	L: AGCCATTCCAAAAGCATTCC R: TTACAGCTTGTGCCAGCAAT	
SNP 3	3	1187767	L: TAGGAATTGAACCGGATTGC R: TCAGCCTGCAATAATCGAAA	
SNP 4	4	26919313	L: TTTTAGCGGGAACAACAACC R: AAGGAGGGAATTGGAAGAGC	
SNP 5	4	66059933	L: AACTTGCAGCTCAAGGGAAA R: AAATCCACCATGGAAGGACA	
SNP 6	6	78288161	L: GAAAACAGGTGTGGGAAGGA R: CCCGTTTGCAACATTTCTCT	
SNP 7	9	3445493	L: TTCGTTTTGATGTATGCACTCC R: TGCATGCTTAGACCCATCTG	
SNP 8	10	55429963	L: TTCAGCACACCACGACATAA R: AGGGTTGGGTGAATGAATGA	
SNP 9	1	5266236	L: TCTCCTTGATCAGCAACCAA R: TGCTCTCCTCCCTCAACAGT	Common positions to ‘Finola’ and ‘Santhica 27’
SNP 10	1	1037237	L: AAGCTTCACCTTCTGCGAAA R: CAAATGCCGGAGTTTGACTT	
SNP 11	3	7382936	L: TTTCCCCGATCTTAGGGTTT R: TGGGAAAGTGAGGAGACTGG	
SNP 12	4	69568690	L: TTCGCTGAAAACGACAAATG R: CCCGTCTAATCGGAAATTGA	
SNP 13	6	70711381	L: ACTGCCTTCGTTTTCACCAG R: ATTCAGGGCCATGTCAAAAG	
SNP 14	8	42763723	L: GCACAAGAACTAATGGGCTGT R: ATATGGTGTTGGTGGCGTTT	

## Data Availability

The datasets generated and/or analyzed during the current study are available in the Sequence Read Archive (SRA), https://www.ncbi.nlm.nih.gov/sra/PRJNA1311468 (accessed on 26 December 2025), BioProject accession number: PRJNA1311468, and in FigShare repository at 10.6084/m9.figshare.30762896 (Supplementary Data S1), https://doi.org/10.6084/m9.figshare.23761965 (Supplementary Data S2), https://doi.org/10.6084/m9.figshare.23763873 (Supplementary Data S3) https://doi.org/10.6084/m9.figshare.23764314 (Supplementary Data S4), https://doi.org/10.6084/m9.figshare.23764482 (Supplementary Data S5), https://doi.org/10.6084/m9.figshare.23765337 (Supplementary Data S6).

## Supplementary Materials

The following supporting information can be downloaded at: https://www.mdpi.com/article/10.3390/plants15020202/s1. Table S1: Barcode sequences with adapters used in NGS library preparation. Adapters are in *Italics*, Table S2: Primers with sequences used in NGS library amplification, Collection of Images S1: Photos of individual phenotypes within variety ‘Carmagnola selected’, ‘Tiborszallasi’ and ‘Finola selection’ based on visual traits, File S1: SNP calling, Table S3: Annotated probable genes. Figure S1: Manhattan plots showing significant SNP positions above the FDR threshold across the 10 *Cannabis* chromosomes and Q-Q plot for 3-carene, Figure S2: Manhattan plots showing significant SNP positions (red dots) above the FDR threshold across the 10 *Cannabis* chromosomes and Q-Q plot for α-bisabolol, Figure S3: Manhattan plots showing significant SNP positions (red dots) above the FDR threshold across the 10 *Cannabis* chromosomes and Q-Q plot for α-cedrene, Figure S4: Manhattan plots showing significant SNP positions (red dots) above the FDR threshold across the 10 *Cannabis* chromosomes and Q-Q plot for α-humulene, Figure S5: Manhattan plots showing significant SNP positions (red dots) above the FDR threshold across the 10 *Cannabis* chromosomes and Q-Q plot for anthocyanin coloration of leaf petiole, Figure S6: Manhattan plots showing significant SNP positions (red dots) above the FDR threshold across the 10 *Cannabis* chromosomes and Q-Q plot for α- pinene, Figure S7: Manhattan plots showing significant SNP positions (red dots) above the FDR threshold across the 10 *Cannabis* chromosomes and Q-Q plot for α-terpinen, Figure S8: Manhattan plots showing significant SNP positions (red dots) above the FDR threshold across the 10 *Cannabis* chromosomes and Q-Q plot for α-terpineol, Figure S9: Manhattan plots showing significant SNP positions (red dots) above the FDR threshold across the 10 *Cannabis* chromosomes and Q-Q plot for β-caryophyllene, Figure S10: Manhattan plots showing significant SNP positions (red dots) above the FDR threshold across the 10 *Cannabis* chromosomes and Q-Q plot for β-citronellol, Figure S11: Manhattan plots showing significant SNP positions (red dots) above the FDR threshold across the 10 *Cannabis* chromosomes and Q-Q plot forβ-eudesmol, Figure S12: Manhattan plots showing significant SNP positions (red dots) above the FDR threshold across the 10 *Cannabis* chromosomes and Q-Q plot for borneol, Figure S13: Manhattan plots showing significant SNP positions (red dots) above the FDR threshold across the 10 *Cannabis* chromosomes and Q-Q plot for β-pinene, Figure S14: Manhattan plots showing significant SNP positions (red dots) above the FDR threshold across the 10 *Cannabis* chromosomes and Q-Q plot for branching, Figure S15: Manhattan plots showing significant SNP positions (red dots) above the FDR threshold across the 10 *Cannabis* chromosomes and Q-Q plot for camphene, Figure S16: Manhattan plots showing significant SNP positions (red dots) above the FDR threshold across the 10 *Cannabis* chromosomes and Q-Q plot for camphor, Figure S17: Manhattan plots showing significant SNP positions (red dots) above the FDR threshold across the 10 *Cannabis* chromosomes and Q-Q plot for caryophyllene oxide, Figure S18: Manhattan plots showing significant SNP positions (red dots) above the FDR threshold across the 10 *Cannabis* chromosomes and Q-Q plot for CBD, Figure S19: Manhattan plots showing significant SNP positions (red dots) above the FDR threshold across the 10 *Cannabis* chromosomes and Q-Q plot for CBC-A, Figure S20: Manhattan plots showing significant SNP positions (red dots) above the FDR threshold across the 10 *Cannabis* chromosomes and Q-Q plot for CBD, Figure S21: Manhattan plots showing significant SNP positions (red dots) above the FDR threshold across the 10 *Cannabis* chromosomes and Q-Q plot for CBD-A, Figure S22: Manhattan plots showing significant SNP positions (red dots) above the FDR threshold across the 10 *Cannabis* chromosomes and Q-Q plot for CBG-A, Figure S23: Manhattan plots showing significant SNP positions (red dots) above the FDR threshold across the 10 *Cannabis* chromosomes and Q-Q plot for CBN, Figure S24: Manhattan plots showing significant SNP positions (red dots) above the FDR threshold across the 10 *Cannabis* chromosomes and Q-Q plot for cis-nerolidol, Figure S25: Manhattan plots showing significant SNP positions (red dots) above the FDR threshold across the 10 *Cannabis* chromosomes and Q-Q plot for color, Figure S26: Manhattan plots showing significant SNP positions (red dots) above the FDR threshold across the 10 *Cannabis* chromosomes and Q-Q plot for delta-8-THC, Figure S27: Manhattan plots showing significant SNP positions (red dots) above the FDR threshold across the 10 *Cannabis* chromosomes and Q-Q plot for delta-9-THC-A, Figure S28: Manhattan plots showing significant SNP positions (red dots) above the FDR threshold across the 10 *Cannabis* chromosomes and Q-Q plot for fenchone, Figure S29: Manhattan plots showing significant SNP positions (red dots) above the FDR threshold across the 10 *Cannabis* chromosomes and Q-Q plot for geranyl acetate, Figure S30: Manhattan plots showing significant SNP positions (red dots) above the FDR threshold across the 10 *Cannabis* chromosomes and Q-Q plot for geranyl isobutirate, Figure S31: Manhattan plots showing significant SNP positions (red dots) above the FDR threshold across the 10 *Cannabis* chromosomes and Q-Q plot for γ-terpinene, Figure S32: Manhattan plots showing significant SNP positions (red dots) above the FDR threshold across the 10 *Cannabis* chromosomes and Q-Q plot for inflorescence, Figure S33: Manhattan plots showing significant SNP positions (red dots) above the FDR threshold across the 10 *Cannabis* chromosomes and Q-Q plot for isoborneol, Figure S34: Manhattan plots showing significant SNP positions (red dots) above the FDR threshold across the 10 *Cannabis* chromosomes and Q-Q plot for leaf size, Figure S35: Manhattan plots showing significant SNP positions (red dots) above the FDR threshold across the 10 *Cannabis* chromosomes and Q-Q plot for limonene, Figure S36: Manhattan plots showing significant SNP positions (red dots) above the FDR threshold across the 10 *Cannabis* chromosomes and Q-Q plot for linalool, Figure S37: Manhattan plots showing significant SNP positions (red dots) above the FDR threshold across the 10 *Cannabis* chromosomes and Q-Q plot for menthol, Figure S38: Manhattan plots showing significant SNP positions (red dots) above the FDR threshold across the 10 *Cannabis* chromosomes and Q-Q plot for neryl acetate, Figure S39: Manhattan plots showing significant SNP positions (red dots) above the FDR threshold across the 10 *Cannabis* chromosomes and Q-Q plot for p-cimene, Figure S40: Manhattan plots showing significant SNP positions (red dots) above the FDR threshold across the 10 *Cannabis* chromosomes and Q-Q plot for phytol, Figure S41: Manhattan plots showing significant SNP positions (red dots) above the FDR threshold across the 10 *Cannabis* chromosomes and Q-Q plot for size of the plant, Figure S42: Manhattan plots showing significant SNP positions (red dots) above the FDR threshold across the 10 *Cannabis* chromosomes and Q-Q plot for terpineolene.

## Abbreviations

The following abbreviations are used in this manuscript:

CBC	Cannabichromene
CBC-A	Cannabichromenic acid
CBD	Cannabidiol
CBD-A	Cannabidiolic acid
CBG	Cannabigerol
CBG-A	Cannabigerolic acid
CS	Carmagnola selected
delta-9-THC	Delta-9-tetrahydrocannabinol
DNA	Deoxyribonucleic acid
FDR	False discovery rate
FS	Finola selection
GPP	Geranyl diphosphate
NGS	Next-generation sequencing
OLA	Olivetolic acid
PCR	Polymerase chain reaction
SNP	Single-nucleotide position
SRA	Sequencing Read Archive
THC-A	Tertrahydrocannabinolic acid
TS	Tiborszallasi
