# Supplementary material for: Population Structure of Genotypes and Genome-Wide Association Studies of Cannabinoids and Terpenes Synthesis in Hemp (Cannabis sativa L.)
Source: Plants (Basel). 2026 Jan 8;15(2):202. doi: 10.3390/plants15020202 (PMC12845109; doi:10.3390/plants15020202)
Supplement: Supplementary file 1 [file plants-15-00202-s001.zip › Supplementary-information.pdf]

SUPPLEMENTARY INFORMATION

Population Structure of Genotypes and Genome-Wide Association Studies of Cannabinoids and Terpenes Synthesis in Hemp (*Cannabis sativa* L.) Plants

Marjeta Eržen[1,2,*], Andreja Čerenak[1], Tjaša Cesar[2], Jernej Jakše[2]

[1]Department for Plants, Soil and the Environment, Slovenian Institute of Hop Research and Brewing, Cesta Žalskega tabora 2, 3310 Žalec, Slovenia

2Department for Agronomy, Biotechnical Faculty, University of Ljubljana, Jamnikarjeva 101, 1000 Ljubljana, Slovenia

*** Correspondence:**
Corresponding Author: Marjeta Eržen, Biotechnical faculty, University of Ljubljana, Jamnikarjeva 101, 1000 Ljubljana, Slovenia

Table S1: Barcode sequences with adapters used in NGS library preparation. Adapters are in *Italics*.

Barcode	Barcode sequence with adapters 5'-3'
**A _Barcode-1**	CCATCTCATCCCTGCGTGTCTCCGACTCAG*CTAAGGTAAC*GAT
**A _Barcode-2**	CCATCTCATCCCTGCGTGTCTCCGACTCAG*TAAGGAGAAC*GAT
**A _Barcode-3**	CCATCTCATCCCTGCGTGTCTCCGACTCAG*AAGAGGATTC*GAT
**A _Barcode-4**	CCATCTCATCCCTGCGTGTCTCCGACTCAG*TACCAAGATC*GAT
**A _Barcode-5**	CCATCTCATCCCTGCGTGTCTCCGACTCAG*CAGAAGGAAC*GAT
**A _Barcode-6**	CCATCTCATCCCTGCGTGTCTCCGACTCAG*CTGCAAGTTC*GAT
**A _Barcode-7**	CCATCTCATCCCTGCGTGTCTCCGACTCAG*TTCGTGATTC*GAT
**A _Barcode-8**	CCATCTCATCCCTGCGTGTCTCCGACTCAG*TTCCGATAAC*GAT
**A _Barcode-9**	CCATCTCATCCCTGCGTGTCTCCGACTCAG*TGAGCGGAAC*GAT
**A _Barcode-10**	CCATCTCATCCCTGCGTGTCTCCGACTCAG*CTGACCGAAC*GAT
**A _Barcode-11**	CCATCTCATCCCTGCGTGTCTCCGACTCAG*TCCTCGAATC*GAT
**A _Barcode-12**	CCATCTCATCCCTGCGTGTCTCCGACTCAG*TAGGTGGTTC*GAT
**A _Barcode-13**	CCATCTCATCCCTGCGTGTCTCCGACTCAG*TCTAACGGAC*GAT
**A _Barcode-14**	CCATCTCATCCCTGCGTGTCTCCGACTCAG *TTGGAGTGTC*GAT
**A _Barcode-15**	CCATCTCATCCCTGCGTGTCTCCGACTCAG *TCTAGAGGTC*GAT
**A _Barcode-16**	CCATCTCATCCCTGCGTGTCTCCGACTCAG *TCTGGATGAC*GAT
**A _Barcode-17**	CCATCTCATCCCTGCGTGTCTCCGACTCAG *TCTATTCGTC*GAT
**A _Barcode-18**	CCATCTCATCCCTGCGTGTCTCCGACTCAG *AGGCAATTGC*GAT
**A _Barcode-19**	CCATCTCATCCCTGCGTGTCTCCGACTCAG *TTAGTCGGA*CGAT
**A _Barcode-20**	CCATCTCATCCCTGCGTGTCTCCGACTCAG *CAGATCCATC*GAT
**A _Barcode-21**	CCATCTCATCCCTGCGTGTCTCCGACTCAG *TCGCAATTAC*GAT
**A _Barcode-22**	CCATCTCATCCCTGCGTGTCTCCGACTCAG *TTCGAGACGC*GAT
**A _Barcode-23**	CCATCTCATCCCTGCGTGTCTCCGACTCAG *TGCCACGAAC*GAT
**A _Barcode-24**	CCATCTCATCCCTGCGTGTCTCCGACTCAG *AACCTCATTC*GAT
**A _Barcode-25**	CCATCTCATCCCTGCGTGTCTCCGACTCAG*CCTGAGATAC*GAT

**A _Barcode-26**	CCATCTCATCCCTGCGTGTCTCCGACTCAG*TTACAACCTC*GAT
**A _Barcode-27**	CCATCTCATCCCTGCGTGTCTCCGACTCAG*AACCATCCGC*GAT
**A _Barcode-28**	CCATCTCATCCCTGCGTGTCTCCGACTCAG*ATCCGGAATC*GAT
**A _Barcode-29**	CCATCTCATCCCTGCGTGTCTCCGACTCAG*TCGACCACTC*GAT
**A _Barcode-30**	CCATCTCATCCCTGCGTGTCTCCGACTCAG*CGAGGTTATC*GAT
**A _Barcode-31**	CCATCTCATCCCTGCGTGTCTCCGACTCAG*TCCAAGCTGC*GAT
**A _Barcode-32**	CCATCTCATCCCTGCGTGTCTCCGACTCAG*TCTTACACAC*GAT
**A _Barcode-33**	CCATCTCATCCCTGCGTGTCTCCGACTCAG *TTCTCATTGAAC*GAT
**A _Barcode-34**	CCATCTCATCCCTGCGTGTCTCCGACTCAG *TCGCATCGTTC*GAT
**A _Barcode-35**	CCATCTCATCCCTGCGTGTCTCCGACTCAG *TAAGCCATTGTC*GAT
**A _Barcode-36**	CCATCTCATCCCTGCGTGTCTCCGACTCAG *AAGGAATCGTC*GAT
**A _Barcode-37**	CCATCTCATCCCTGCGTGTCTCCGACTCAG *CTTGAGAATGTC*GAT
**A _Barcode-38**	CCATCTCATCCCTGCGTGTCTCCGACTCAG *TGGAGGACGGAC*GAT
**A _Barcode-39**	CCATCTCATCCCTGCGTGTCTCCGACTCAG *TAACAATCGGC*GAT
**A _Barcode-40**	CCATCTCATCCCTGCGTGTCTCCGACTCAG *CTGACATAATC*GAT
**A _Barcode-41**	CCATCTCATCCCTGCGTGTCTCCGACTCAG *TTCCACTTCGC*GAT
**A _Barcode-42**	CCATCTCATCCCTGCGTGTCTCCGACTCAG *AGCACGAATC*GAT
**A _Barcode-43**	CCATCTCATCCCTGCGTGTCTCCGACTCAG *CTTGACACCGC*GAT
**A _Barcode-44**	CCATCTCATCCCTGCGTGTCTCCGACTCAG *TTGGAGGCCAGC*GAT
**A _Barcode-45**	CCATCTCATCCCTGCGTGTCTCCGACTCAG *TGGAGCTTCCTC*GAT
**A _Barcode-46**	CCATCTCATCCCTGCGTGTCTCCGACTCAG *TCAGTCCGAAC*GAT
**A _Barcode-47**	CCATCTCATCCCTGCGTGTCTCCGACTCAG *TAAGGCAACCAC*GAT
**A _Barcode-48**	CCATCTCATCCCTGCGTGTCTCCGACTCAG *TTCTAAGAGAC*GAT
**A _Barcode-49**	CCATCTCATCCCTGCGTGTCTCCGACTCAG*TCCTAACATAAC*GAT
**A _Barcode-50**	CCATCTCATCCCTGCGTGTCTCCGACTCAG*CGGACAATGGC*GAT
**A _Barcode-51**	CCATCTCATCCCTGCGTGTCTCCGACTCAG*TTGAGCCTATTC*GAT
**A _Barcode-52**	CCATCTCATCCCTGCGTGTCTCCGACTCAG*CCGCATGGAAC*GAT
**A _Barcode-53**	CCATCTCATCCCTGCGTGTCTCCGACTCAG*CTGGCAATCCTC*GAT
**A _Barcode-54**	CCATCTCATCCCTGCGTGTCTCCGACTCAG*CCGGAGAATCGC*GAT
**A _Barcode-55**	CCATCTCATCCCTGCGTGTCTCCGACTCAG*TCCACCTCCTC*GAT
**A _Barcode-56**	CCATCTCATCCCTGCGTGTCTCCGACTCAG*CAGCATTAATTC*GAT
**A _Barcode-57**	CCATCTCATCCCTGCGTGTCTCCGACTCAG*TCTGGCAACGGC*GAT
**A _Barcode-58**	CCATCTCATCCCTGCGTGTCTCCGACTCAG*TCCTAGAACAC*GAT
**A _Barcode-59**	CCATCTCATCCCTGCGTGTCTCCGACTCAG*TCCTTGATGTTC*GAT
**A _Barcode-60**	CCATCTCATCCCTGCGTGTCTCCGACTCAG*TCTAGCTCTTC*GAT
**A _Barcode-61**	CCATCTCATCCCTGCGTGTCTCCGACTCAG*TCACTCGGATC*GAT
**A _Barcode-62**	CCATCTCATCCCTGCGTGTCTCCGACTCAG*TTCCTGCTTCAC*GAT
**A _Barcode-63**	CCATCTCATCCCTGCGTGTCTCCGACTCAG *CCTTAGAGTTC*GAT
**A _Barcode-64**	CCATCTCATCCCTGCGTGTCTCCGACTCAG*CTGAGTTCCGAC*GAT
**A _Barcode-65**	CCATCTCATCCCTGCGTGTCTCCGACTCAG *TCCTGGCACATC*GAT
**A _Barcode-66**	CCATCTCATCCCTGCGTGTCTCCGACTCAG *CCGCAATCATC*GAT
**A _Barcode-67**	CCATCTCATCCCTGCGTGTCTCCGACTCAG *TTCCTACCAGTC*GAT
**A _Barcode-68**	CCATCTCATCCCTGCGTGTCTCCGACTCAG *TCAAGAAGTTC*GAT
**A _Barcode-69**	CCATCTCATCCCTGCGTGTCTCCGACTCAG *TTCAATTGGC*GAT
**A _Barcode-70**	CCATCTCATCCCTGCGTGTCTCCGACTCAG *CCTACTGGTC*GAT
**A _Barcode-71**	CCATCTCATCCCTGCGTGTCTCCGACTCAG *TGAGGCTCCGAC*GAT

**A _Barcode-72**	CCATCTCATCCCTGCGTGTCTCCGACTCAG *CGAAGGCCACAC*GAT
**A _Barcode-73**	CCATCTCATCCCTGCGTGTCTCCGACTCAG*TCTGCCTGTC*GAT
**A _Barcode-74**	CCATCTCATCCCTGCGTGTCTCCGACTCAG*CGATCGGTTC*GAT
**A _Barcode-75**	CCATCTCATCCCTGCGTGTCTCCGACTCAG*TCAGGAATAC*GAT
**A _Barcode-76**	CCATCTCATCCCTGCGTGTCTCCGACTCAG*CGGAAGAACCTC*GAT
**A _Barcode-77**	CCATCTCATCCCTGCGTGTCTCCGACTCAG*CGAAGCGATTC*GAT
**A _Barcode-78**	CCATCTCATCCCTGCGTGTCTCCGACTCAG*CAGCCAATTCTC*GAT
**A _Barcode-79**	CCATCTCATCCCTGCGTGTCTCCGACTCAG*CCTGGTTGTC*GAT
**A _Barcode-80**	CCATCTCATCCCTGCGTGTCTCCGACTCAG*TCGAAGGCAGGC*GAT
**A _Barcode-81**	CCATCTCATCCCTGCGTGTCTCCGACTCAG *CCTGCCATTCGC*GAT
**A _Barcode-82**	CCATCTCATCCCTGCGTGTCTCCGACTCAG *TTGGCATCTC*GAT
**A _Barcode-83**	CCATCTCATCCCTGCGTGTCTCCGACTCAG *CTAGGACATTC*GAT
**A _Barcode-84**	CCATCTCATCCCTGCGTGTCTCCGACTCAG*CTTCCATAAC*GAT
**A _Barcode-85**	CCATCTCATCCCTGCGTGTCTCCGACTCAG*CCAGCCTCAAC*GAT
**A _Barcode-86**	CCATCTCATCCCTGCGTGTCTCCGACTCAG*CTTGGTTATTC*GAT
**A _Barcode-87**	CCATCTCATCCCTGCGTGTCTCCGACTCAG *TTGGCTGGAC*GAT
**A _Barcode-88**	CCATCTCATCCCTGCGTGTCTCCGACTCAG*CCGAACACTTC*GAT
**A _Barcode-89**	CCATCTCATCCCTGCGTGTCTCCGACTCAG *TCCTGAATCTC*GAT
**A _Barcode-90**	CCATCTCATCCCTGCGTGTCTCCGACTCAG *CTAACCACGGC*GAT
**A _Barcode-91**	CCATCTCATCCCTGCGTGTCTCCGACTCAG *CGGAAGGATGC*GAT
**A _Barcode-92**	CCATCTCATCCCTGCGTGTCTCCGACTCAG *CTAGGAACCGC*GAT
**A _Barcode-93**	CCATCTCATCCCTGCGTGTCTCCGACTCAG *CTTGTCCAATC*GAT
**A _Barcode-94**	CCATCTCATCCCTGCGTGTCTCCGACTCAG *TCCGACAAGC*GAT
**A _Barcode-95**	CCATCTCATCCCTGCGTGTCTCCGACTCAG *CGGACAGATC*GAT
**A _Barcode-96**	CCATCTCATCCCTGCGTGTCTCCGACTCAG *TTAAGCGGTC*GAT

Table S2: Primers with sequences used in NGS library amplification.

**Primer**	**Sequence 5'-3'**
P1amp	CCACTACGCCTCCGCTTTCCTCTCTATG
T_PCR_A	CCATCTCATCCCTGCGTGTC

Collection of Images S1: Photos of individual phenotypes within varitey 'Carmagnola selected', 'Tiborszallasi' and 'Finola selection' based on visual traits.

Phenotype CI ('Carmagnola selected')

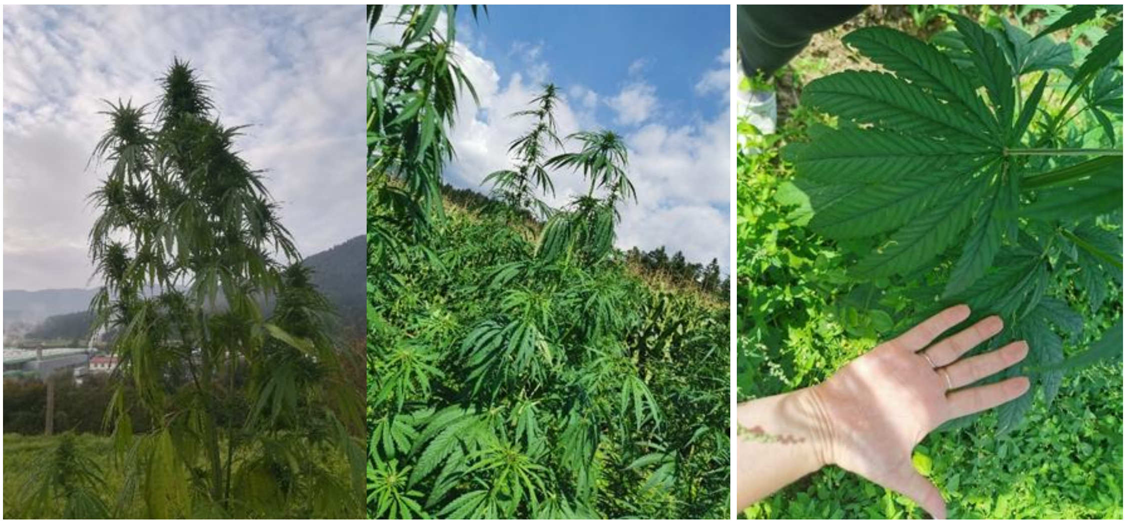

Phenotype CII ('Carmagnola selected')

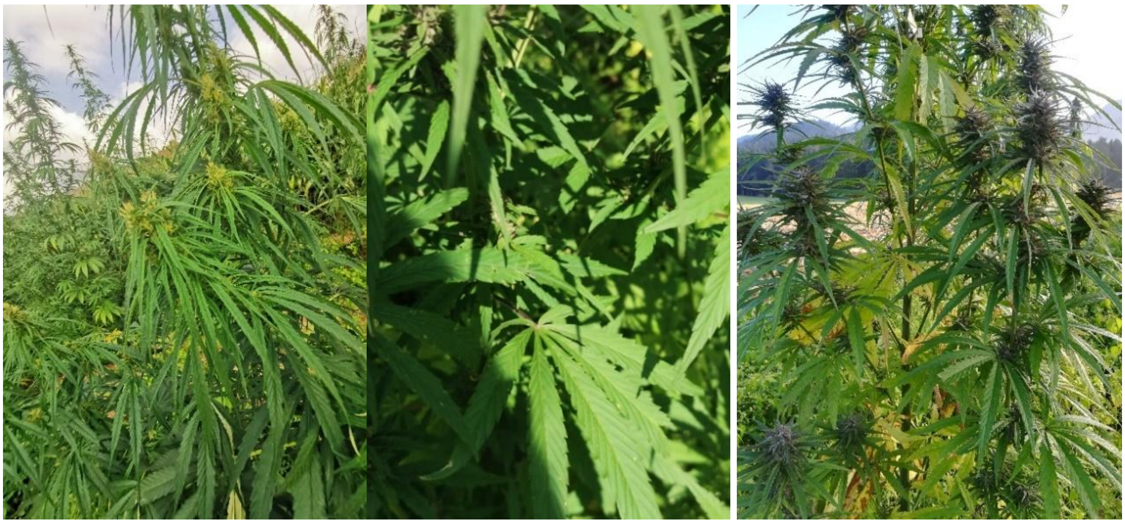

Phenotype TI ('Tiborszallasi')

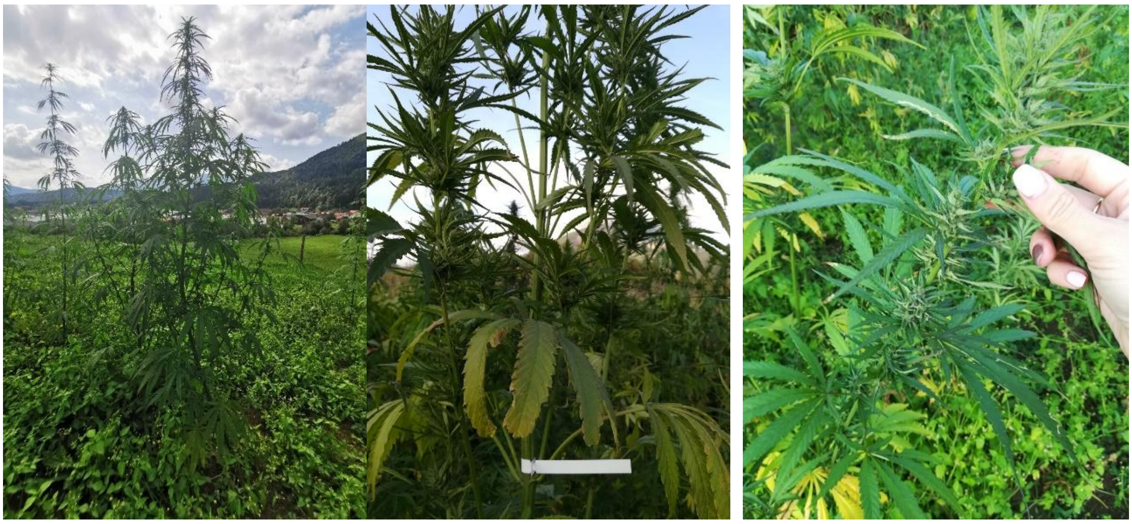

Phenotype TII ('Tiborszallasi')

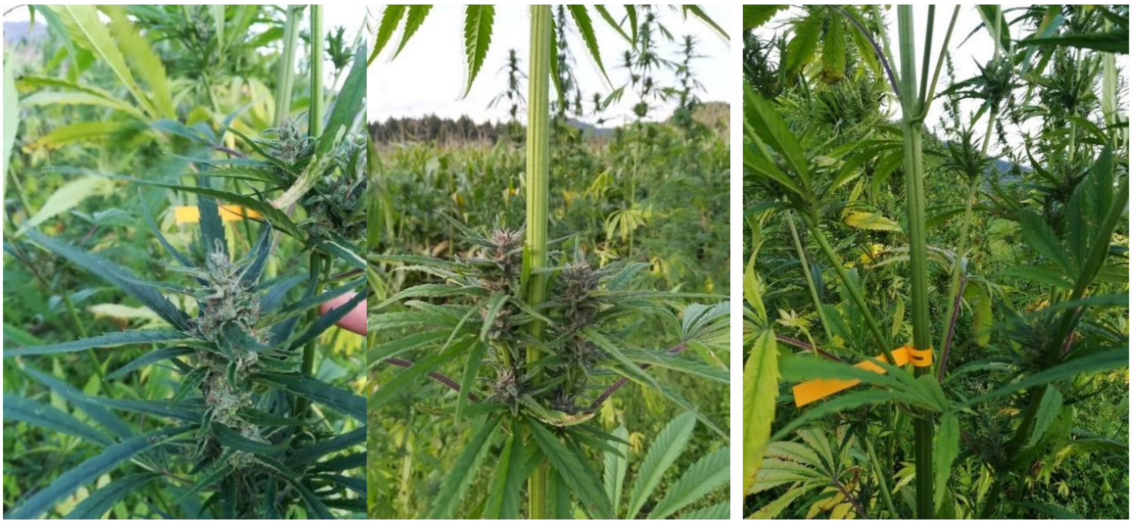

Phenotype TIII ('Tiborszallasi')

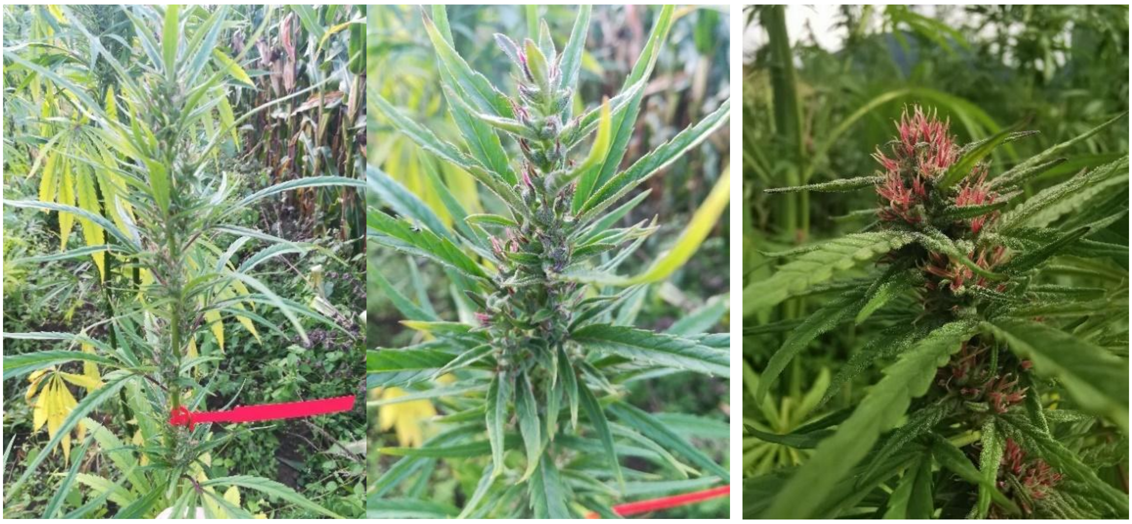

Phenotpye TIV ('Tiborszallasi')

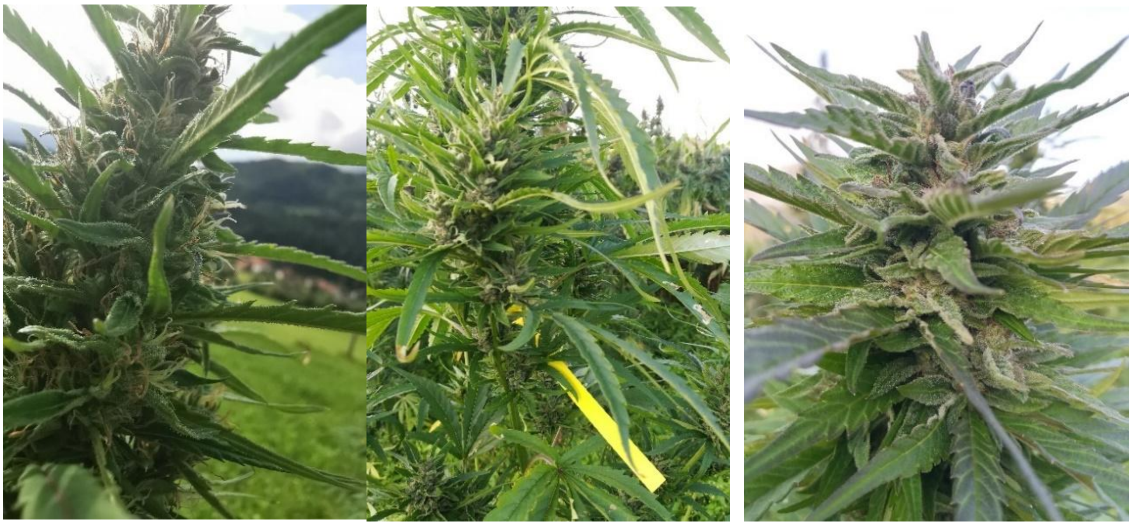

Phenotype TV ('Tiborszallasi')

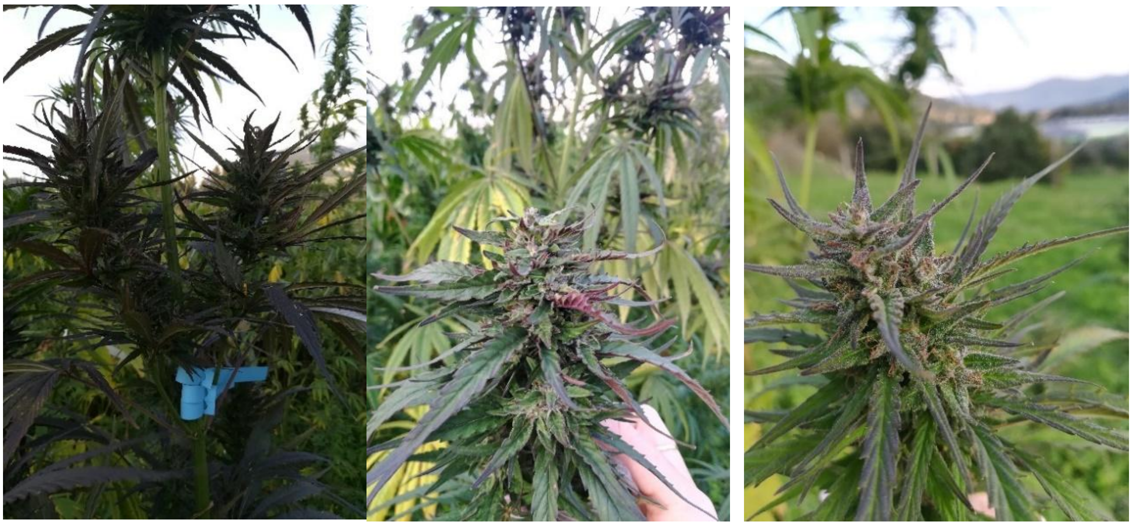
Phenotype FI ('Finola selection')

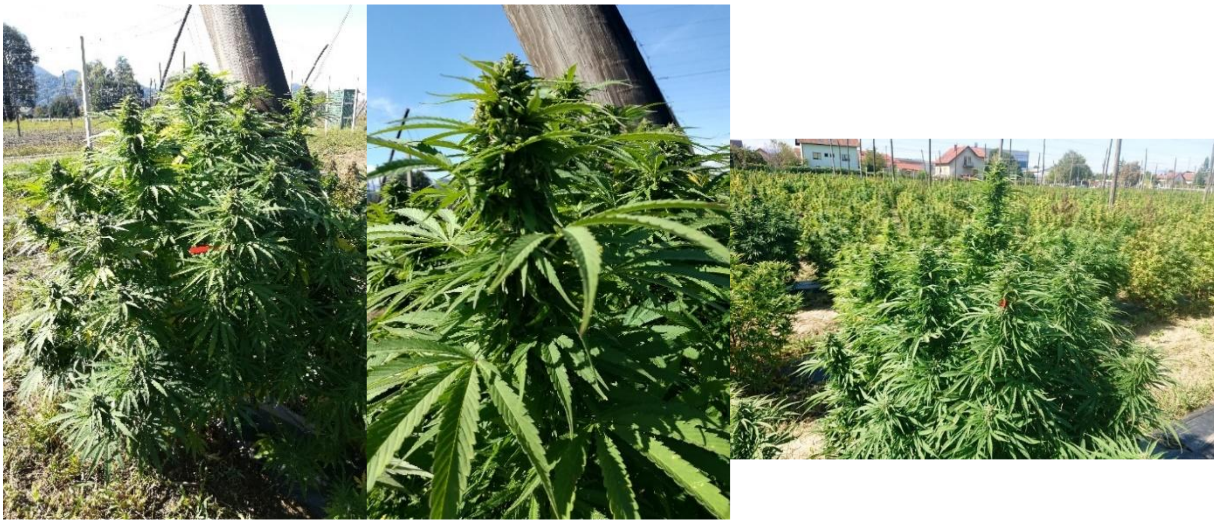
Phenotype FII ('Finola selection')

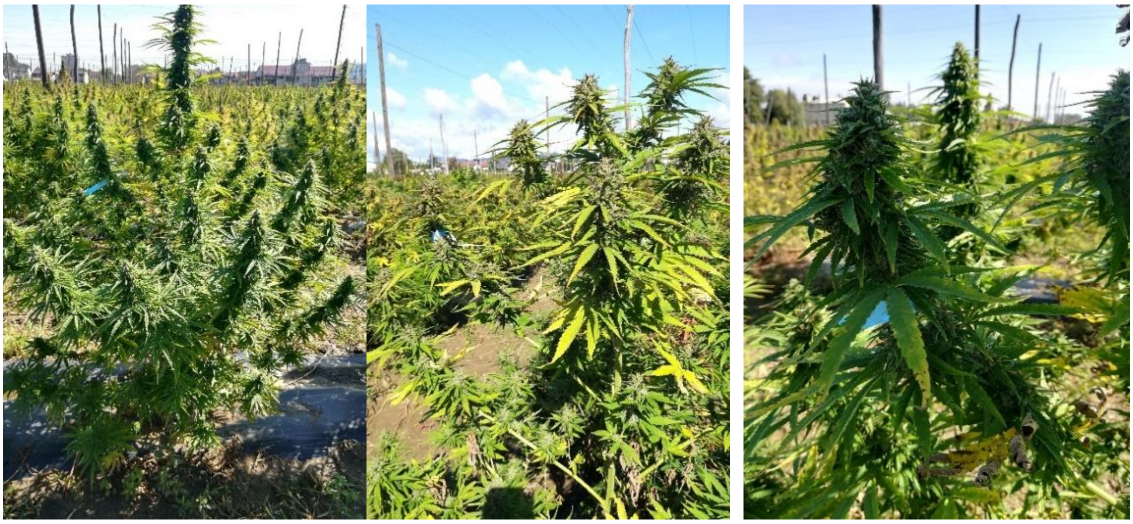
Phenotype FIII ('Finola selection')

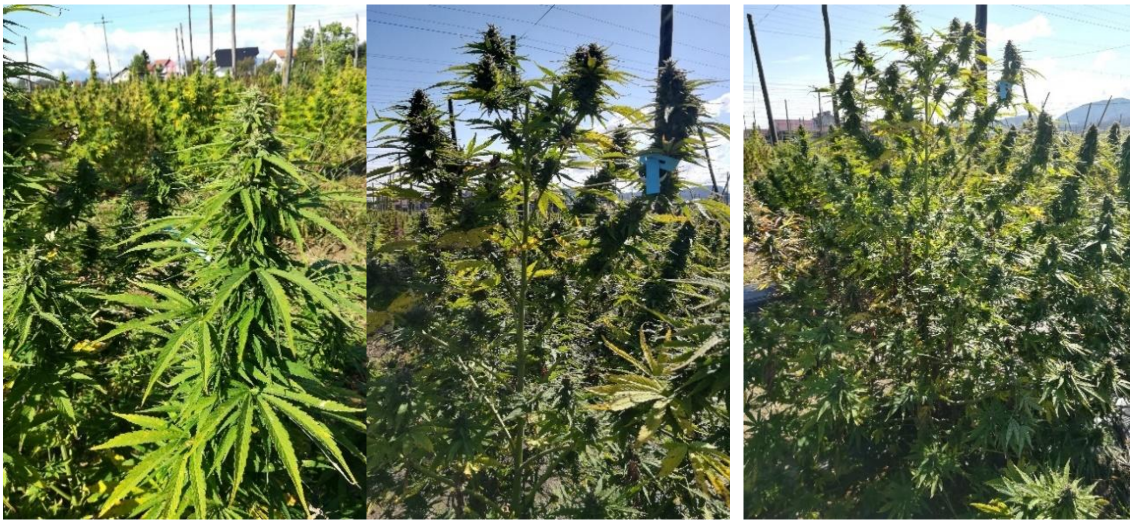

Phenotype FIV ('Finola selection')

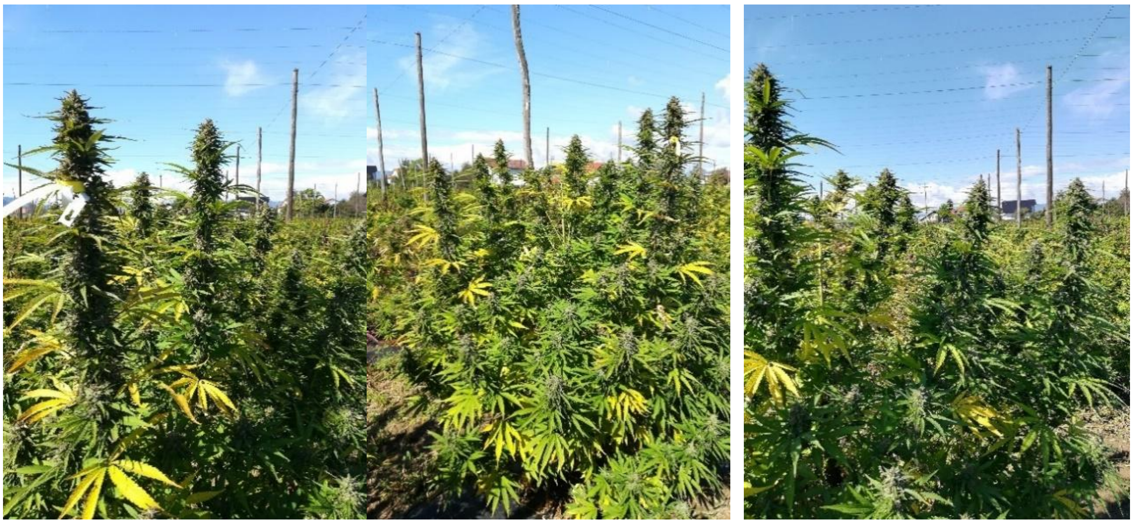

File S1: SNP calling

SNP calling was performed using the Genome Analysis Toolkit (GATK) (https://gatk.broadinstitute.org/hc/en-us), following the GATK Best Practices Workflows (https://gatk.broadinstitute.org/hc/en-us/sections/360007226651-Best-Practices-Workflows). The procedure included the following steps:

1 Creating a sequence dictionary file from a reference sequence using the 'CreateSequenceDictionary'.

Reference genome in FASTA format was in file `GCA_900626175_seqret.fasta`

command: `gatk CreateSequenceDictionary -R GCA_900626175_seqret.fasta`

2 Identification of duplicates using 'MarkDuplicates'.

The file `MarkDuplicates.bash` was wrtitten (below), which was run with a bash command `MarkDuplicates.bash`

```
for f in *.bam; do

base=${f%.bam}
```

```
gatk MarkDuplicates -I »$f« -O »$base«_marked_duplicates.bam -M »$base«_marked_duplicates_metrics.txt 1>«$base«_markduplicates.1log 2>«$base«_markduplicates.log

done
```

3 Sorting of BAM files using 'SortSam'.

FIle `SortSam.bash` was written (bellow) which was run with a bash command `SortSam.bash`

```
for f in *_marked_duplicates.bam; do

base=${f%.bam}

gatk SortSam -I $f -O »$base«_sorted.bam -SORT_ORDER coordinate 1>«$base«_sortsam.1log 2>«$base«_sortsam.2log

done
```

4 Recalibration of qualitative values using the 'BaseRecalibrator' according to known SNP postitions and tool 'ApplyBQSR.bash'.

For known 4537 SNP positions file in VCF format `4537_pozicij.vcf`.was used.

File `BaseRecalibrator.bash` was written (below), which was run with a bash command `BaseRecalibrator.bash`

```
for f in *_sorted.bam; do

base=${f%.bam}

gatk BaseRecalibrator -I »$f« -R GCA_900626175_seqret.fasta –known-sites 4537_pozicij.vcf -O »$base«_recal_data.table

done
```

File `ApplyBQSR.bash` was written (below), which was run with a bash command `ApplyBQSR.bash`

```
for f1 in *_marked_duplicates_sorted.bam; do

base1=${f1%.bam}

base2=«${base1%_marked_duplicates_sorted}«

for f2 in *marked_duplicates_sorted_recal_data.table; do

base3=${f2%.table}

if [ »$base1« == »${base3%_recal_data}« ]; then

gatk ApplyBQSR -R GCA_900626175_seqret.fasta -I »$f1« –bqsr-recal-file »$f2« -O »$base2«_processed.bam 1>«$base2«_ApplyBQSR1.log 2>«$base2«_ApplyBQSR2.log

fi

done

done
```

5 Determination of SNP postitions using 'HaplotypeCaller'.

File `HaplotypeCaller.bash` was written (below), which was run with a bash command `HaplotypeCaller.bash`

```
for f in *_processed.bam; do
base1=${f%.bam}
base2=${base1%_processed}
gatk HaplotypeCaller -R GCA_900626175_seqret.fasta -I »$f« -O
»$base2«.vcf -ERC GVCF –native-pair-hmm-threads 150
1>«$base2«_HaplotypeCaller.log1 2>«$base2«_HaplotypeCaller.log2
done
```

6 Combining individual VCF files to one VCF file using the 'CombineGVCFs'.

File `CombineGVCFs.bash` was written (below), which was run with a bash command `CombineGVCFs.bash`

```
gatk CombineGVCFs \
-R ../GCA_900626175_seqret.fasta –variant ../CI1.vcf –variant
../CI2.vcf –variant ../CI3.vcf –variant ../CI4.vcf –variant
../CI5.vcf –variant ../CI6.vcf \ --variant ../CI7.vcf \ --variant
../CI8.vcf \ --variant ../CI9.vcf \ --variant ../CI10.vcf \ --
variant ../CI11.vcf \ --variant ../CI12.vcf \ --variant ../CI13.vcf
\ --variant ../CI14.vcf \ --variant ../CI15.vcf \ --variant
../CI16.vcf \ --variant ../CI18.vcf \ --variant ../CI19.vcf \ --
variant ../CII1.vcf \ --variant ../CII2.vcf \ --variant ../CII3.vcf
\ --variant ../CII4.vcf \ --variant ../CII5.vcf \ --variant
../CII6.vcf \ --variant ../CII7.vcf \ --variant ../CII8.vcf \ --
variant ../CII9.vcf \ --variant ../CII10.vcf \ --variant
../CII11.vcf \ --variant ../CII12.vcf \ --variant ../CII13.vcf \ --
variant ../CII14.vcf \ --variant ../CII15.vcf \ --variant
../CII16.vcf \ --variant ../CII17.vcf \ --variant ../CII18.vcf \ --
variant ..–II19.vcf \ --variant ../FI1.vcf \ --variant ../FI2.vcf \
--variant ../FI3.vcf \ --variant ../FI4.vcf \ --variant ..–I5.vcf \
--variant ../FI6.vcf \ --variant ../FI7.vcf \ --variant ../FI9.vcf \
--variant ../FI10.vcf \ --variant ../FI11.vcf \ --variant
../FI12.vcf \ --variant ../FI13.vcf \ --variant ../FI14.vcf \ --
variant ../FI15.vcf \ --variant ../FI16.vcf \ --variant ../FI17.vcf
\ --variant ../FI18.vcf \ --variant ../FI20.vcf \ --variant
../FII1.vcf \ --variant ../FII2.vcf \ --variant ../FII3.vcf \ --
variant ../FII4.vcf \ --variant ../FII5.vcf \ --variant ../FII6.vcf
\ --variant ../FII7.vcf \ --variant ../FII8.vcf \ --variant
../FII9.vcf \ --variant ../FII10.vcf \ --variant ../FII11.vcf \ --
variant ../FII12.vcf \ --variant ../FII14.vcf \ --variant
../FII15.vcf \ --variant ../FII16.vcf \ --variant ../FII17.vcf \ --
variant ../FII18.vcf \ --variant ../FII19.vcf \ --variant
../FII20.vcf \--variant ../FIII1.vcf \ --variant ../FIII2.vcf \ --
variant ../FIII3.vcf \ --variant ../FIII4.vcf \ --variant
../FIII5.vcf \ --variant ../FIII6.vcf \ --variant ../FIII7.vcf \ --
variant ../FIII8.vcf \ --variant ../FIII9.vcf \ --variant
../FIII10.vcf \ --variant ../FIII11.vcf \ --variant ../FIII12.vcf \
--variant ../FIII13.vcf \ --variant ..–III14.vcf \ --variant
../FIII15.vcf \ --variant ../FIII16.vcf \ --variant ../FIII17.vcf \
```

```
--variant ../FIII18.vcf \ --variant ../FIII19.vcf \ --variant
../FIII20.vcf \ --variant ../FIV1.vcf \ --variant ../FIV2.vcf \ --
variant ../FIV3.vcf \ --variant ../FIV4.vcf \ --variant ../FIV5.vcf
\ --variant ../FIV6.vcf \ --variant ../FIV7.vcf \ --variant
../TI1.vcf \ --variant ../TI2.vcf \ --variant ..-I3.vcf \ --variant
../TI4.vcf \ --variant ../TI5.vcf \ --variant ../TI6.vcf \ --variant
../TI7.vcf \ --variant ../TI8.vcf \ --variant ../TI9.vcf \ --variant
../TI10.vcf \ --variant ../TI11.vcf \ --variant ../TI12.vcf \ --
variant ../TI13.vcf \ --variant ../TI14.vcf \ --variant ../TI15.vcf
\ --variant ../TI17.vcf \ --variant ../TI18.vcf \ --variant
../TI19.vcf \ --variant ../TI20.vcf \ --variant ../TII1.vcf \ --
variant ../TII2.vcf \ --variant ../TII3.vcf \ --variant ../TII4.vcf
\ --variant ../TII5.vcf \ --variant ../TII6.vcf \ --variant
../TII7.vcf \ --variant ../TII8.vcf \ --variant ../TII9.vcf \ --
variant ../TII10.vcf \ --variant ../TII11.vcf \ --variant
../TII12.vcf \ --variant ../TII13.vcf \ --variant ..-II14.vcf \ --
variant ../TII15.vcf \ --variant ../TII16.vcf \ --variant
../TII17.vcf \ --variant ../TII18.vcf \ --variant ..-II19.vcf \ --
variant ../TII20.vcf \ --variant ../TIII1.vcf \ --variant
../TIII2.vcf \ --variant ../TIII3.vcf \ --variant ../TIII4.vcf \ --
variant ../TIII5.vcf \ --variant ../TIII6.vcf \ --variant
../TIII7.vcf \ --variant ../TIII8.vcf \ --variant ../TIII9.vcf \ --
variant ../TIII11.vcf \ --variant ../TIII12.vcf \ --variant
../TIII13.vcf \ --variant ..-III14.vcf \ --variant ../TIV1.vcf \ --
variant ../TIV2.vcf \ --variant ../TIV3.vcf \ --variant ../TIV4.vcf
\ --variant ..-IV5.vcf \ --variant ../TIV6.vcf \ --variant
../TIV7.vcf \ --variant ../TIV8.vcf \ --variant ../TIV9.vcf \ --
variant ..-IV10.vcf \ --variant ../TIV11.vcf \ --variant
../TIV12.vcf \ --variant ../TIV13.vcf \ --variant ../TV1.vcf \ --
variant ../TV3.vcf \ --variant ../TV4.vcf \ --variant ../TV5.vcf \ -
-variant ../TV6.vcf -O combined_all.vcf 1>1.log 2>2.log &
```

7 Samples genotyping using 'GenotypeGVCFs'.

genotyping of samples occurred according to 4,537 analyzed SNP positions (file `4537_pozicij.vcf`). Data for monomorphic positions were kept using the `–include-non-variant-sites`

Run bash command:

```
gatk GenotypeGVCFs -R GCA_900626175_seqret.fasta -V combined_all.vcf
-O combined_all_genotyped.vcf -L 4537_pozicij.vcf –include-non-
variant-sites
```

8 Data were filtered according to the quality and depth of reads using 'VariantFiltration'

Run bash command: *gatk VariantFiltration -R GCA_900626175_seqret.fasta -V*

combined_all_genotyped.vcf --filter-expression "DP < 5" --

filter-name "LowQual" --filter-expression "QUAL < 20" --

filter-name "LowQual20" -O test_filtered.vcf

Supplementary Data S1: 10.6084/m9.figshare.30762896
Supplementary Data S2: https://doi.org/10.6084/m9.figshare.23761965
Supplementary Data S3: https://doi.org/10.6084/m9.figshare.23763873
Supplementary Data S4: https://doi.org/10.6084/m9.figshare.23764314
Supplementary Data S5: https://doi.org/10.6084/m9.figshare.23764482
Supplementary Data S6: https://doi.org/10.6084/m9.figshare.23765337
