# Supplementary figures and images for: Population Structure of Genotypes and Genome-Wide Association Studies of Cannabinoids and Terpenes Synthesis in Hemp (Cannabis sativa L.)
Source: Plants (Basel). 2026 Jan 8;15(2):202. doi: 10.3390/plants15020202 (PMC12845109; doi:10.3390/plants15020202)

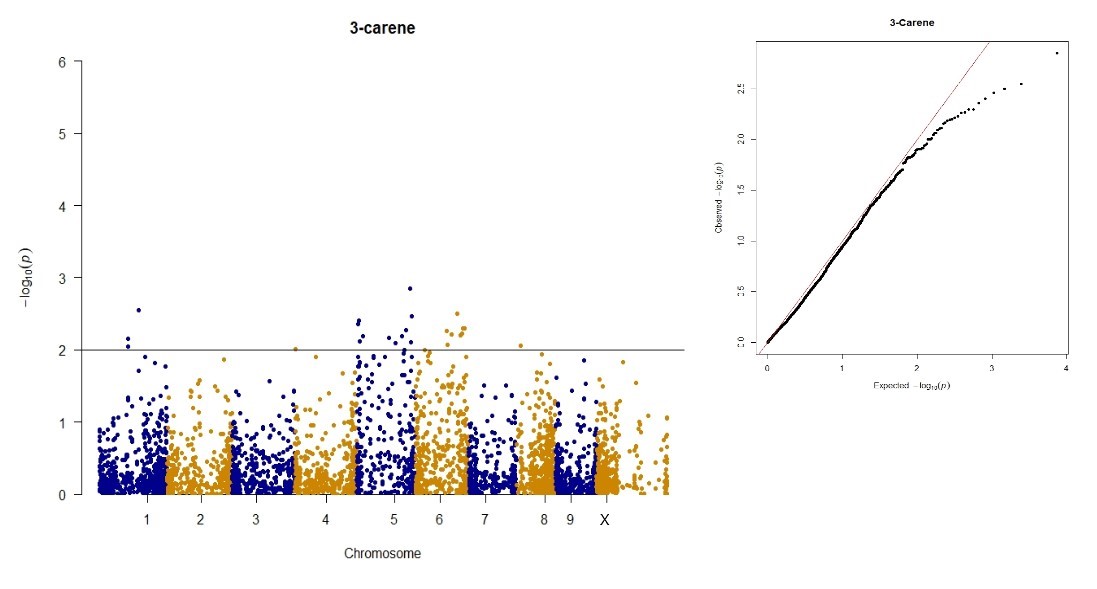

Supplement: Supplementary file 1 [file plants-15-00202-s001.zip › Manhattan plots/Figure S1.3-carene.jpg]

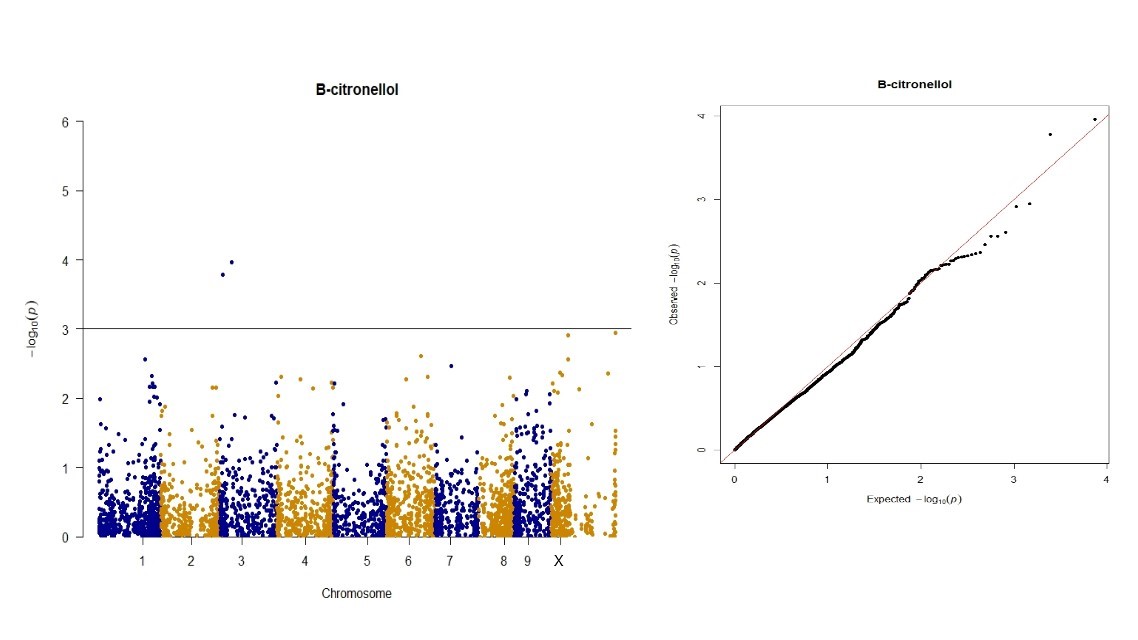

Supplement: Supplementary file 1 [file plants-15-00202-s001.zip › Manhattan plots/Figure.S.10.B-citronellol.jpg]

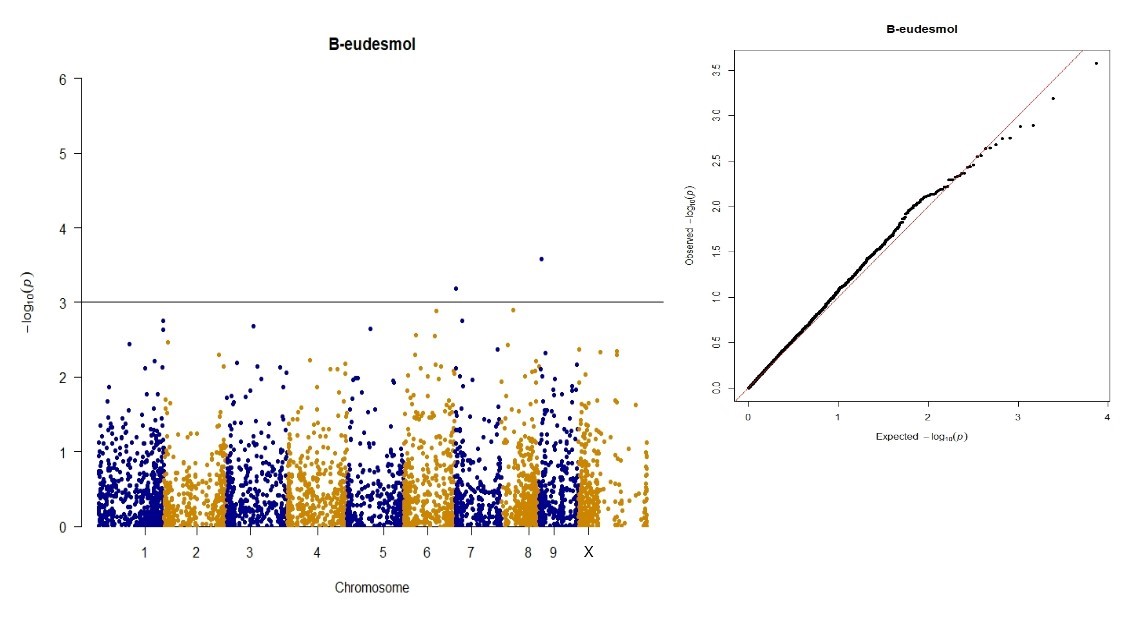

Supplement: Supplementary file 1 [file plants-15-00202-s001.zip › Manhattan plots/Figure.S11.B-eudesmol.jpg]

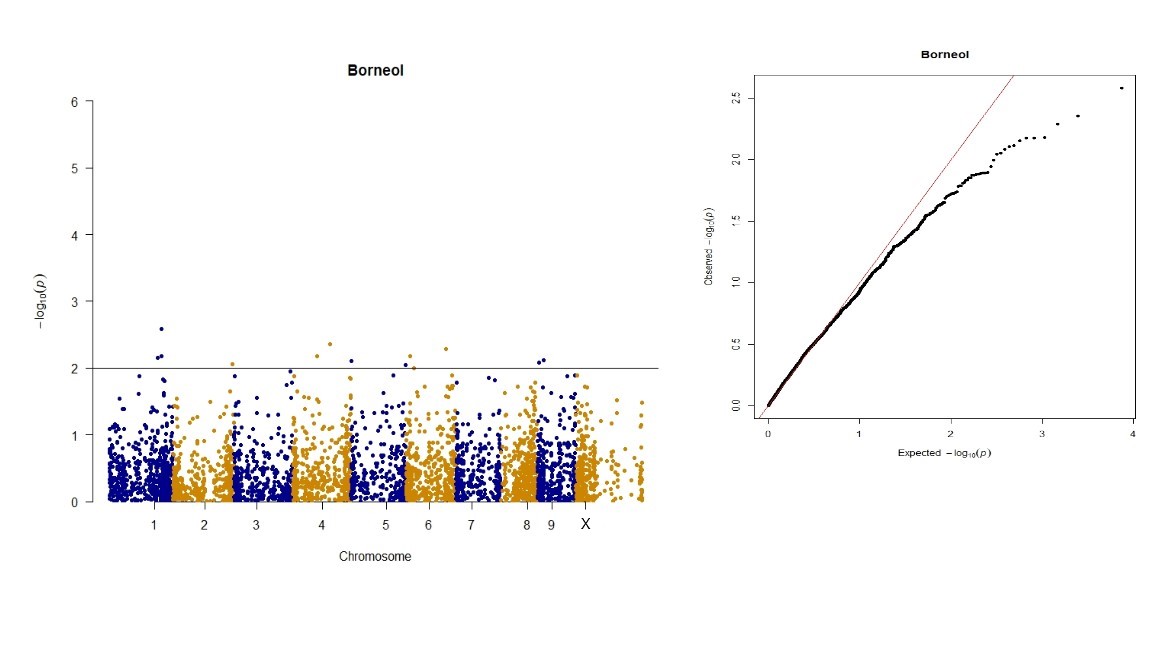

Supplement: Supplementary file 1 [file plants-15-00202-s001.zip › Manhattan plots/Figure.S12.Borneol.jpg]

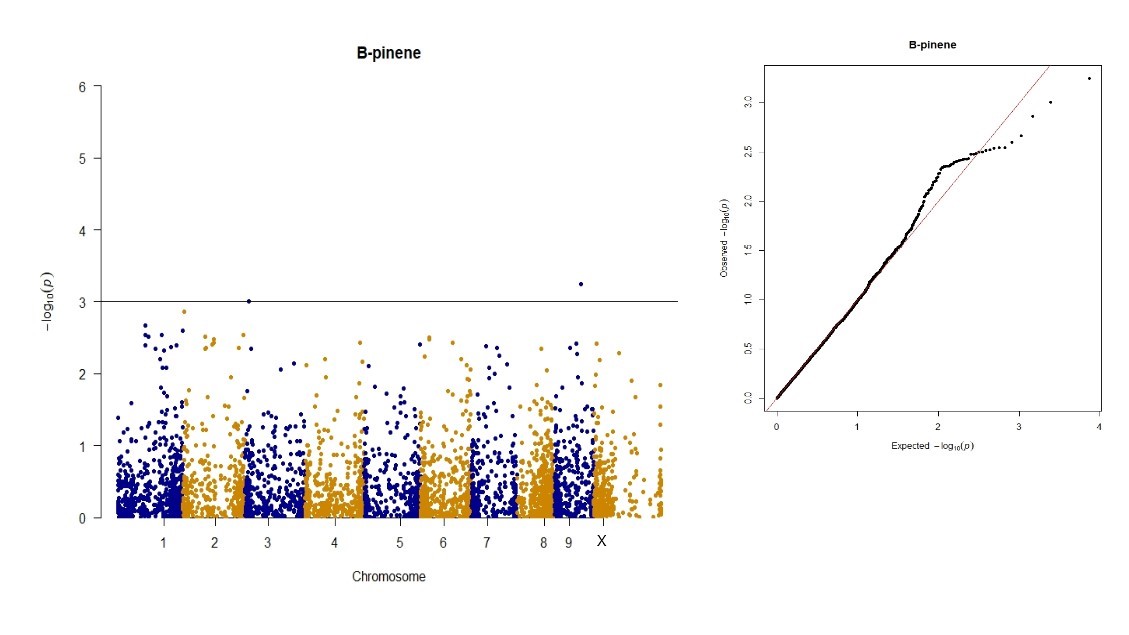

Supplement: Supplementary file 1 [file plants-15-00202-s001.zip › Manhattan plots/Figure.S13.B-pinene.jpg]

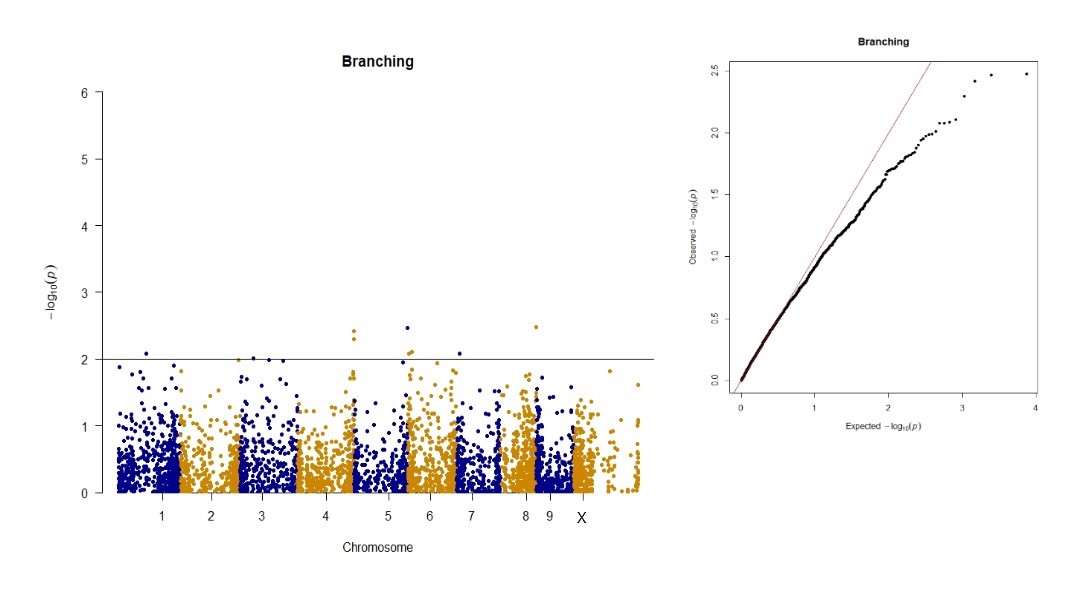

Supplement: Supplementary file 1 [file plants-15-00202-s001.zip › Manhattan plots/Figure.S14.Branching.jpg]

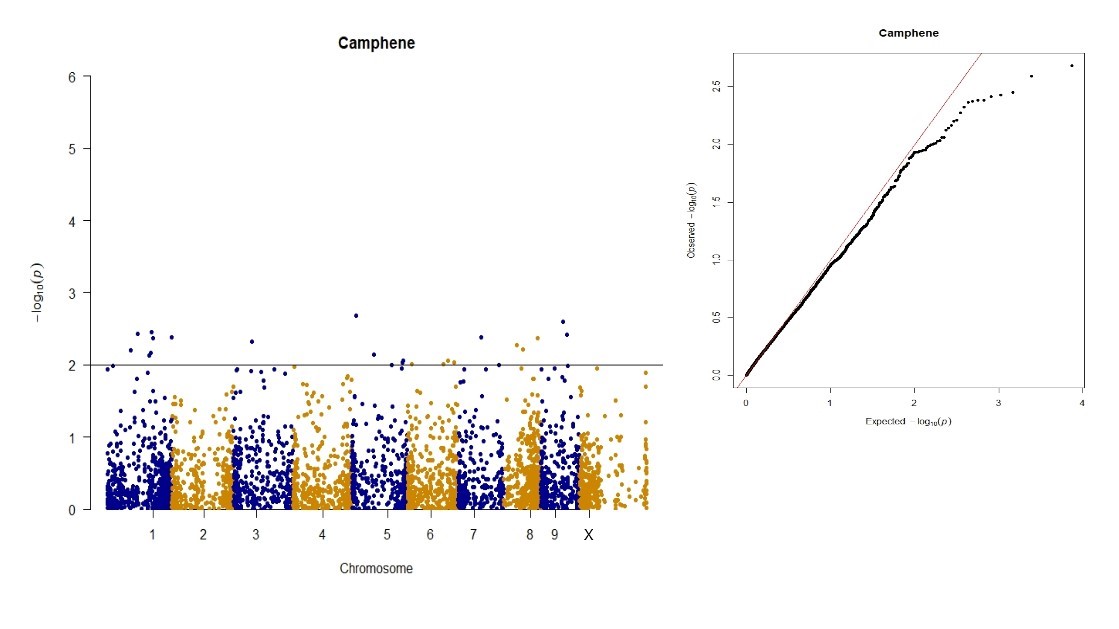

Supplement: Supplementary file 1 [file plants-15-00202-s001.zip › Manhattan plots/Figure.S15.Camphene.jpg]

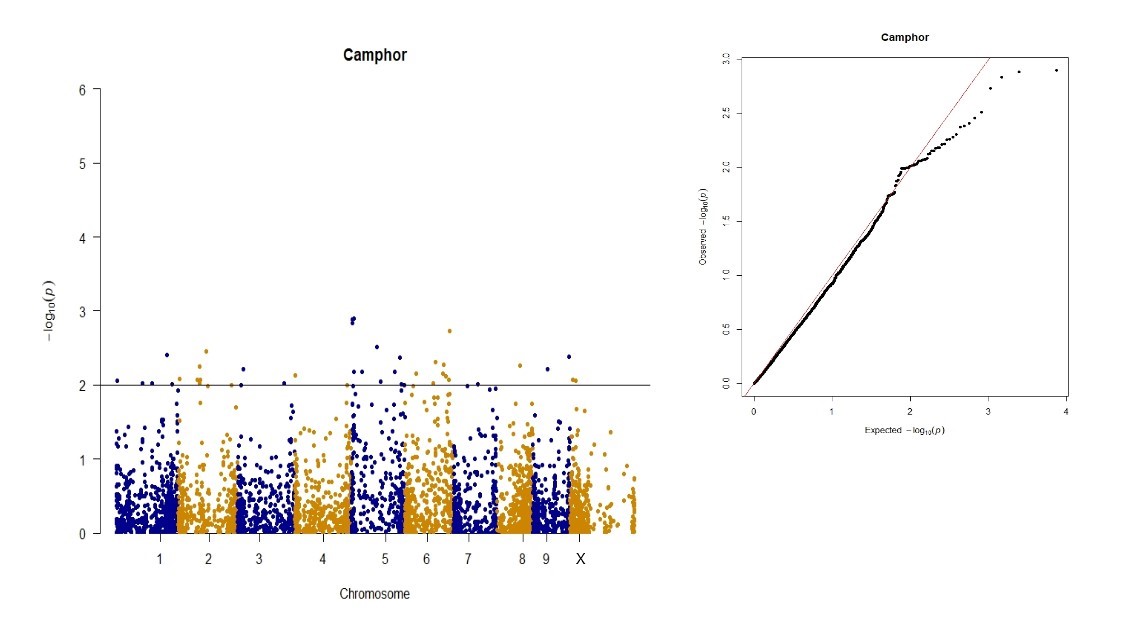

Supplement: Supplementary file 1 [file plants-15-00202-s001.zip › Manhattan plots/Figure.S16.Camphor.jpg]

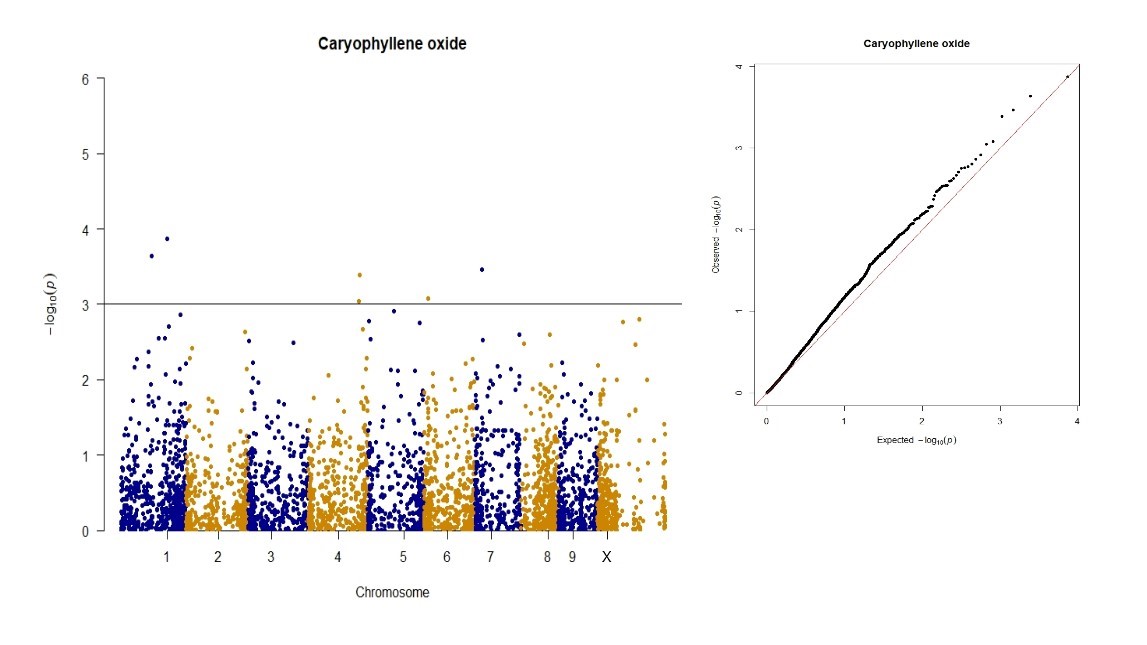

Supplement: Supplementary file 1 [file plants-15-00202-s001.zip › Manhattan plots/Figure.S17.Caryophyllene oxide.jpg]

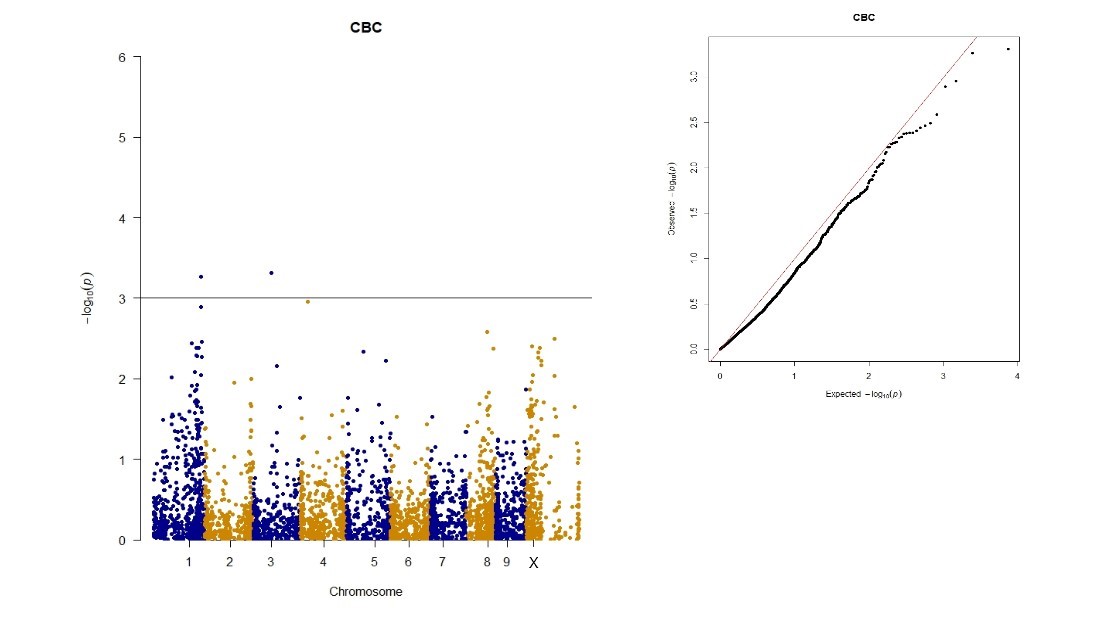

Supplement: Supplementary file 1 [file plants-15-00202-s001.zip › Manhattan plots/Figure.S18.CBC.jpg]

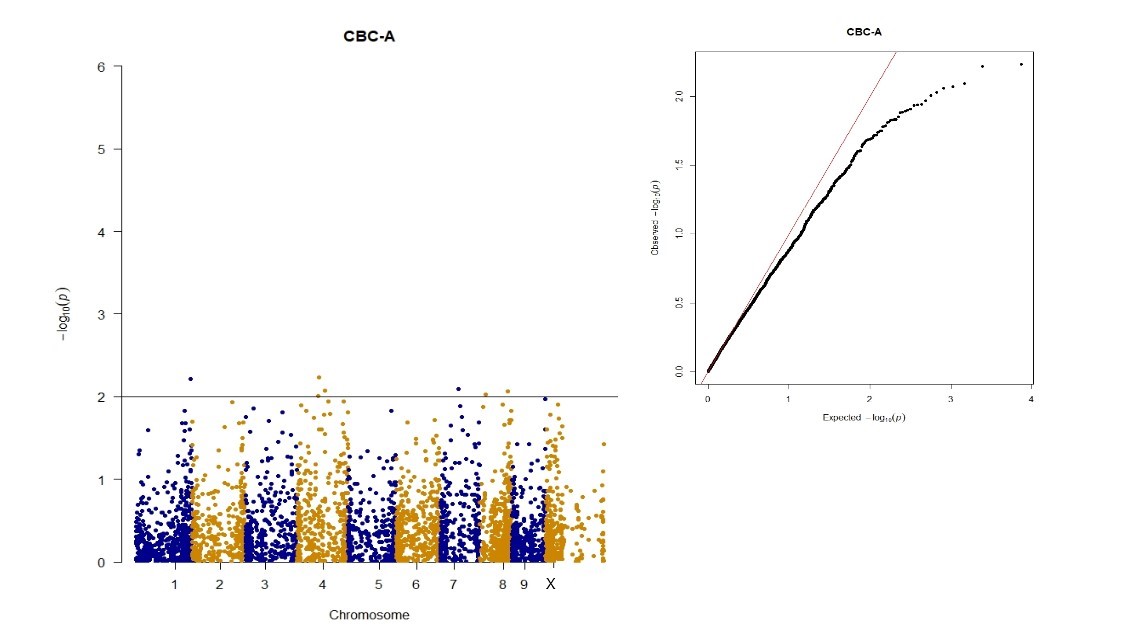

Supplement: Supplementary file 1 [file plants-15-00202-s001.zip › Manhattan plots/Figure.S19.CBC-A.jpg]

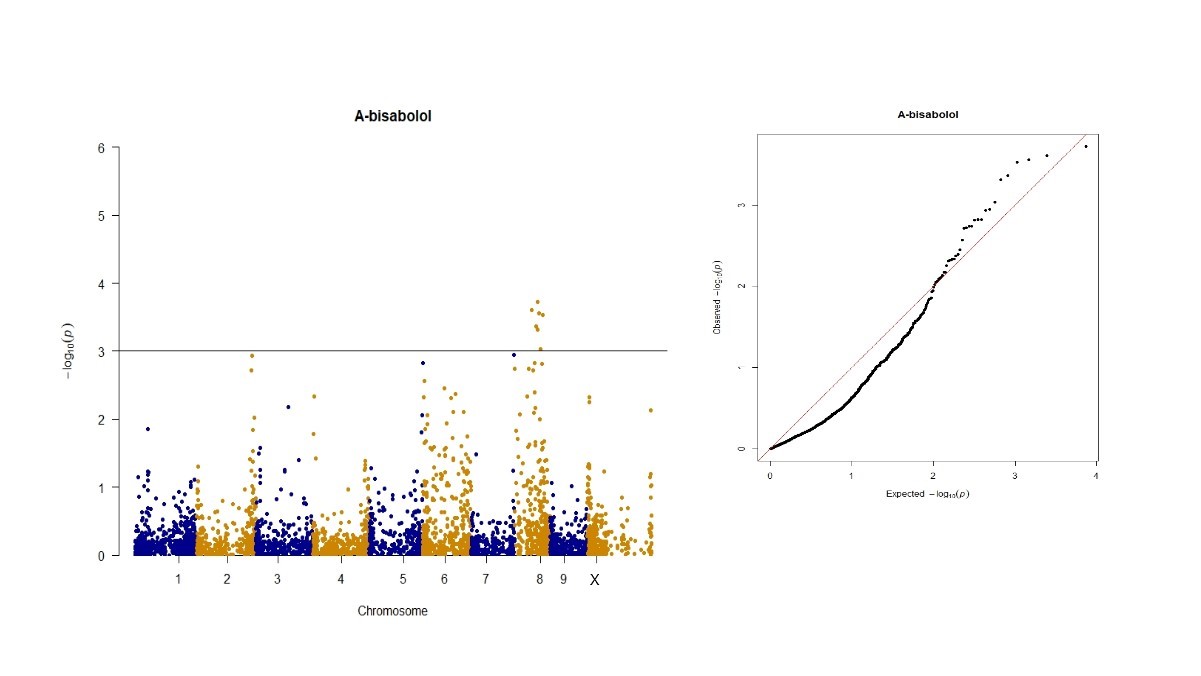

Supplement: Supplementary file 1 [file plants-15-00202-s001.zip › Manhattan plots/Figure.S2.A-bisabolol.jpg]

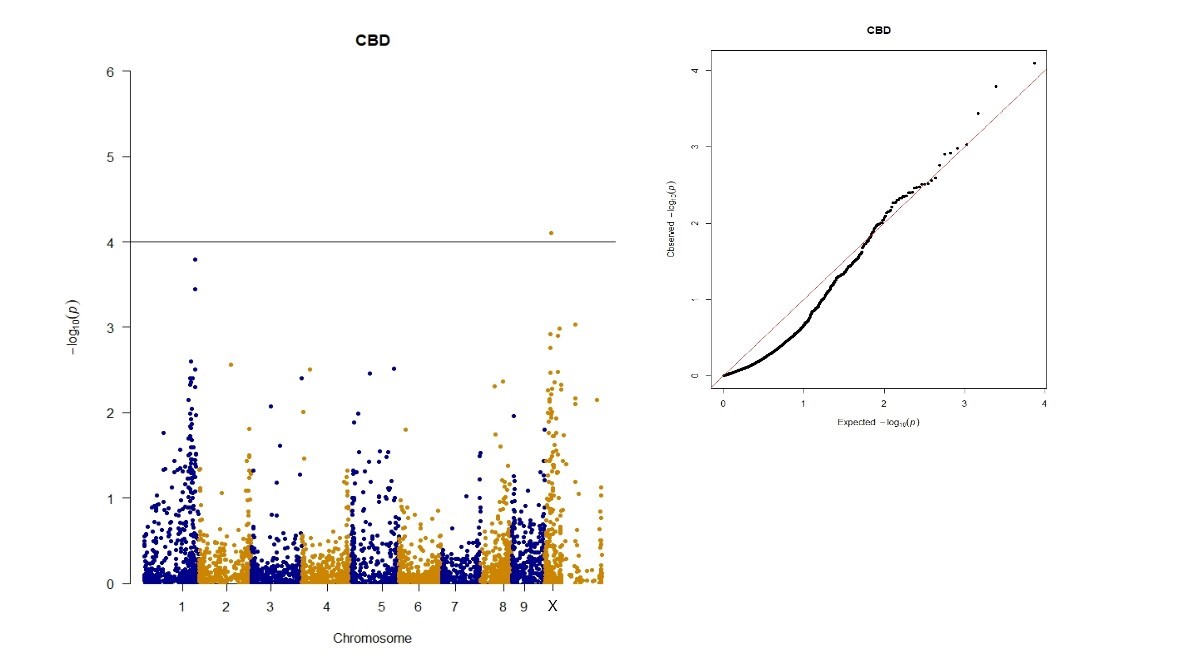

Supplement: Supplementary file 1 [file plants-15-00202-s001.zip › Manhattan plots/Figure.S20.CBD.jpg]

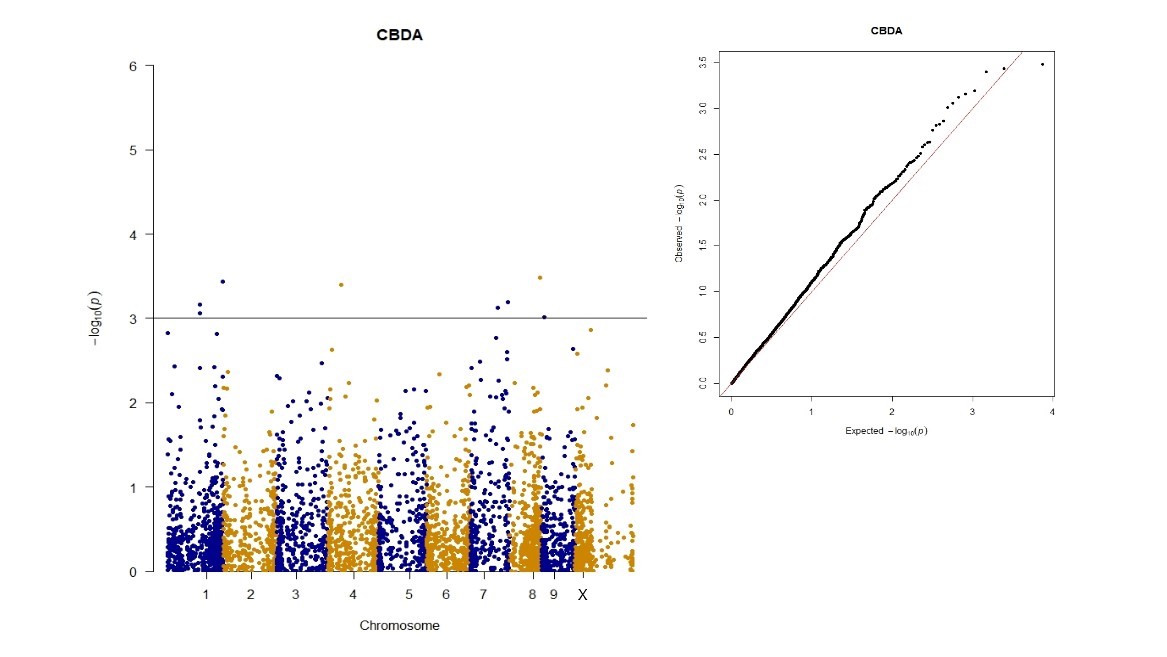

Supplement: Supplementary file 1 [file plants-15-00202-s001.zip › Manhattan plots/Figure.S21.CBD-A.jpg]

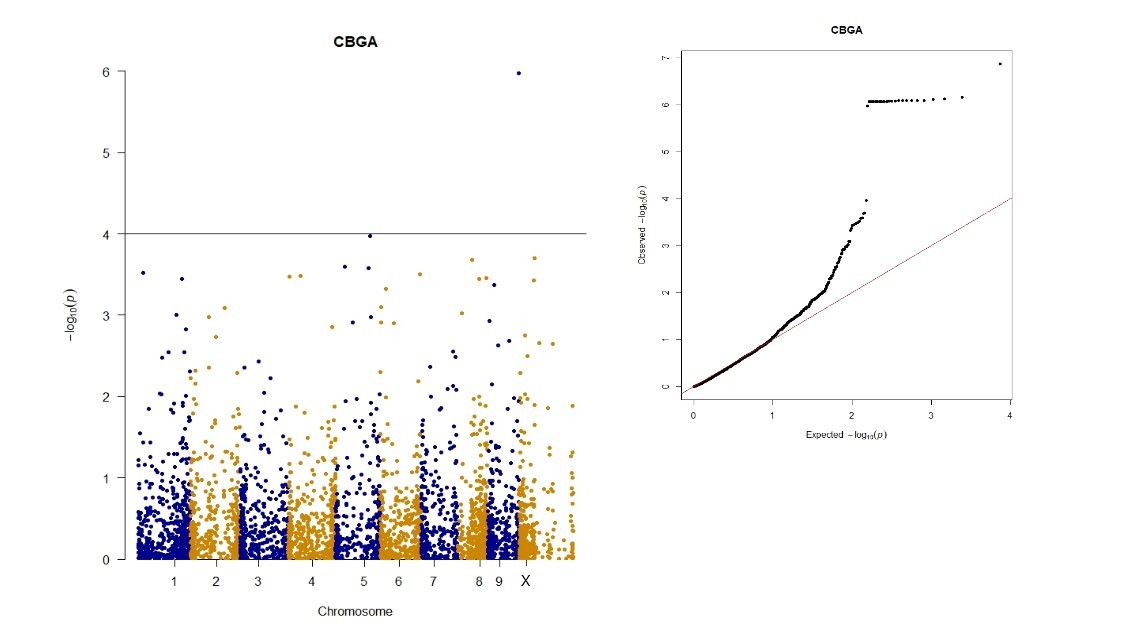

Supplement: Supplementary file 1 [file plants-15-00202-s001.zip › Manhattan plots/Figure.S22.CBG-A.jpg]

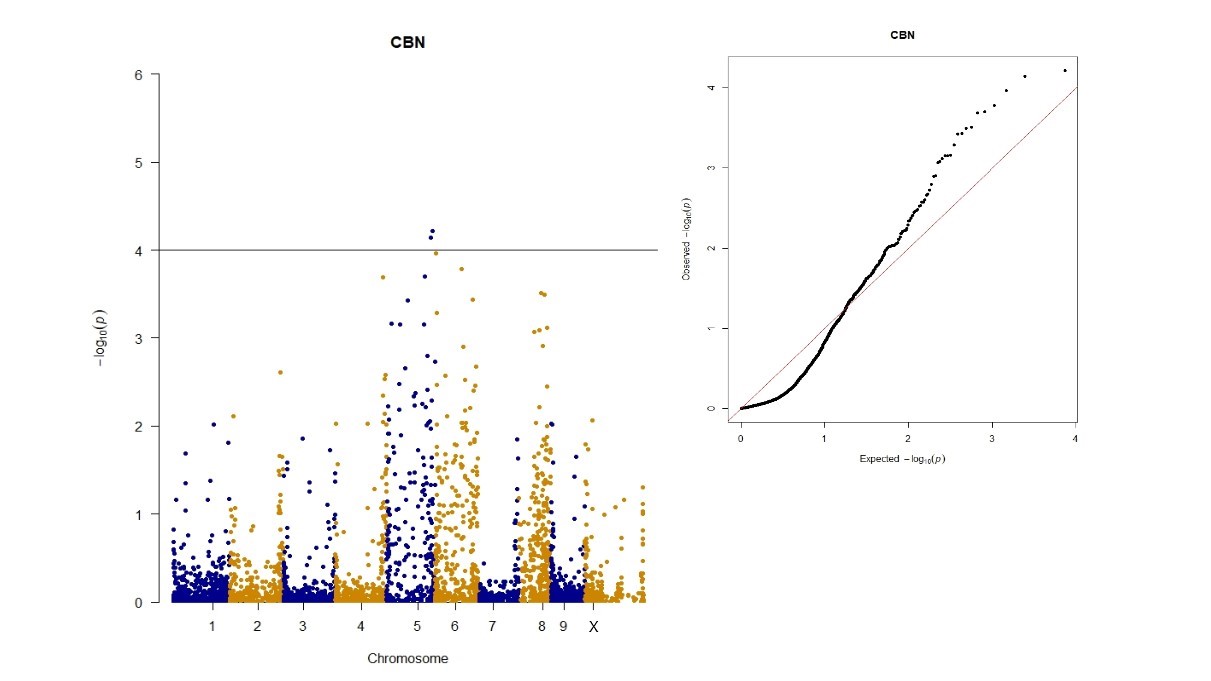

Supplement: Supplementary file 1 [file plants-15-00202-s001.zip › Manhattan plots/Figure.S23.CBN.jpg]

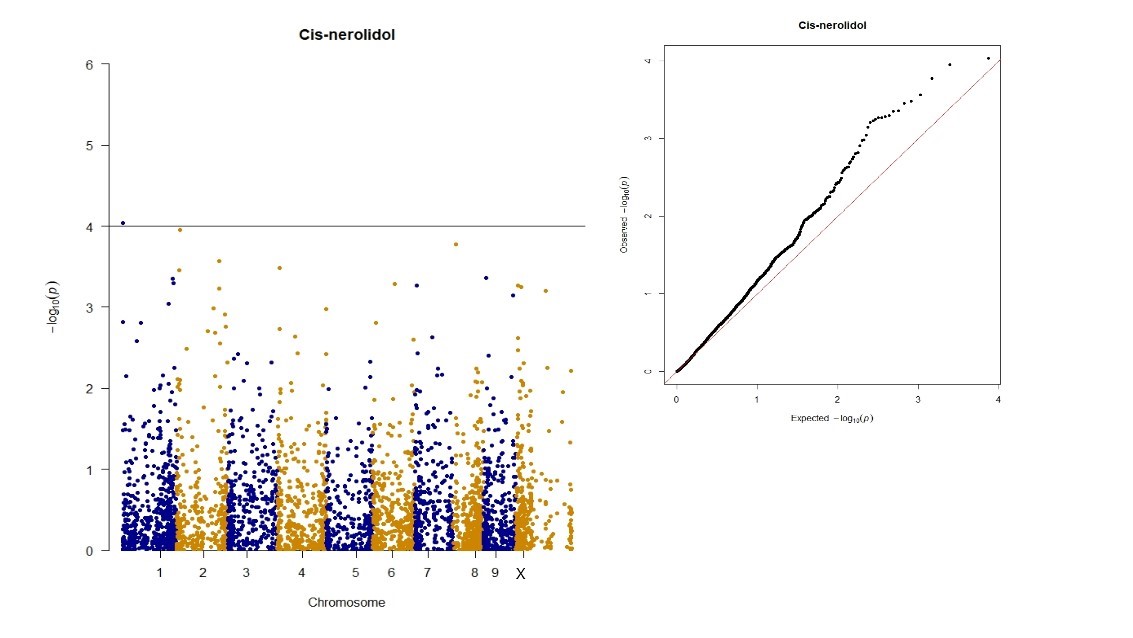

Supplement: Supplementary file 1 [file plants-15-00202-s001.zip › Manhattan plots/Figure.S24.Cis-nerolidol.jpg]

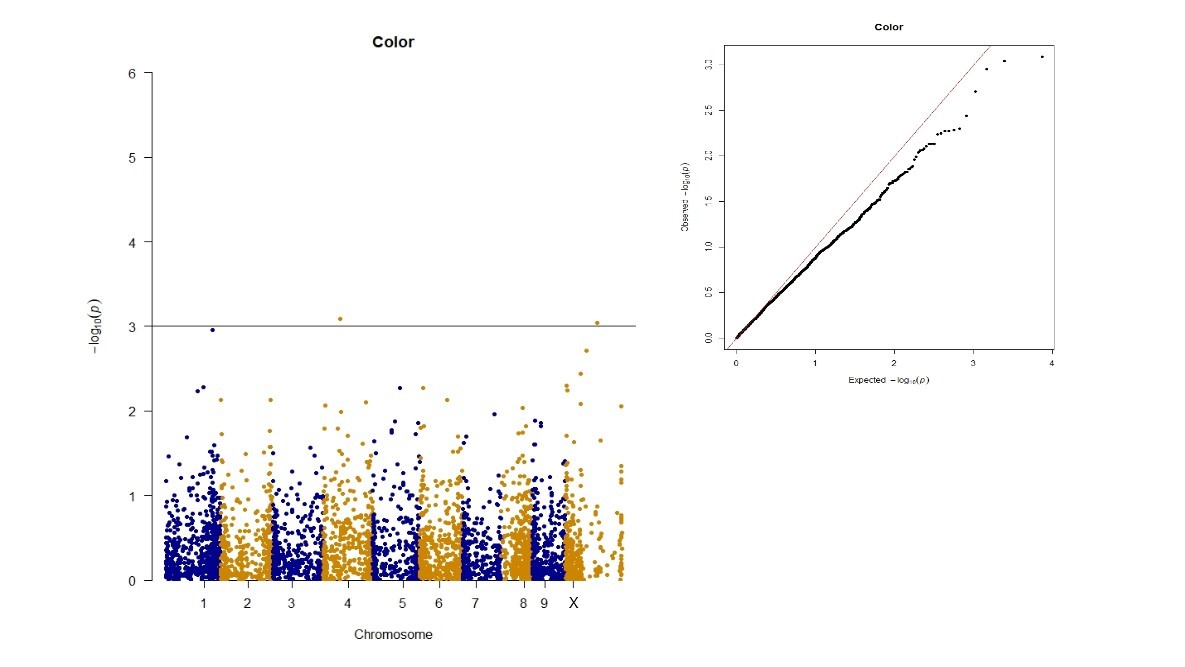

Supplement: Supplementary file 1 [file plants-15-00202-s001.zip › Manhattan plots/Figure.S25.Color.jpg]

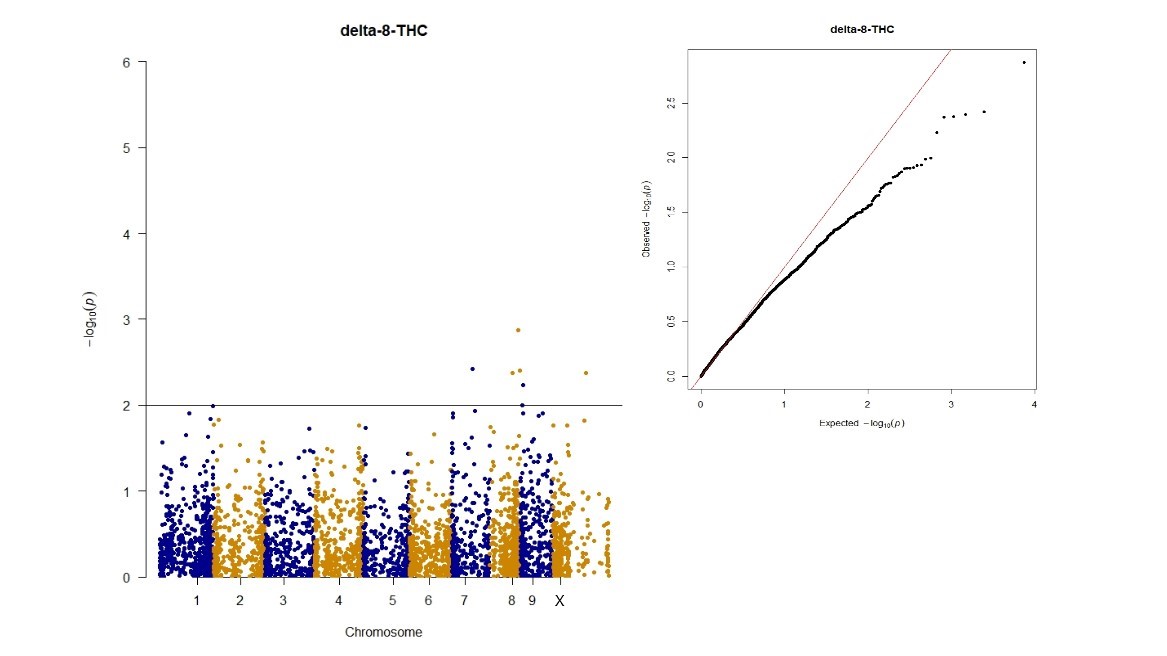

Supplement: Supplementary file 1 [file plants-15-00202-s001.zip › Manhattan plots/Figure.S26.d-8-THC.jpg]

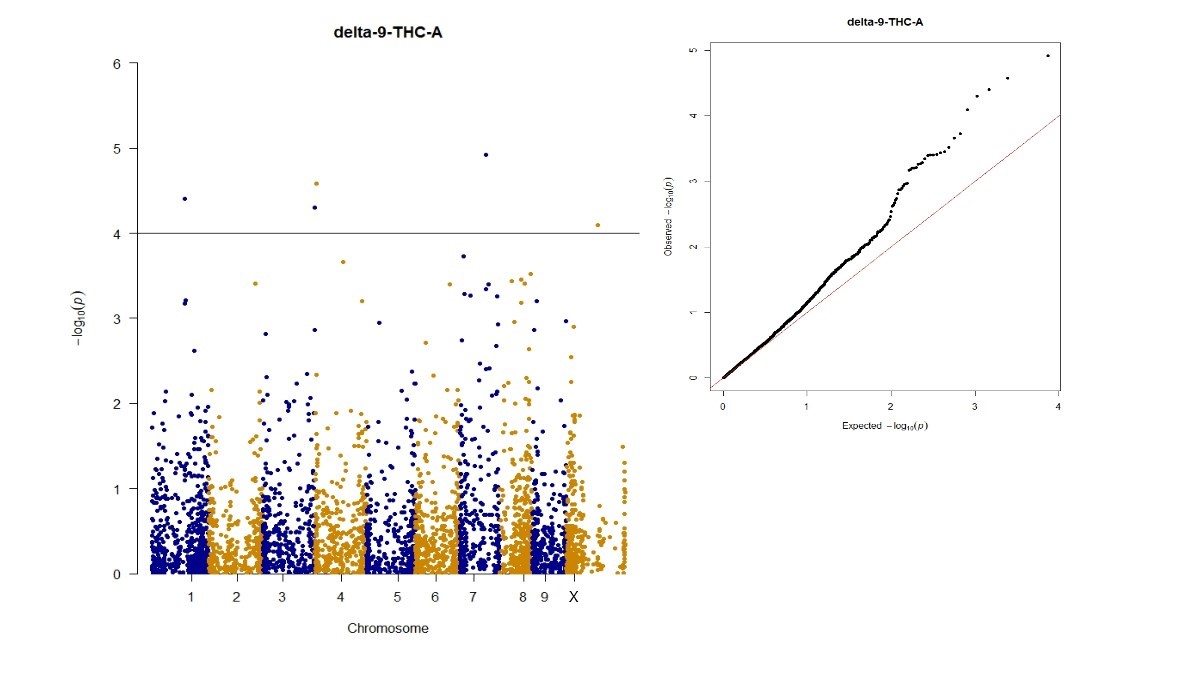

Supplement: Supplementary file 1 [file plants-15-00202-s001.zip › Manhattan plots/Figure.S27.d-9-THCA.jpg]

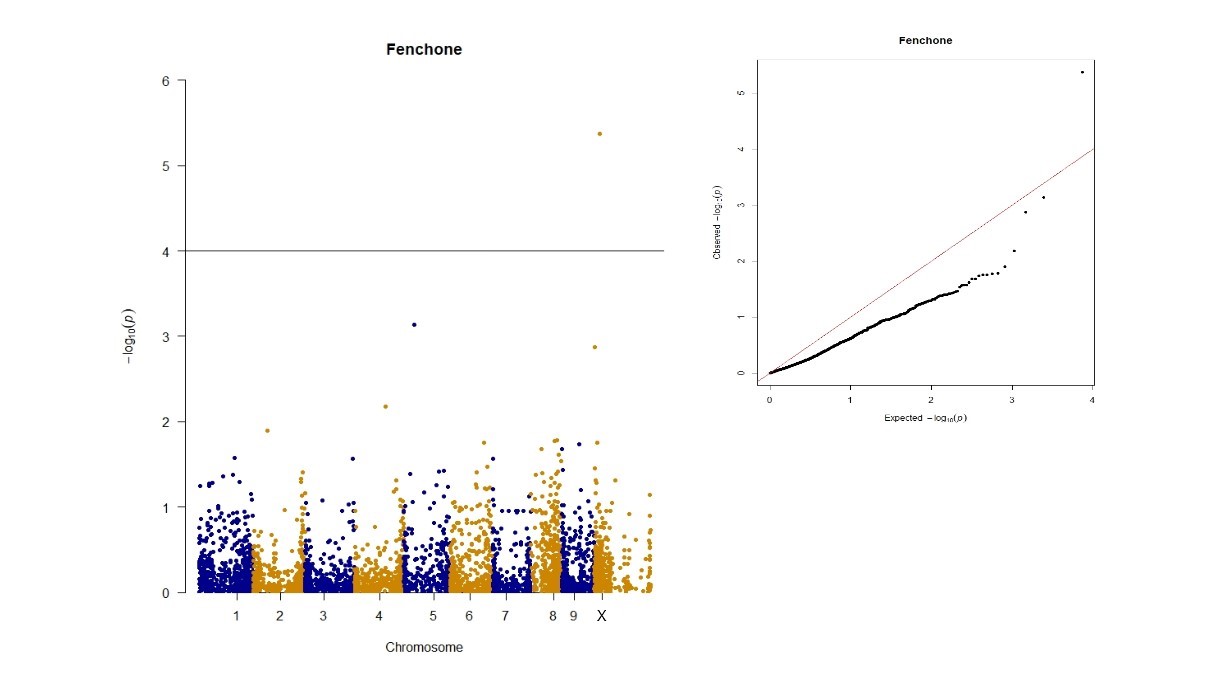

Supplement: Supplementary file 1 [file plants-15-00202-s001.zip › Manhattan plots/Figure.S28.Fenchone.jpg]

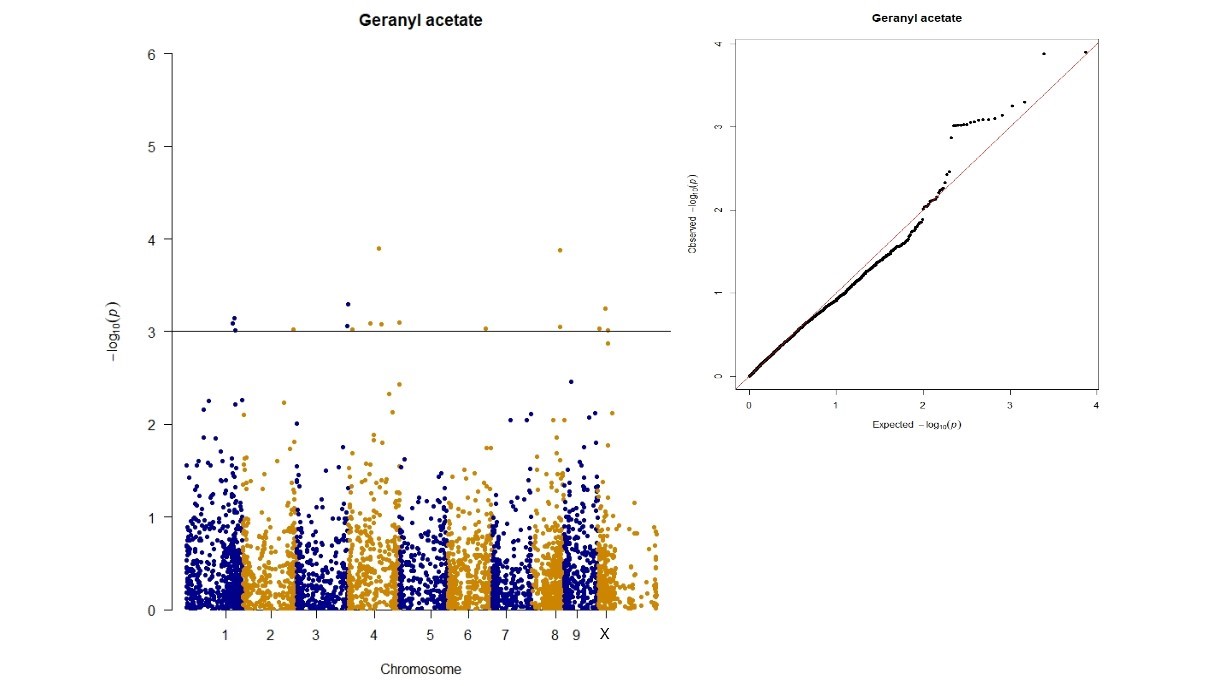

Supplement: Supplementary file 1 [file plants-15-00202-s001.zip › Manhattan plots/Figure.S29.Geranyl acetate.jpg]

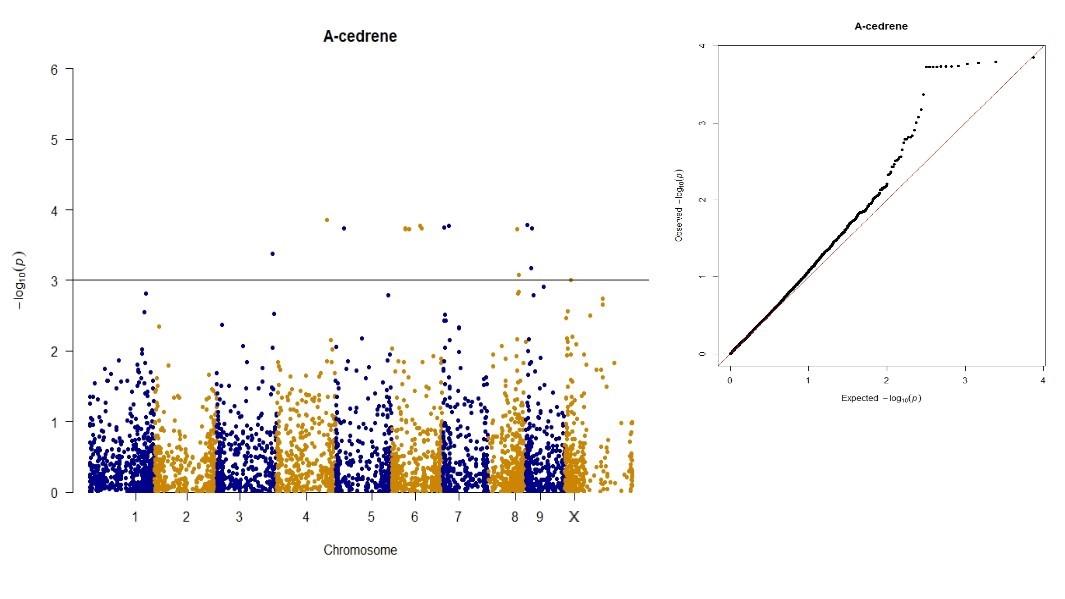

Supplement: Supplementary file 1 [file plants-15-00202-s001.zip › Manhattan plots/Figure.S3.A-cedrene.jpg]

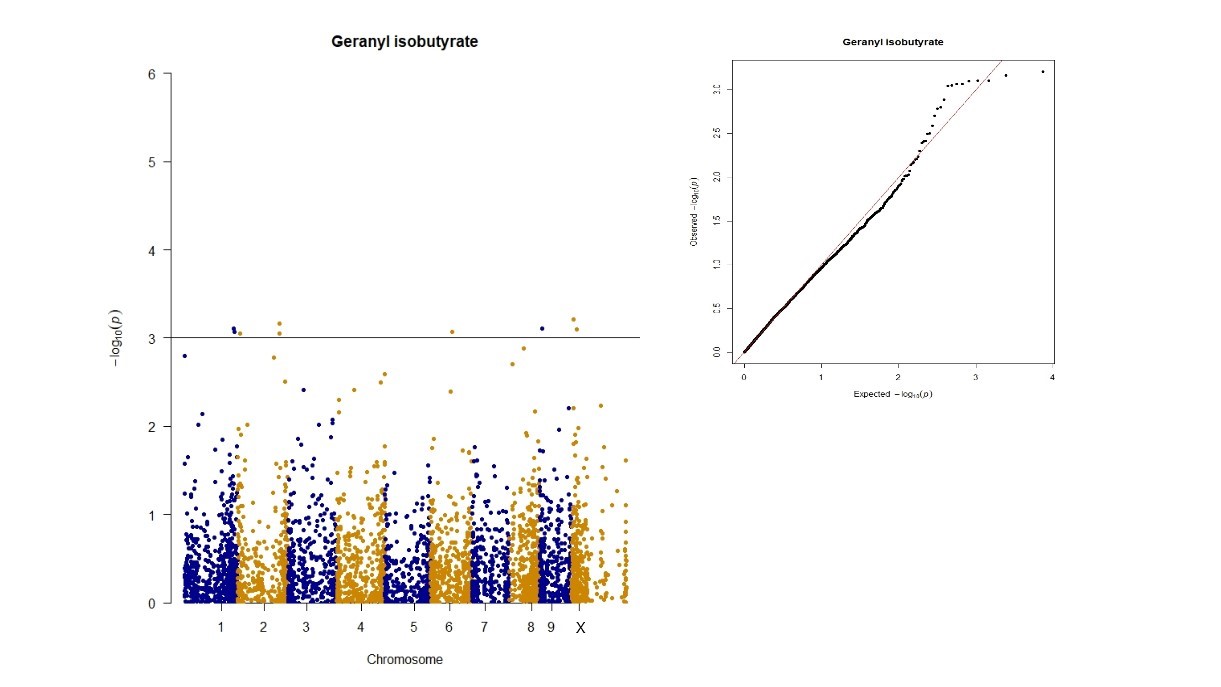

Supplement: Supplementary file 1 [file plants-15-00202-s001.zip › Manhattan plots/Figure.S30.Geranyl isobutyrate.jpg]

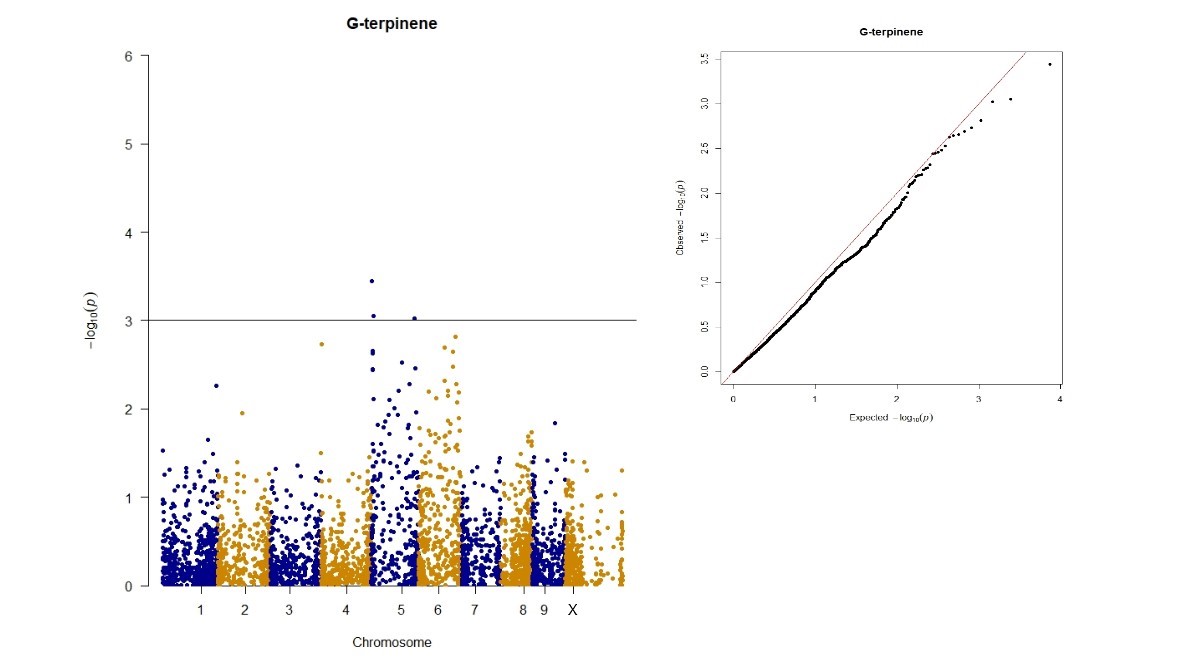

Supplement: Supplementary file 1 [file plants-15-00202-s001.zip › Manhattan plots/Figure.S31.G-terpinene.jpg]

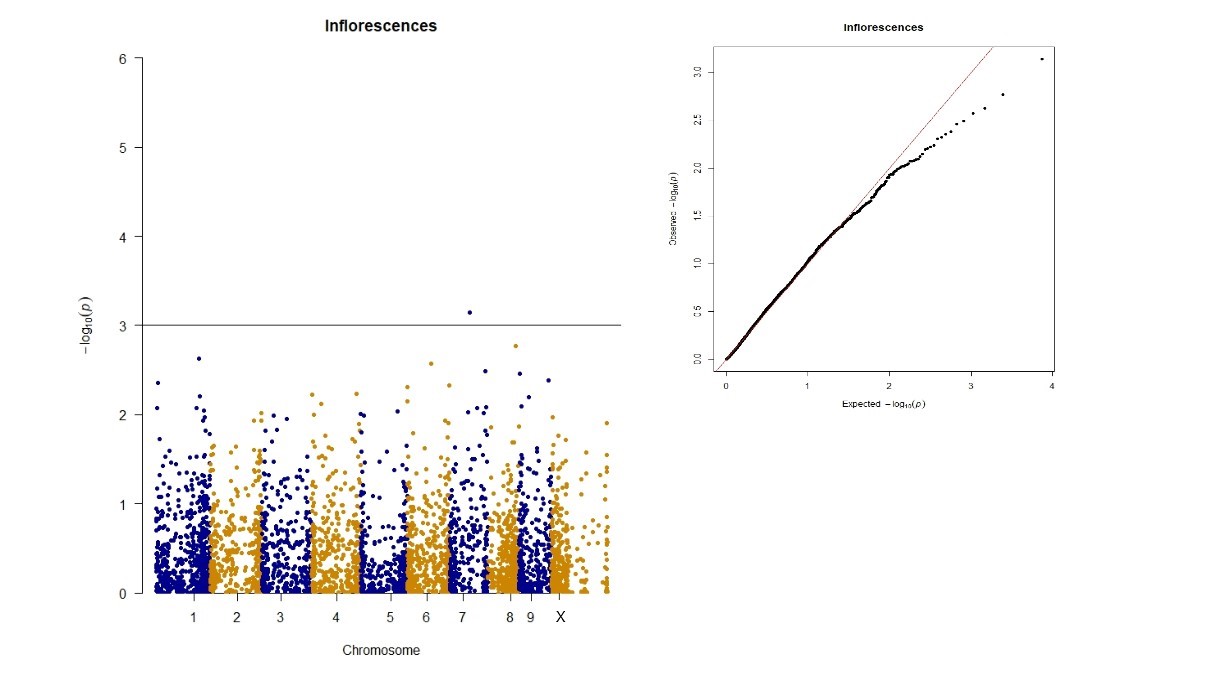

Supplement: Supplementary file 1 [file plants-15-00202-s001.zip › Manhattan plots/Figure.S32.Inflorescence.jpg]

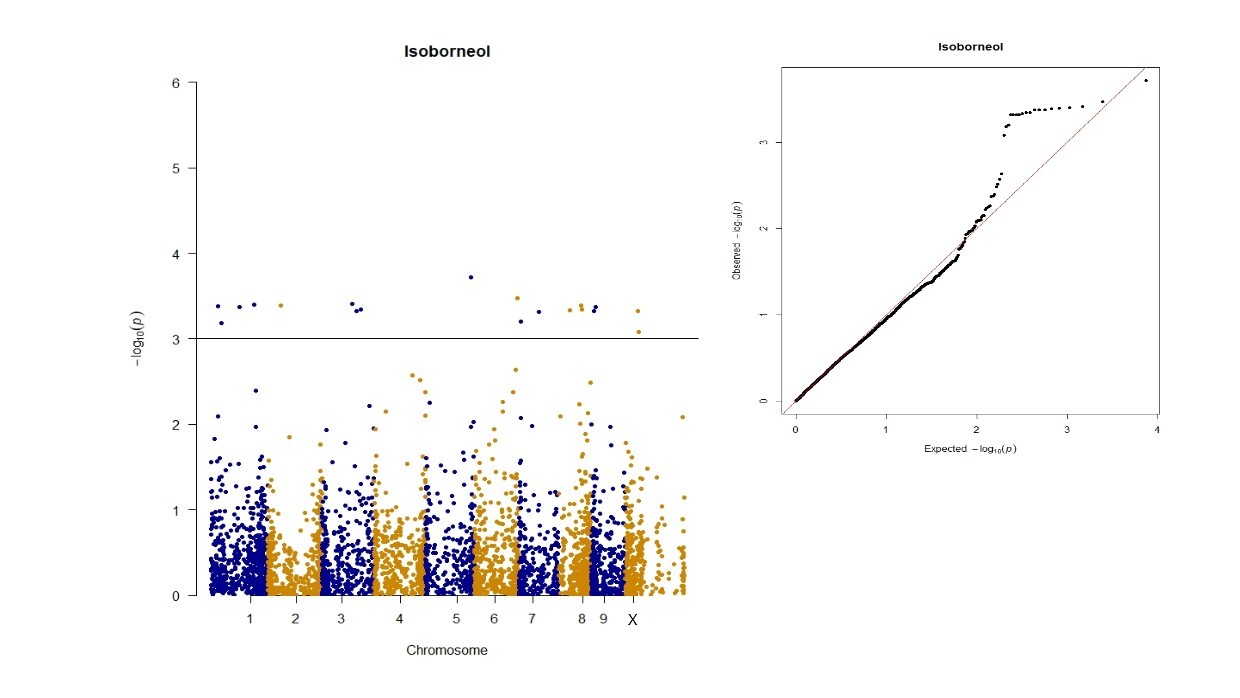

Supplement: Supplementary file 1 [file plants-15-00202-s001.zip › Manhattan plots/Figure.S33.Isoborneol.jpg]

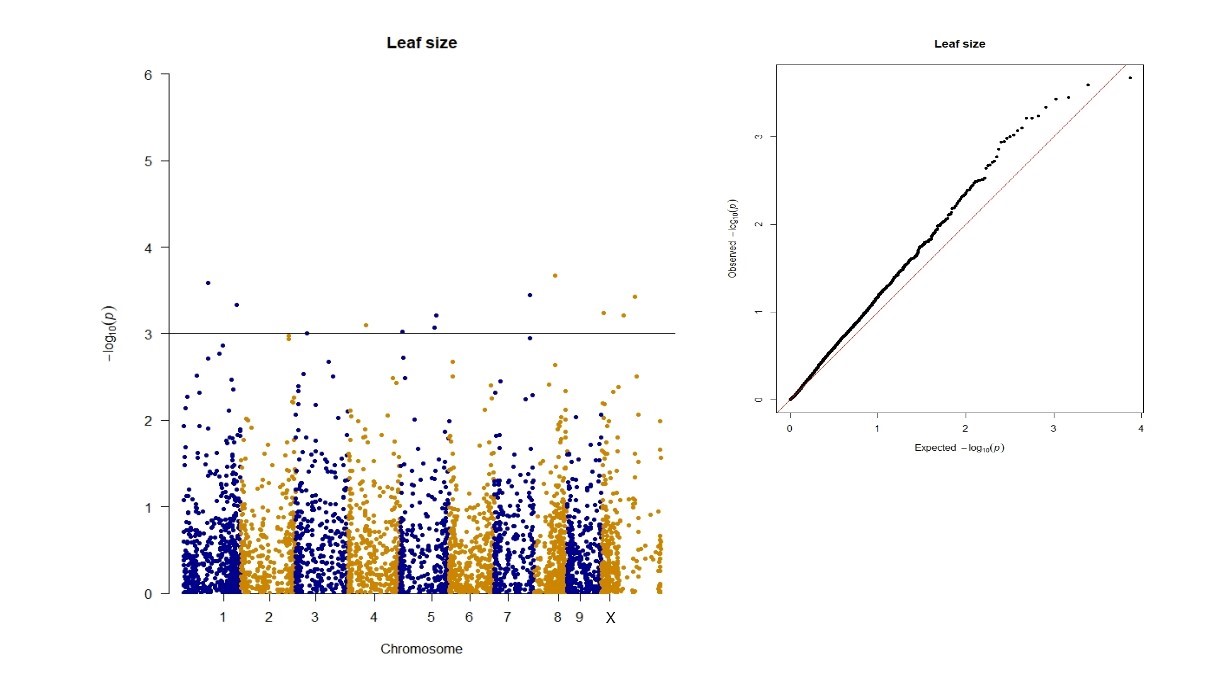

Supplement: Supplementary file 1 [file plants-15-00202-s001.zip › Manhattan plots/Figure.S34.Leaf-size.jpg]

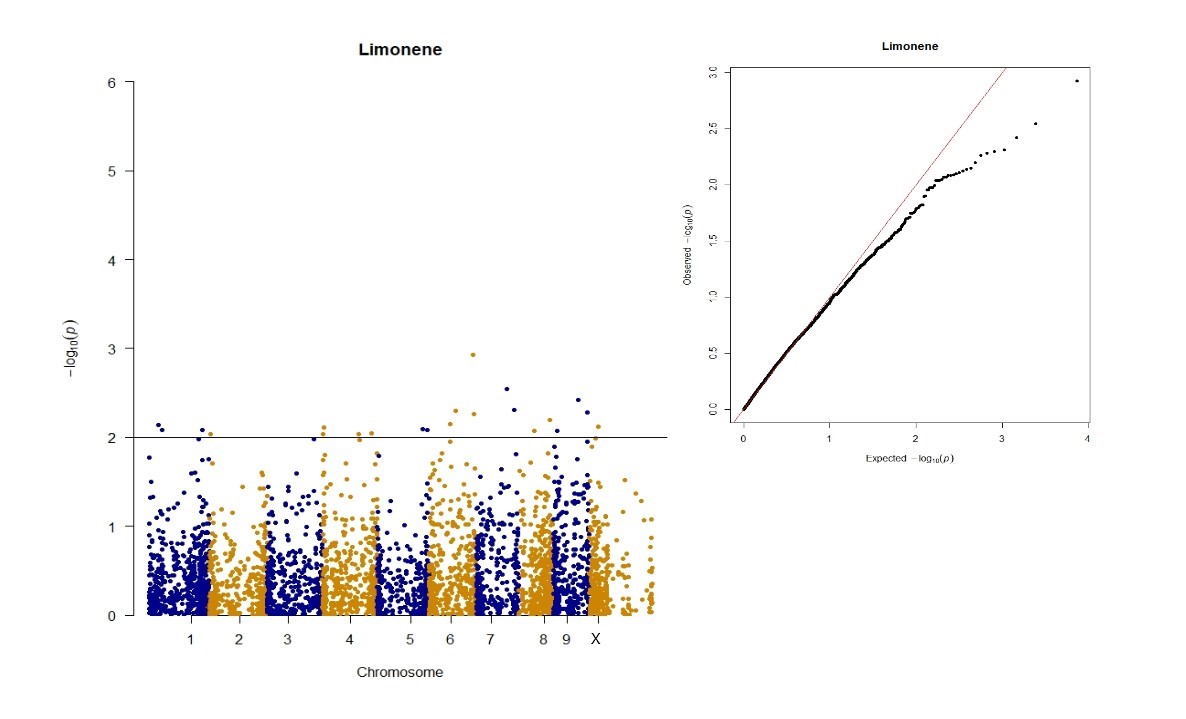

Supplement: Supplementary file 1 [file plants-15-00202-s001.zip › Manhattan plots/Figure.S35.Limonene.jpg]

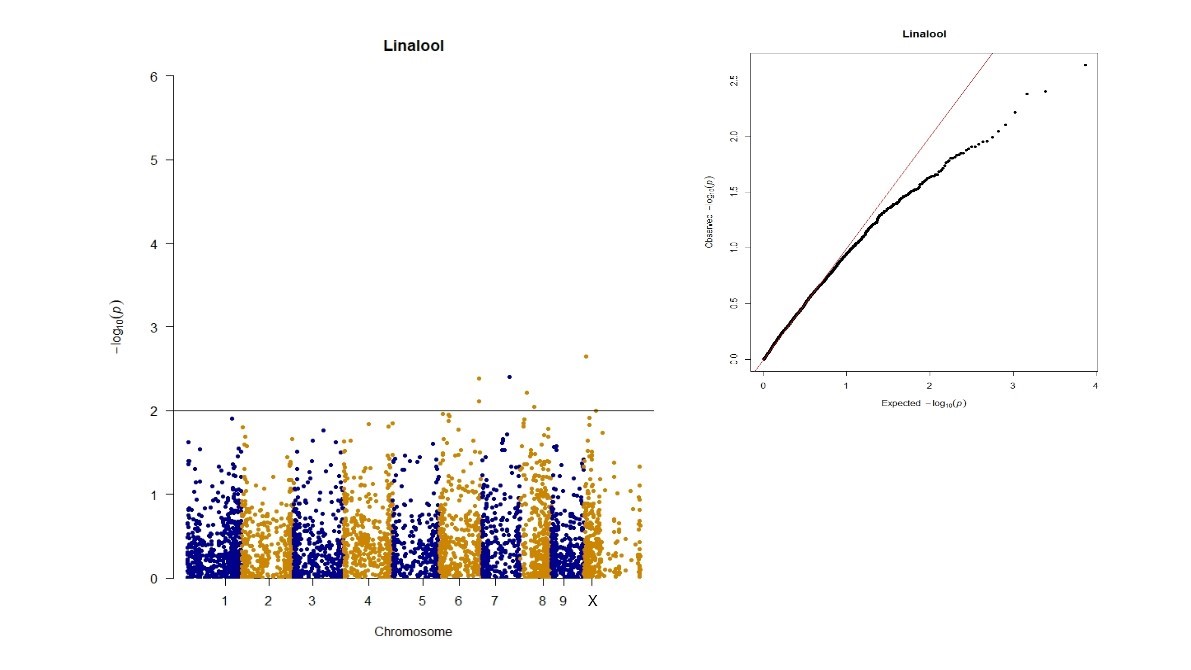

Supplement: Supplementary file 1 [file plants-15-00202-s001.zip › Manhattan plots/Figure.S36.Linalool.jpg]

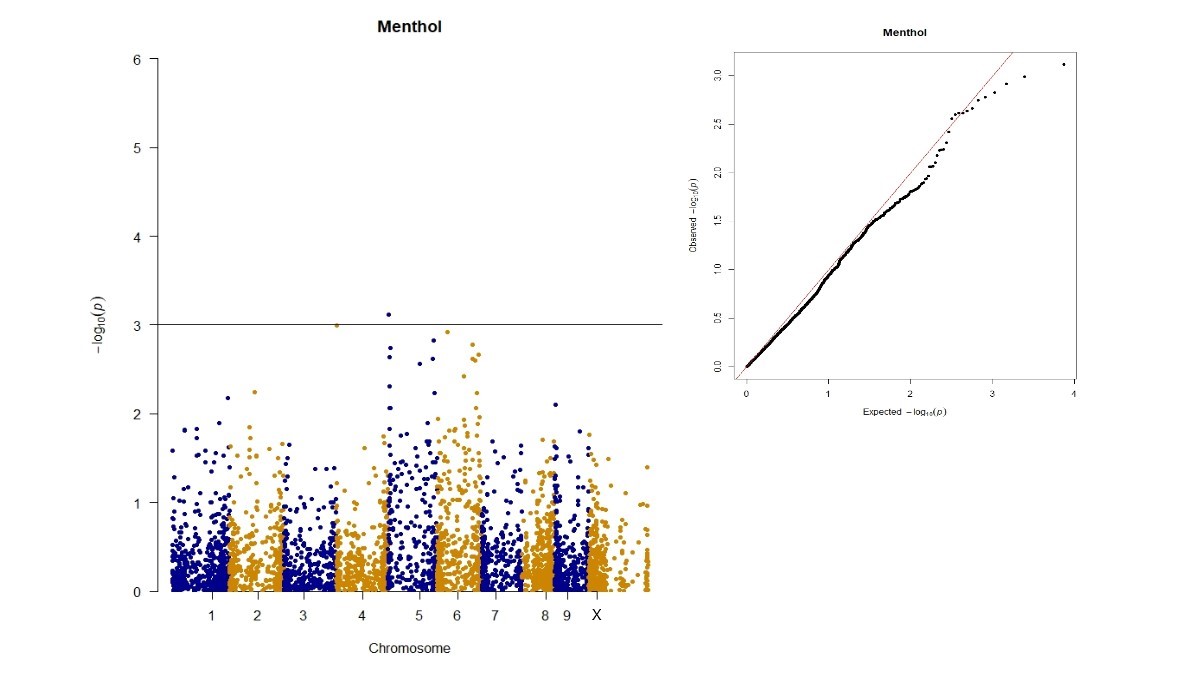

Supplement: Supplementary file 1 [file plants-15-00202-s001.zip › Manhattan plots/Figure.S37.Menthol.jpg]

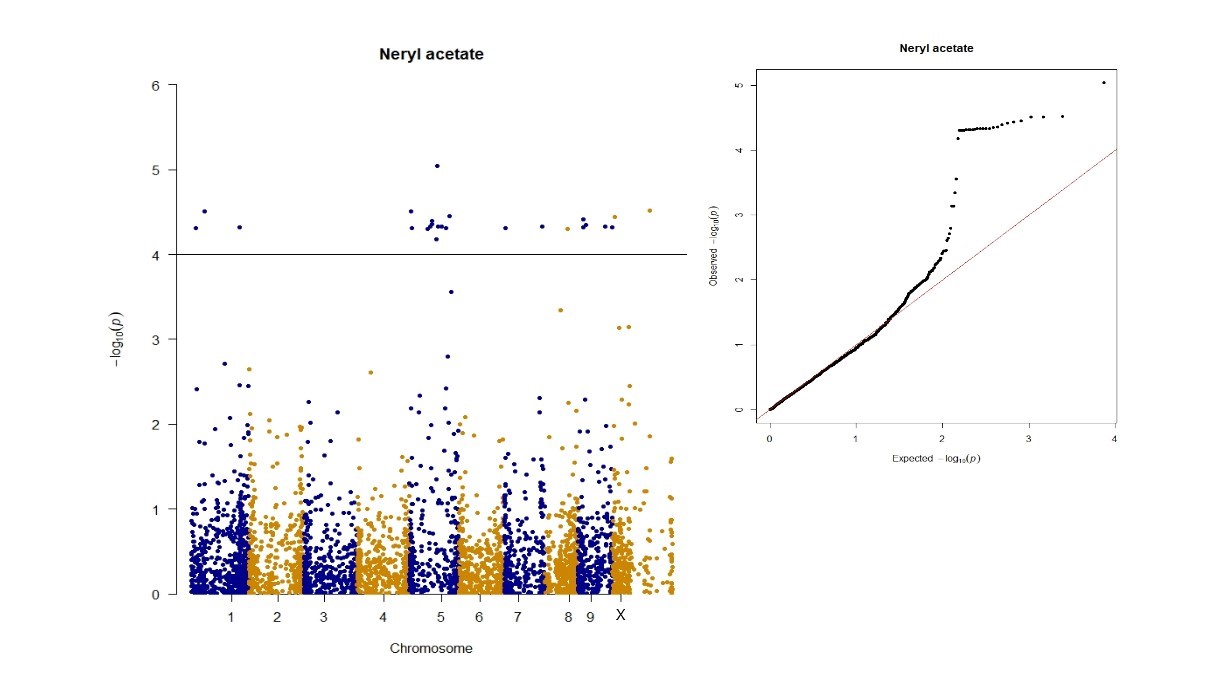

Supplement: Supplementary file 1 [file plants-15-00202-s001.zip › Manhattan plots/Figure.S38.Neryl acetate.jpg]

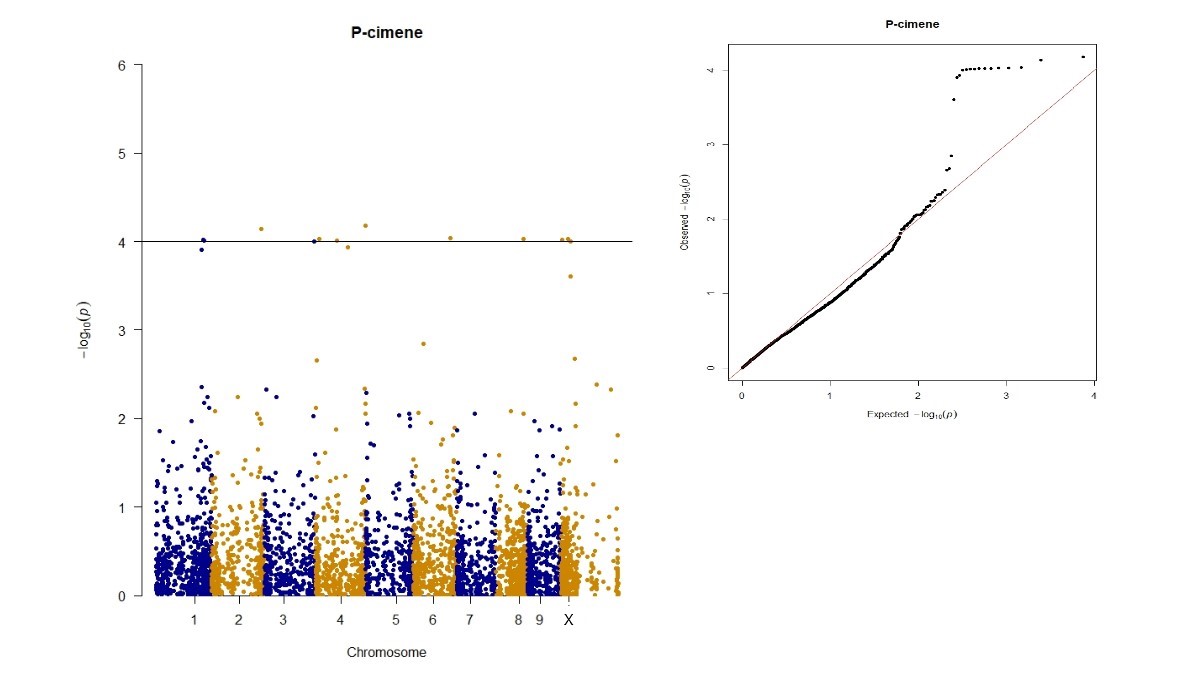

Supplement: Supplementary file 1 [file plants-15-00202-s001.zip › Manhattan plots/Figure.S39.P-cimene.jpg]

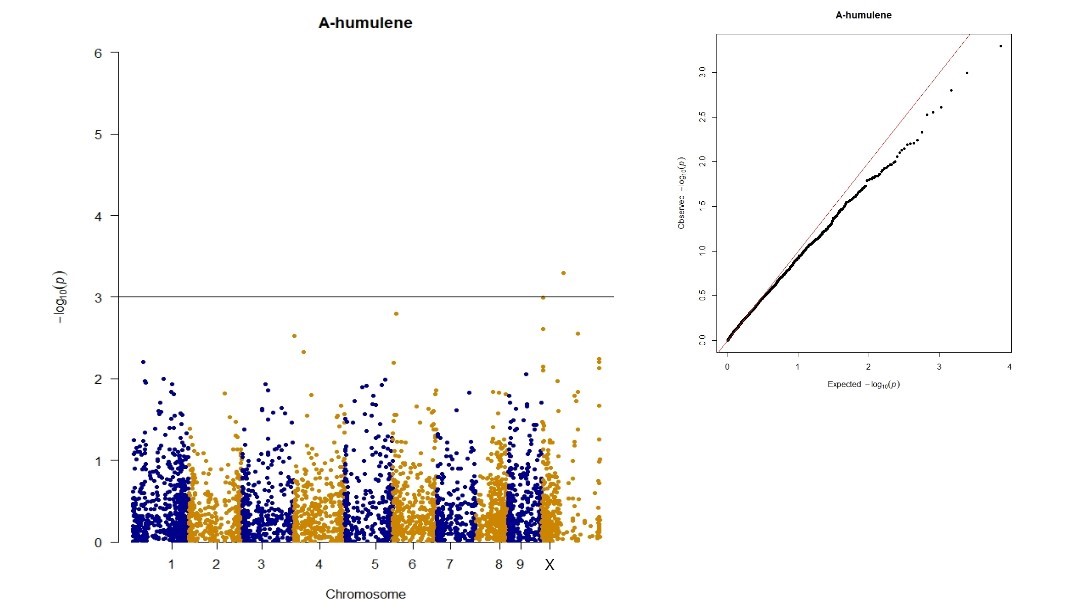

Supplement: Supplementary file 1 [file plants-15-00202-s001.zip › Manhattan plots/Figure.S4.A-humulene.jpg]

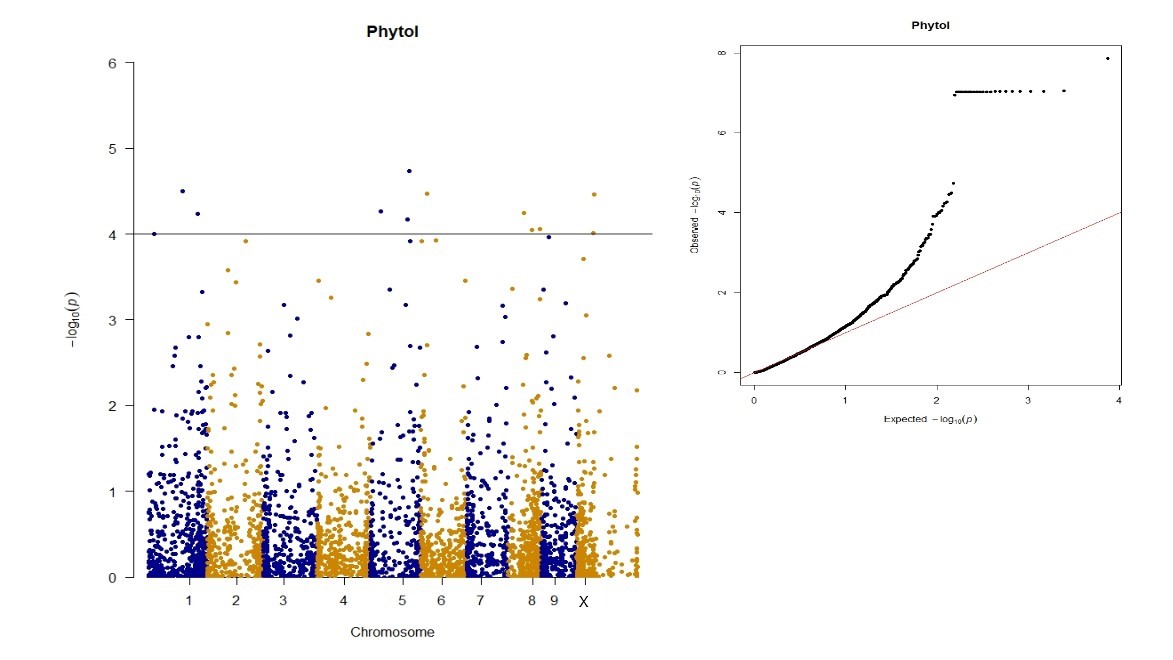

Supplement: Supplementary file 1 [file plants-15-00202-s001.zip › Manhattan plots/Figure.S40.Phytol.jpg]

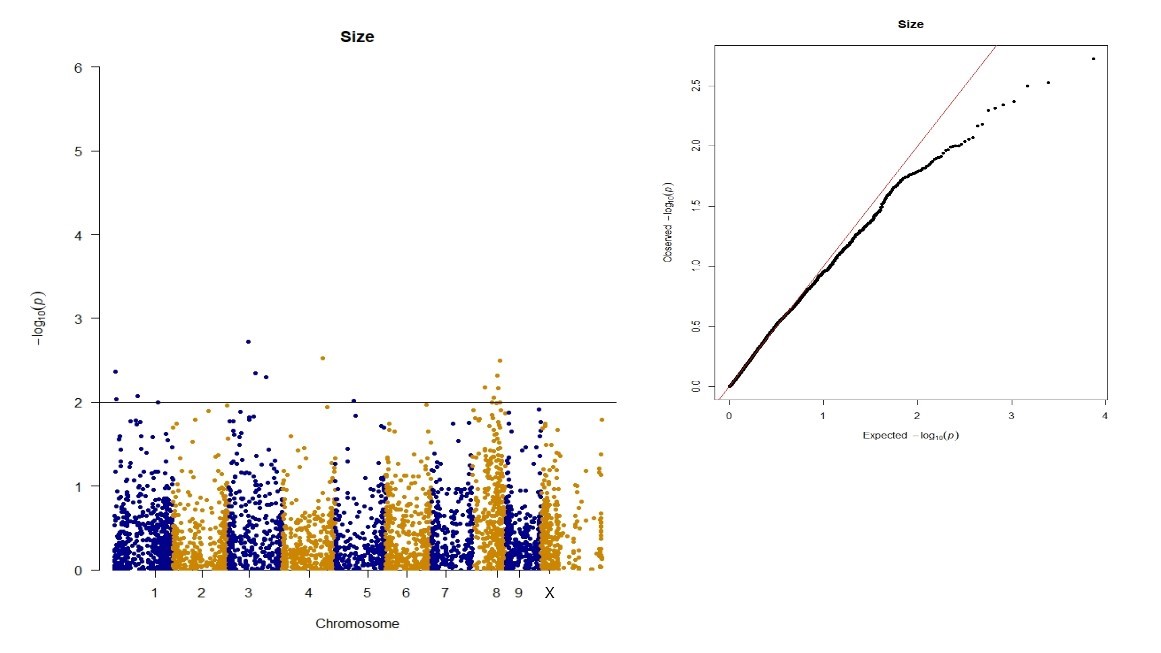

Supplement: Supplementary file 1 [file plants-15-00202-s001.zip › Manhattan plots/Figure.S41.Size.jpg]

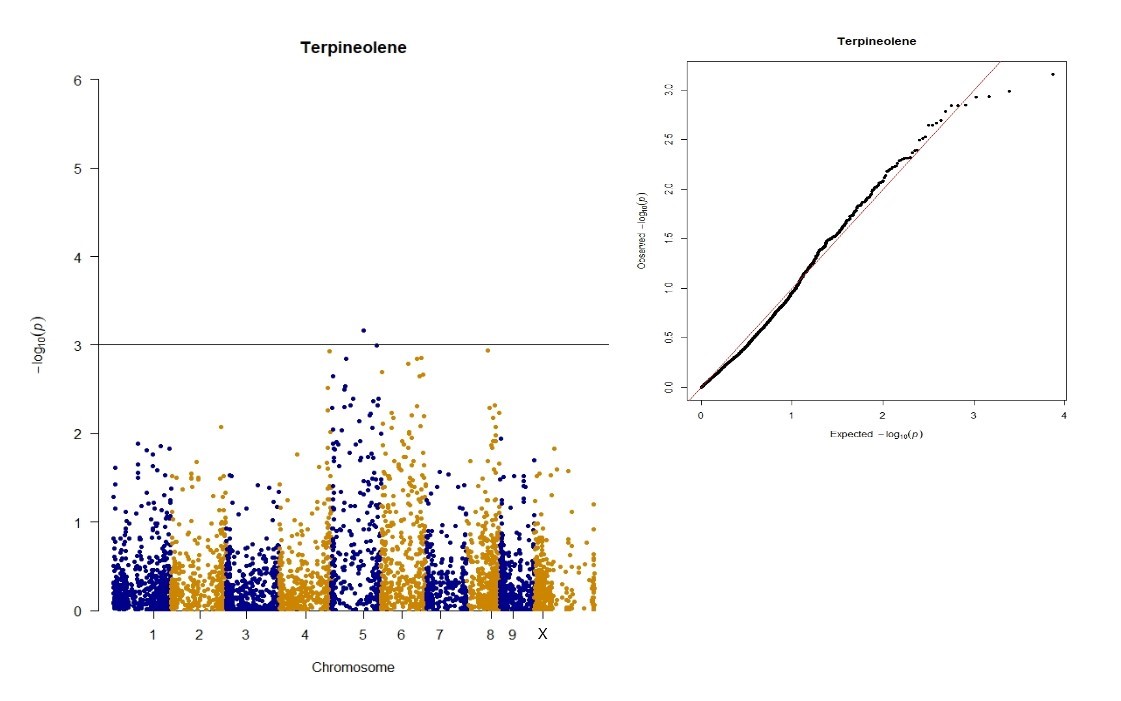

Supplement: Supplementary file 1 [file plants-15-00202-s001.zip › Manhattan plots/Figure.S42.Terpineolene.jpg]

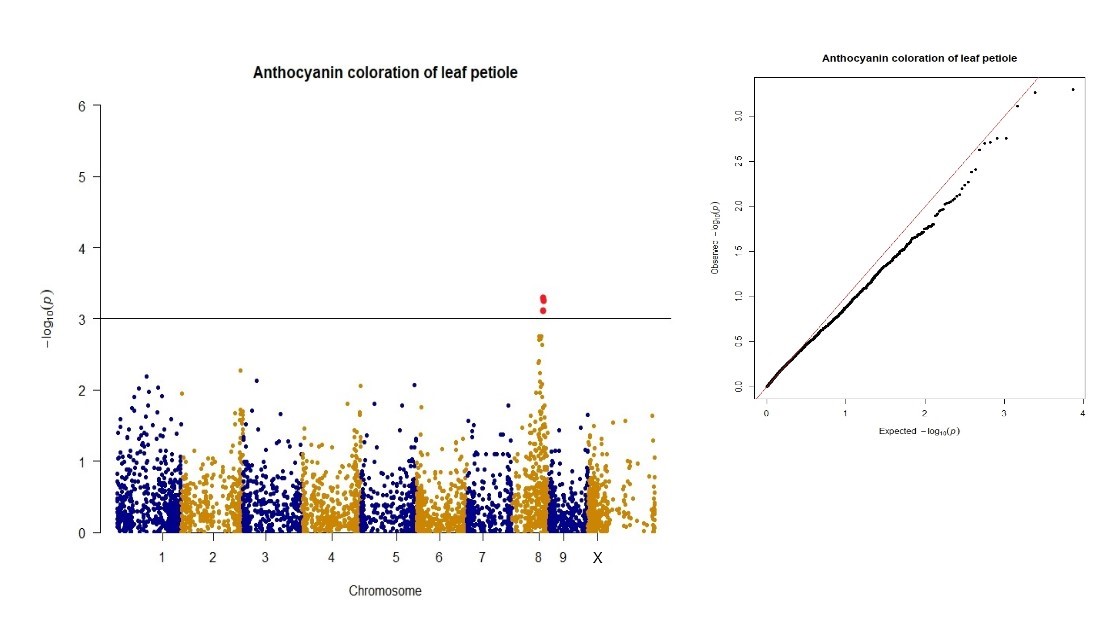

Supplement: Supplementary file 1 [file plants-15-00202-s001.zip › Manhattan plots/Figure.S5.Anthocyanin-coloration.jpg]

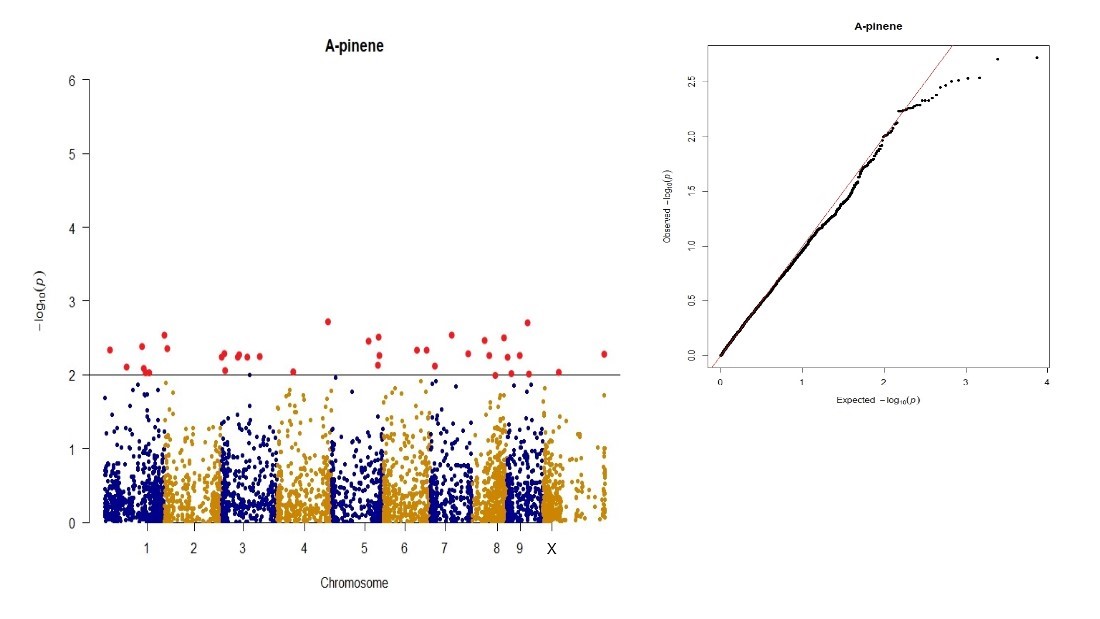

Supplement: Supplementary file 1 [file plants-15-00202-s001.zip › Manhattan plots/Figure.S6.A-pinene.jpg]

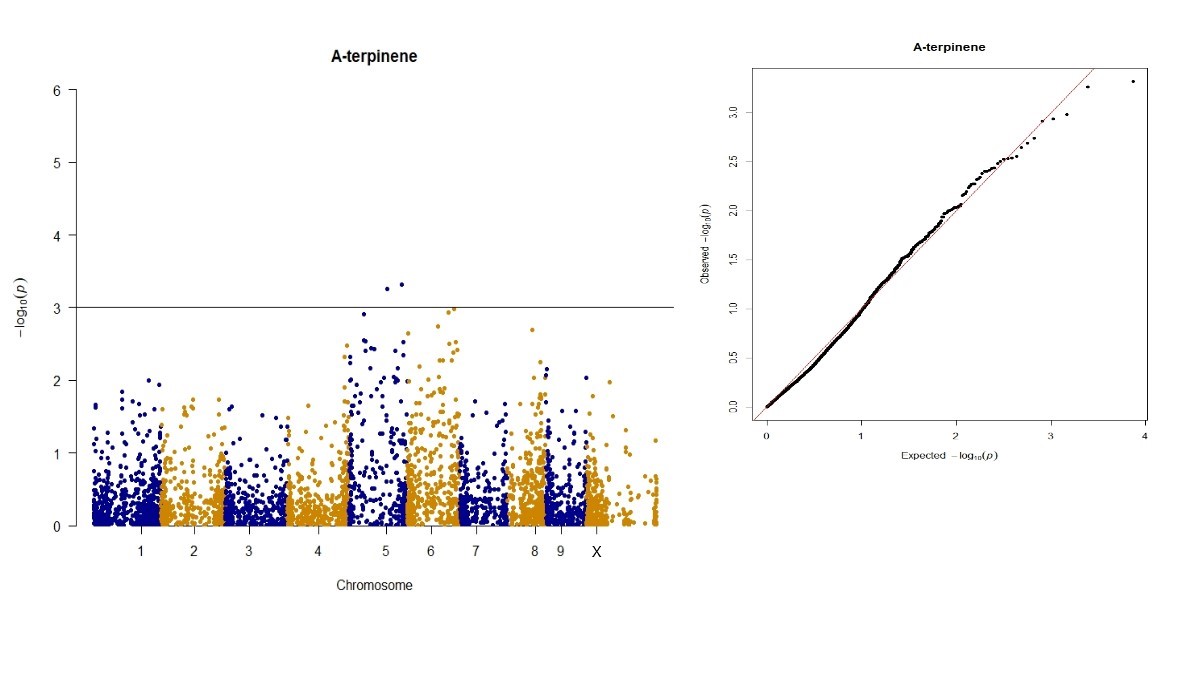

Supplement: Supplementary file 1 [file plants-15-00202-s001.zip › Manhattan plots/Figure.S7.A-terpinene.jpg]

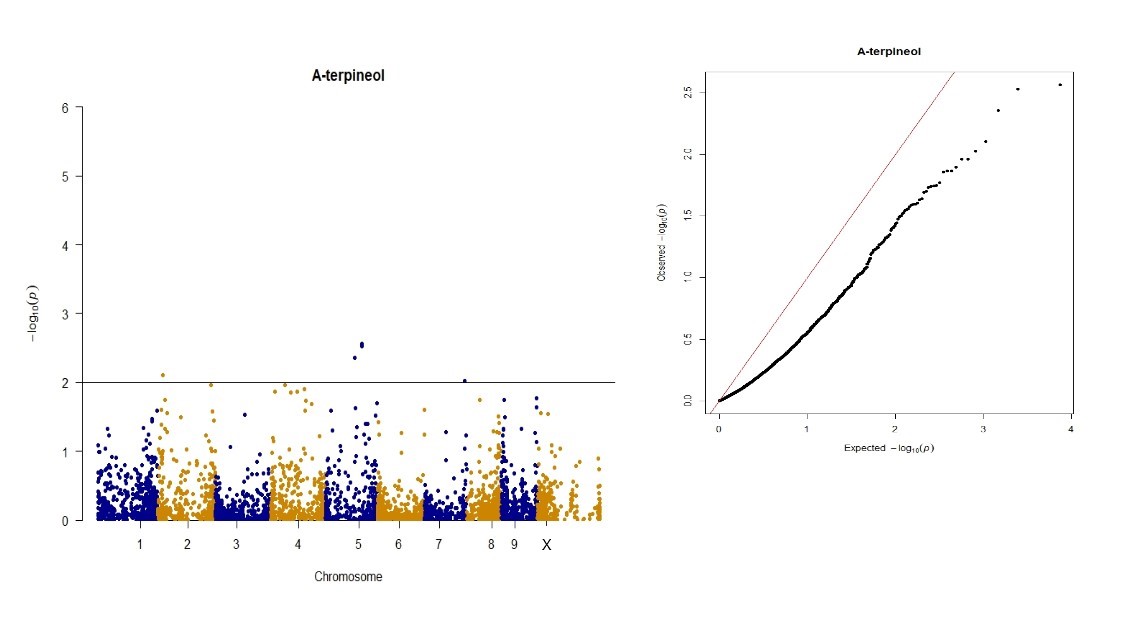

Supplement: Supplementary file 1 [file plants-15-00202-s001.zip › Manhattan plots/Figure.S8.A-terpineol.jpg]

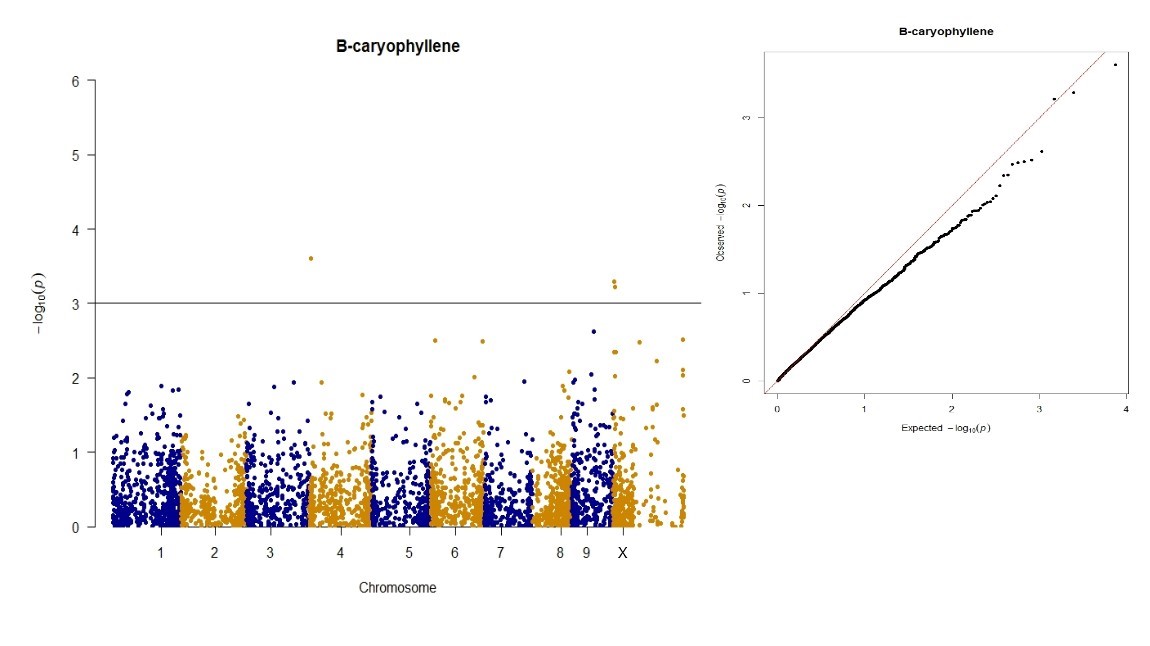

Supplement: Supplementary file 1 [file plants-15-00202-s001.zip › Manhattan plots/Figure.S9.B-caryophyllene.jpg]
